# Easy Access to Vinylene-Linked Conjugated Homopolymers from Phosphonates via an O_2_‑Mediated Aldehyde-Free Strategy

**DOI:** 10.1021/acs.orglett.5c03195

**Published:** 2025-09-13

**Authors:** Arindam Nandy, Banchhanidhi Prusti, D. Krishna Rao, Manab Chakravarty

**Affiliations:** † Department of Chemistry, 209298 Birla Institute of Technology and Sciences, Pilani-Hyderabad Campus, Jawahar Nagar, Hyderabad 500078, India; ‡ Tata Institute of Fundamental Research Hyderabad, 36/p Gopanpally, Hyderabad 500046, India

## Abstract

Among numerous approaches, Horner–Wadsworth–Emmons (HWE) reactions between phosphonates and aldehydes have become crucial for developing synthetic strategies for fully vinylene-linked conjugated 1D/2D co/homopolymers. Herein, we report room-temperature synthesis of various fluorescent, chemo/photostable 2D (porous), and 1D homopolymers via base/oxygen-mediated HWE reactions starting with tris/bis-phosphonates without using aldehyde precursors. In addition, such polymerization produces a soluble polymer with an excellent number-average molecular weight (*M*_n_ = 69 891 Da). Water-soluble phosphate salt formation as a side product is desirable.

## Graphical abstract

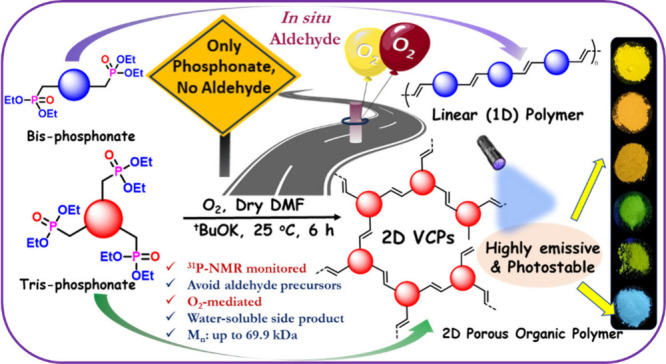

Superior conjugation in chemically and thermally stable vinylene-linked conjugated polymers (VCP) is in high demand for various real-world applications.^1–3^ Specifically, the optoelectronic properties of such materials^4–6^ are in enormous demand in the development of stimulus-responsive materials for efficient optical sensing.^7,8^ Moreover, the demand for such organic emitters is praiseworthy because of the relatively better flexibility and milder toxicity of organic molecules than inorganic materials.^9–11^ However, photobleaching upon exposure to UV radiation makes it challenging for such organic emissive materials for practical applications.^12^ Among varieties of polymers, polyethylene, poly­(vinyl chloride), and polystyrene are highly renowned homopolymers, largely used for applications.^3^ Even chemically and thermally stable 2D VCP has attracted the attention of the academic and industrial communities.

Apart from radical and coordination polymerization techniques, aldol-type or Knoevenagel condensation reactions were dominantly utilized to afford 1D/2D VCPs.^11–14^ With a slight restriction on 2D VCP generation using these strategies, the HWE reactions between phosphonates and aldehydes were introduced in producing linear or 2D VCPs (Figure 1).^15^ However, such pioneering VCP formation was described under harsh reaction conditions at 120 °C for 3 days. Later, HWE reaction-based polymerization using tris-phosphonates and tris-aldehydes was reported at 0–25 °C within 18 h and established as simple but efficient polymerization method (Figure 1).^16^

**Figure 1:**
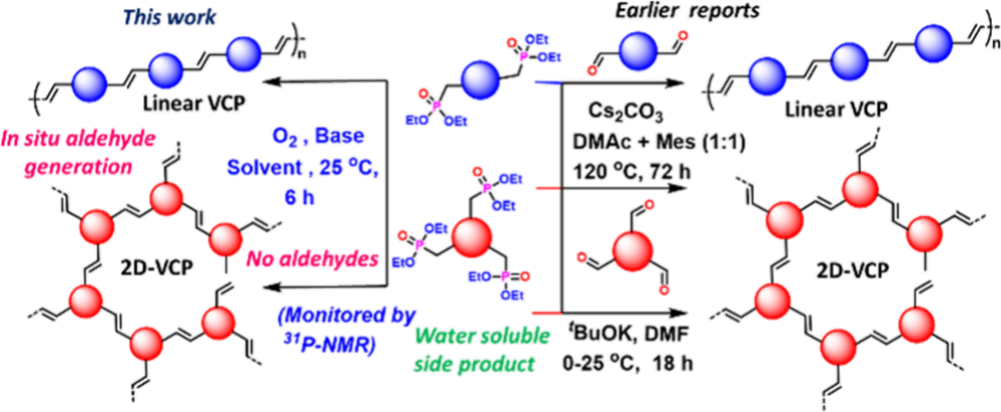
Variance between the current report and earlier reports.

Such a porous 2D VCP network, which has an excellent surface area and suitable pore size, displayed semiconducting properties and was utilized for photocatalytic hydrogen evolution from water.^16^ However, this synthetic method was employed to generate 2D VCPs, not linear polymers.^13^ Another report on HWE-based polymerization where the thiophene phosphonate was linked to aldehyde functionality was intended for self-condensation reactions, but it was a failure because aldehydes holding a carbanion became less reactive.^14^ Notably, ambient-temperature HWE dispersion polymerization was also established.^17^ Table S1 depicts systematic comparison among the O_2_-free, condensation, and O_2_-mediated polymerization approaches.

Notably, adventitious oxygen exposure during the HWE reaction of monophosphonates always led to the formation of symmetrically substituted alkenes.^18,19^ These reactions must proceed through an aldehyde intermediate, which has recently been trapped.^20^ In addition, oxidative dephosphorylation was commonly established by isolating ketones from the corresponding phosphonates, not aldehydes.^21^ The *in situ* aldehyde generation has herein reinforced the idea of synthesizing homopolymers using only bis- or tris-phosphonates by applying similar reaction conditions. Such a route would be more beneficial because many such aldehydes need a tedious synthetic procedure and also suffer from poor stability.

Initially, an easily accessible tris-phosphonate **TPP** was picked for the O_2_-mediated synthesis of homopolymer **CPN-3** (**TPV**), which was previously reported by reacting **TPP** with the corresponding aldehyde.^16^ Herein, an *N,N-*dimethylformamide (DMF) solution of **TPP** was treated with weakly nucleophilic base KO^*t*^Bu (9 equiv), and the flask was kept under a normal O_2_ balloon, generating a precipitate at 25 °C within 1 h (Scheme S1). The precipitate was washed with dichloromethane, acetone, and ethanol to remove unreacted starting material and reaction-produced oligomers and aldehydes, if any. The precipitate was primarily characterized by FT-IR and ss-^13^C NMR (Figure 2a–c), indicating fruitful polymerization. These reactions occur at 25 °C, indicating that they are driven kinetically, not thermodynamically. Hence, the formation of a well-structured/polycrystalline covalent organic framework (COF) is mostly ruled out under such reaction conditions.^22–25^

**Figure 2:**
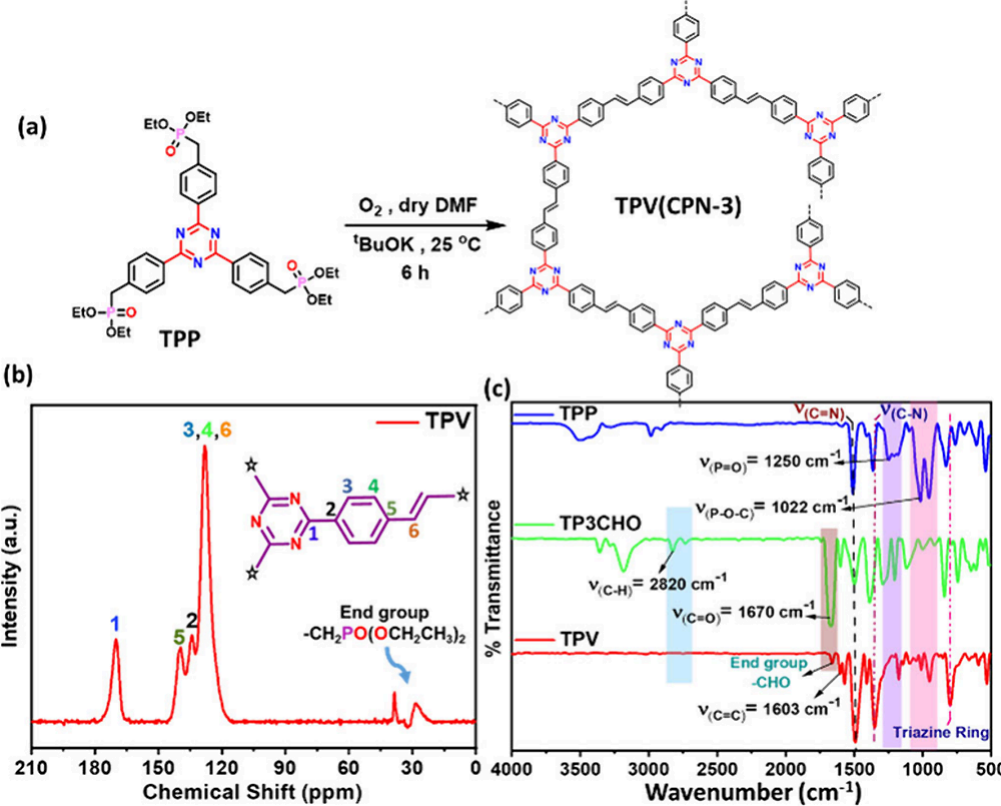
(a) Tris-phosphonate conversion to **TPV**. (b) ss-^13^C NMR spectra. (c) FT-IR comparison of phosphonate (**TPP**), an aldehyde intermediate (**TP3CHO**), and a polymer (**TPV**).

Later, this reaction was monitored through ^31^P NMR of the reaction mixture at different intervals, signifying the reaction’s completion after only 6 h (Figure 3).

**Figure 3:**
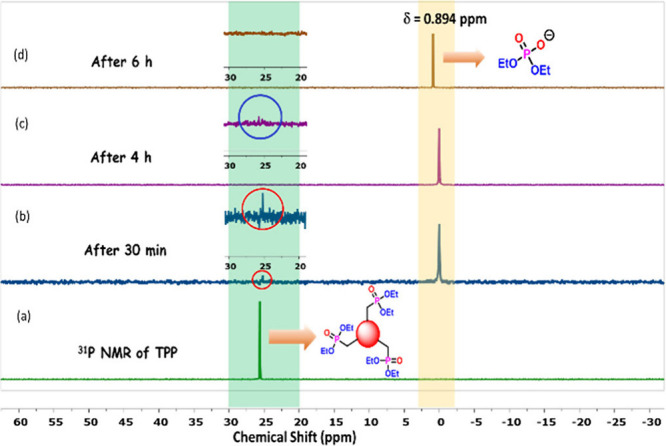
Monitoring the reaction using ^31^P NMR (CDCl_3_, 162 MHz): (a) precursor **TPP**; and **TPP** after treatment with 9 equiv of KO^*t*^Bu under an oxygen balloon in a DMF solvent for (b) 30 min, (c) 4 h, and (d) 6 h.

The phosphonate precursor appeared at δ 25.6 (Figure S1), which was drastically diminished within 30 min [∼5% unreacted phosphonate (Figure S2)], and a new signal at δ 0.894 indicates water-soluble phosphate salt formation. The estimated unreacted phosphonate is 0.9% after 4 h (Figure S3), which completely disappeared after 6 h (Figure S4). In addition, the effect of this polymerization using diverse bases and solvents was examined to optimize the yield at 25 °C within 6 h (Table S2). The reaction was smooth in the KO*^t^* Bu/DMF system, and the outcomes dictate **TPV** polymer formation in 75% yield (isolated). Although Cs_2_CO_3_ played a critical role in favorable HWE reactions,^2,15^ our attempt was unsatisfactory. The smaller number of equivalents (5–8) of KO*^t^* Bu produced polymer yields of up to 65%, and the best yield of 75% was obtained from 9 equiv of KO*^t^* Bu.

Next, the same conditions were utilized with bis-phosphonates, which produced 4,4′-biphenyl-linked linear (1D) homopolymer **BPV** in 80% yield (Scheme S2). Later, linear polymers **AMV** (1,4-dimethoxybenzene-linked) and **ABV** [2,2′-bis­(heptyloxy)-1,1′-biphenyl-linked] were conveniently produced from bis-phosphonates **AMP** and **ABP**, respectively (Schemes S3–S7). All of these polymers (Figure 4) were precipitated from the solution and purified by washing with solvents. In addition, long ethoxy chains were introduced into phosphonate **AEP** to enhance hydrophilicity. However, soluble polymer **AEV** was formed along with the expected intermediate bis-aldehyde (Schemes S8–S10), which was isolated and characterized (Figures S5–S7). Homopolymer **AEV** was isolated in 50% yield. In addition, the option of sequestering unreacted phosphonate within the porous **TPV** is ruled out by ss-^31^P NMR (Figure S8a). Also, the ^31^P NMR spectra of the reaction mixture revealed 100% conversion of **AEP** to soluble polymer **AEV** within 4 h (Figure S8b).

**Figure 4:**
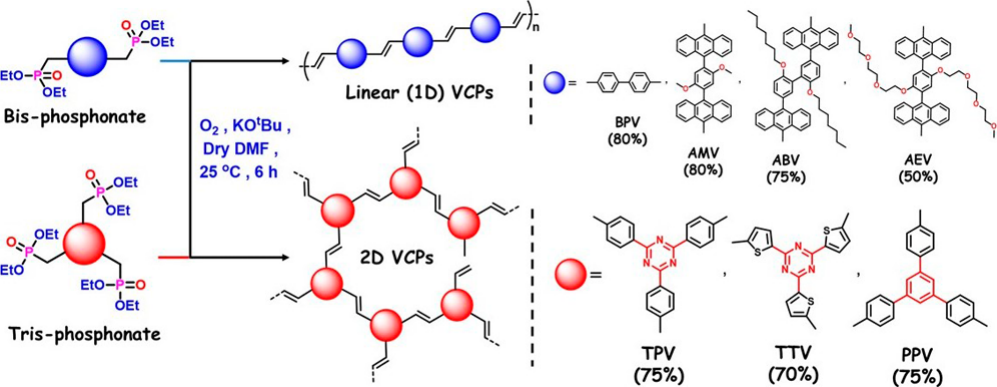
Oxygen-mediated synthesis of 1D and 2D VCPs. The yields are calculated based on the starting phosphonate amount.

Next, two more *C*_3_-symmetric phosphonates, **TTP** (thiophene-linked) and **PPP** (phenyl-linked), were synthesized (Schemes S11–S13) and indeed transformed effortlessly into the respective 2D VCPs **TTV** and **PPV**, respectively (Schemes S14 and S15). Synthesis of porous polymeric materials with a triazene core is a focus because of their potential applications.^26–28^ The molecular structures of these homopolymers were elucidated by FT-IR, ss-^13^C NMR, and X-ray photoelectron spectroscopy (XPS). As stated above, 2D homopolymer **TPV** (**CPN3**) was first synthesized and completely characterized because a similar homopolymer was formerly reported via the HWE reaction between phosphonate and aldehyde.^16^ The FT-IR spectrum of as-synthesized **TPV** shows the characteristic signal at 1600 cm^–1^ for the newly generated vinyl C=C bond, which was reconfirmed by the strong signal in the Raman spectrum (Figure 5a). The FT-IR bands from P=O and P–O–C bond vibrations from the phosphonate almost disappeared after polymerization, indicating phosphonate consumption and a higher degree of polymerization. The ss-^13^C NMR spectra displayed the vinylenic C and aromatic carbon signals at δ 128–140, and the signal at δ 170 was ascribed to the triazine ring. The powder X-ray diffraction (PXRD) profiles of **TPV** disclose only a broad diffraction peak at ∼24°, a distinctive indication of the amorphous nature (Figure 5b). All of these characteristic data completely agree with the previous report (Figure S9).^16^ The XPS data with the standard binding energies (electronvolts) also justify respective elements in C–H, C=C, and C=N with their relevant valence states. Furthermore, the excellent thermal stability of these polymers is determined by using thermogravimetric analysis (TGA) under a nitrogen atmosphere. A similar TGA profile is observed for previously synthesized triazine-based homopolymer **TPV**. Field emission scanning electron microscopy (FE-SEM) depicts microscale morphologies of the materials, matching well with the earlier reports on the HWE reaction-based **TPV** synthesis (Figure S10).

**Figure 5:**
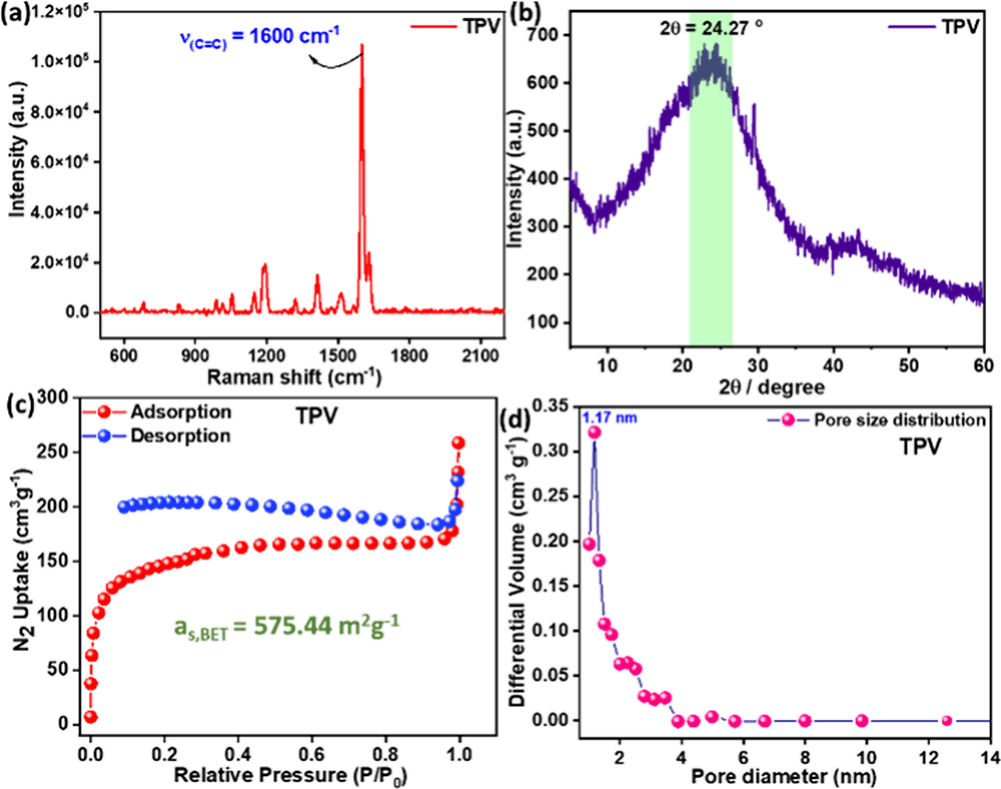
**TPV** polymer characterization: (a) Raman spectra, (b) PXRD pattern, (c) BET adsorption isotherm, and (d) pore size distribution.

The Brunauer–Emmett–Teller (BET) surface area (Figure 5c) and the microporous nature of the **TPV** were determined using nitrogen adsorption–desorption isotherms and revealed a larger BET surface area, reaching 575.44 m^2^/g, compared to the earlier report that stated 142 m^2^/g for a similar polymer synthesized via the conventional HWE route. The pore size distribution of **TPV** is demonstrated to be 2.12 nm, indicating a mesoporous nature (Figure 5d).

Subsequently, we compared the synthetic strategies for the new 2D VCP **TTV** using (i) an aldehyde-free O_2_-mediated method and (ii) a conventional HWE protocol that needs expensive aldehyde precursors (Schemes S16 and S17). The characterization data of thiophene-linked homopolymer **TTV** harmonized extremely well (Figure S11), indicating the efficacy of this direct method utilizing only phosphonates without using any aldehyde precursor. Notably, this **TTV** homopolymer, synthesized from both methods, appeared to be crystalline based on the PXRD profile, possibly due to the repetition of the chemical structure, displaying a large degree of conformational regularity, enabling the chains to align and pack together.^29^ The SEM images of this polymer also exhibit a platelike morphology (Figure S11e). Even almost identical TGA profiles (Figure S11d) were notable. Also, energy dispersive X-ray (EDX) analysis and elemental mapping validated the distribution of C, N, and S in **TTV** (Figures S12 and S13), and XPS profiles were also almost indistinguishable (Figure S14).

Subsequently, phenyl-rich 2D VCP, i.e., **PPV**, was synthesized conveniently in 75% yield following this established aldehyde-free strategy and well-characterized (Figures S15 and S16). The BET surface area of **PPV** reaches 280.5 m^2^/g.

The linear 1D VCPs are also documented by various characterization methods (Figure 6 and Figures S17–S27). The phosphonates were mostly consumed in all cases, and the new alkene–C–C stretching appeared at ∼1600 cm^–1^. The aromatic C’s at δ ∼120–140 were identified in ss-^13^C NMR for **BPV**, while anthracene and alkoxy groups appeared in ss-^13^C NMR spectra for **AMV** and **ABV**. However, the presence of end group phosphonates [(OEt)_2_P=O] was identified through weak signals in ss-^13^C NMR and FT-IR spectra for some of these polymers.

**Figure 6:**
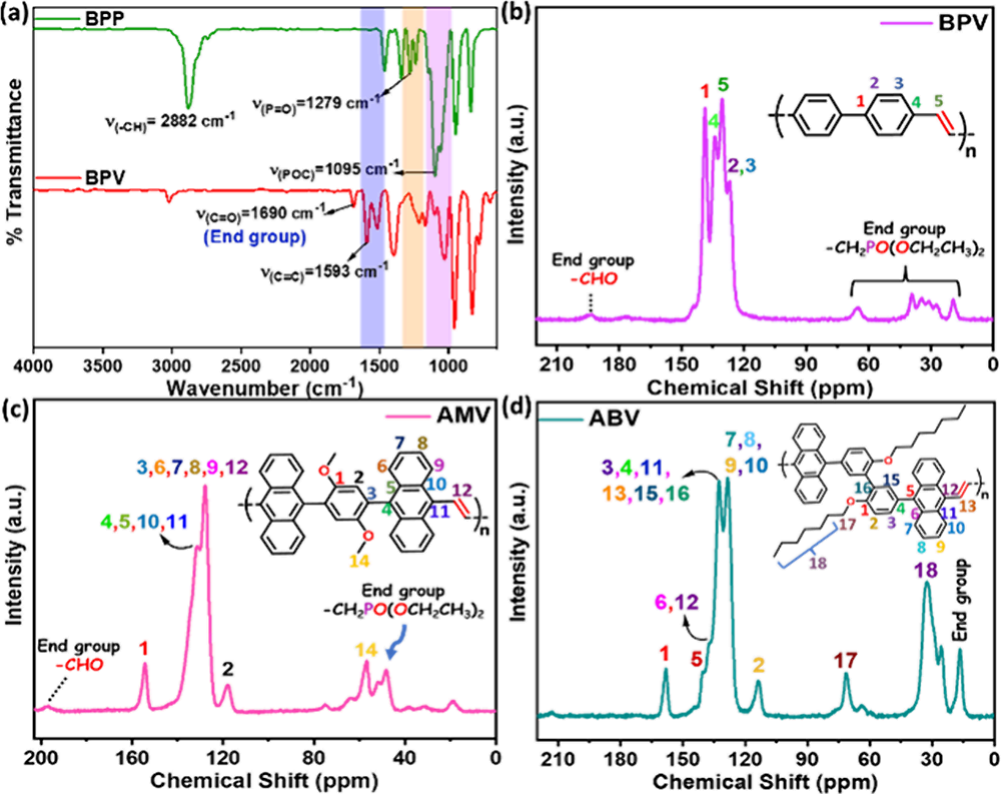
(a) FT-IR spectra of **BPP** and **BPV** and ss-^13^C NMR spectra of (b) **BPV**, (c) **AMV**, and (d) **ABV**.

To determine the degree of polymerization, a soluble polymer **AEV** (Figure 4) was produced from **AEP** and well characterized (Figures S23–S27). The gel-permeation chromatographic (GPC) analysis of the **AEV** polymer exhibited an *M*_n_ of 69 891 Da, indicating a promising degree of polymerization.

These VCPs exhibit high thermal stability (∼400 °C) and are suitable for real-life applications. An initial investigation displayed strong absorption features (Figure 7 and Figure S28), typically attributed to π–π* transitions within the conjugated backbone. Among 2D VCPs, **TTV** and **TPV** displayed better conjugations (wavelength λ_max_ = 420–430 nm) than **PPV** (λ_max_ = 398 nm) for the triazine core, enabling the donor–acceptor character. Linear polymer **BPV** absorbs at a relatively longer λ_max_ (485 nm) than **AMV** and **ABV**, indicating a better π-conjugation in **BPV** than other conformationally twisted analogues (Figure 7). These polymeric materials with optical band gaps of ∼2–2.9 eV (Figure S29 and Table S3) would be suitable for optoelectronic devices. The emission bands for 2D polymers (triazine-based) **TPV** and **TTV** appeared at λ_max_ ∼ 520 nm (green) with absolute quantum yields (ϕ_f_) of 1–3%, while **PPV** emits at λ_max_ = 457 nm (blue) with a ϕ_f_ of 13.85% (Table S4). Such a difference was elucidated by the relatively higher radiative rate constant for **PPV** (Table S5). The 1D polymers except **AEV** are yellow emissive (λ_max_ = 540–550 nm) with visually detectable ϕ_f_ values of 6–8% (Figure S30). The experimentally determined lifetime and radiative/nonradiative rate constants (Figure S31 and Tables S5 and S6) are in agreement with all of the outcomes. The slight reduction in emission intensity upon photoexposure was noticed for **TPV**, as previously reported for **CPN-3**.^16^ The rest of them showed excellent photostability (Figure S32a). In addition, the chemostability of each 1D (**BPV**) and 2D polymer (**TPV**) was tested after treatment with both 12 M KOH and HCl for 10 days, and the results were admirable, which was made evident by FT-IR studies (Figure S32b,c). Thus, such 1D/2D VCPs are useful for prospective applications.

**Figure 7:**
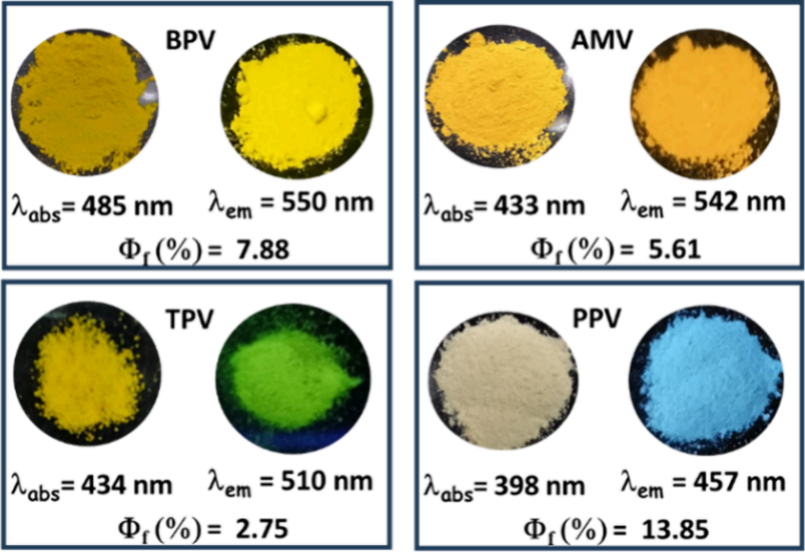
Optical features of selected 1D and 2D polymers. Photographs are taken under room light and a 365 nm lamp.

In summary, we have presented an easy base/O_2_-mediated, aldehyde-free synthetic route to access 1D and 2D vinylene-linked conjugated porous polymeric molecules with very high *M*_n_ values at 25 °C starting from phosphonates. The completion of the reaction within 6 h is monitored by ^31^P NMR and proceeds via *in situ* aldehyde formation. Water-soluble phosphate salt formation as a side product leads to easy separation of the final product. This method is established for generating porous materials with an excellent BET surface area and is superior to the conventional HWE reaction. In addition, these chemo/photostable polymers display solid-state emitting features, a suitable platform for on-site optical sensing. Also, the porous nature would enable the adsorption of crucial analytes. Thus, this newly developed protocol would serve as a new direction to access a large number of valuable 1D and 2D VCPs directly from phosphonate.

## Supplementary Material





## Data Availability

The data underlying this study are available in the published article and its .

## Notes

Details of experimental and characterization data (along with NMR and mass spectra) (PDF)
FAIR data, including the primary NMR FID files, for compounds **ABP**, **ABV**, **AEP**, **AEV**, **AMV**, **BPV**, **PPP**, **PPV**, **TPP**, **TPV**, **TTA**, **TTP**, and **TTV** (ZIP)
