# Supplementary material for: Easy Access to Vinylene-Linked Conjugated Homopolymers from Phosphonates via an O2‑Mediated Aldehyde-Free Strategy
Source: Org Lett. 2025 Sep 13;27(38):10695–9. doi: 10.1021/acs.orglett.5c03195 (PMC12481555; doi:10.1021/acs.orglett.5c03195)
Supplement: Supplementary file 1 [file ol5c03195_si_001.pdf]

Supporting Information

For

Easy Access to Vinylene-Linked Conjugated Homopolymers from Phosphonates *via* O_2-Mediated Aldehyde-free Strategy

Arindam Nandy,[a] Banchhanidhi Prusti,[a] D. Krishna Rao,[b] Manab Chakravarty[a]*

[a]Department of Chemistry, Birla Institute of Technology and Sciences-Pilani, Hyderabad Campus, Jawaharnagar, Medak, Shamirpet, Hyderabad-500078. [b]Tata Institute of Fundamental Research- Hyderabad, Gopanapally Hyderabad-500046.

Table of contents

1. Materials ---S2-S3

2. Methods, Measurements, and Instrumentations and comparative Table (Table S1)-- S3-S6

3. General experimental procedure for the polymer synthesis from monomer phosphonate-S6

4. Synthesis of monomer precursor TPP ---S7

5. Synthesis of TPV polymer --S7

6. Optimization ---S8-S10

7. Synthesis of linear polymers BPV, AMV, ABV, AEV and aldehyde intermediate --S10-S17

8. Reaction mixture studies in solid and solution state ^{31}P NMR-------------------------S17-S18

9. Synthesis of compounds TNP, TTP, and PPP---S18-S20

10. Synthesis of TTV and PPV polymer --S20-S21

11. Characterisation of TPV ---S21-S22

12. Synthesis of linker TTP and TTA --S22

13. Synthesis of TTV through novel HWE strategy --------------------------------------- S22-S23

14. Characterisation of TTV ---S23-S25

15. All characterization of PPV ---S25-S26

16. Characterisation of 1D Polymers BPV, AMV, ABV, AEV --------------------------S26-S31

17. All absorbance data --S32

18. All optical band gap plots ---S32

19. All emissions plots --S33

20. All lifetime plots and relevant tables---S33-S34

21. Photo and chemical-stability experiment --S35

22.All spectroscopic characterizations ($^{1}H/^{13}C/^{31}P$, HRMS, LCMS)---------------------S35-50

23. Supplementary References ---S50-S51

1. Materials:

All reactions were carried out in a hot air oven-dried glassware under an argon atmosphere. All the chemicals, such as 4-cyanobenzylbromide, 5-methylthiophene-2-carbonitrile, 4,4”-dimethyl-5’-(p-tolyl)-1,1’:3’,1”-terphenyl, 4,4’- bis(chloromethyl)-1,1’-biphenyl, 9-anthracenecarboxaldehyde, 1,4-dimethoxybenzene, 2,2’-bisphenol, 1,4-dihydroxybenzene were purchased from various chemical companies, 4',4''-(1,3,5-Triazine-2,4,6-triyl)tribenzaldehyde (**TP3CHO**) is commercially availed to check the IR spectra. Methane sulphonic acid,(MSA) triethyl phosphite and bases (KO*ᵗ*Bu, Cesium carbonate), NaH (60% dispersion in mineral oil) were commercially availed and was utilized without additional purification. Solvents, including *N, N*-dimethylformamide (DMF), 1,2-dichloroethane (DCE), 1,4-dioxan, *N, N*-dimethylacetamide (DMAc), dimethylsulphoxide (DMSO), tetrahydrofuran (THF, distilled after buying), Acetonitrile (CH_3CN), ethyl acetate, dichloromethane (DCM), methanol, and hexane, were purchased. Local suppliers purchased oxygen and argon cylinders. Column chromatography was performed using Silica gel 100-200 mesh. Reactions were monitored by thin-layer chromatography on a precoated silica gel 60 F254 plates (Merck &

Co.) and were visualized by UV (mainly 365 nm). The reference used in NMR is TMS. Chemical shifts are reported in ppm, and multiplicities are indicated by s (singlet), d (doublet), t (triplet), and dd (doublet of a doublet). High-resolution mass spectra (HR-MS) were recorded on an Agilent 6538 UHD Q-TOF electron spray ionization (ESI) mode. All data were plotted using Origin 2021 software. Gel Permeation Chromatography (GPC)-analysis: M_n, Mw and Mz were measured by size exclusion chromatography (SEC) on a Malvern VISCOTEK TDA 305 equipped with an absorbance detector (UV, λ = 254 nm), and three polystyrene gel columns based on a conventional calibration curve using eight polystyrene standards. THF was used as a carrier solvent at a flow rate of 1.0 mL/min.

2. Methods, Measurements, and Instrumentations:

Solution ^1H, ^{13}C, ^{31}P NMR data: The solution state ^{1}H, ^{13}C, ^{31}P NMR data were recorded on a Bruker 400 MHz instrument (400 MHz for ^{1}H NMR and 101 MHz for ^{13}C NMR). Copies of ^{1}H, ^{31}P, and ^{13}C NMR spectra can be found at the end of the Supporting Information. Coupling constants (J) were reported in Hz. The reference used in NMR is TMS. Chemical shifts are reported in ppm, and multiplicities are indicated by s (singlet), d (doublet), t (triplet), and dd (doublet of a doublet). The solvent signals were used as references ($CDCl_3$: ^{1}H = δ 7.26 & ^{13}C = δ 77.00; DMSO: ^{1}H = δ 2.50 ppm & δ ^{13}C = 39.52 ppm). All SS-NMR experiments were conducted on a JEOL spectrometer (Model: JNM-ECZL600G, JEOL Ltd., Japan) equipped with a 3.2 mm MAS double (HFX) resonance probe operating at resonance frequencies of 150.9 MHz for ^{13}C, and 242.9 MHz for ^{31}P. NMR experiments are conducted at room temperature unless stated otherwise

Fourier Transform Infrared (FT-IR) Spectroscopy measurement: All the FT-IR spectra were recorded with Jasco FTIR-4200, spectral range: 400-4000 cm^{-1}, and DLATGS detector.

Thermal Stability Analysis: Thermogravimetric analysis (TGA) was carried out on a Shimadzu DTG-60 simultaneous DTA-TG apparatus with an increasing temperature rate at 10 °C min^{-1} in N_2 atmosphere.

Powder X-ray diffraction measurement: PXRD measurements were carried out using a Rigaku ULTIMA IV system with a dual detector with Cu-K_α micro focus within the range of 5° to 50° at a scanning speed of 2 °/min. The sample was placed and spread over a Kapton tape, and data were recorded in transmission geometry.

Field-Emission Scanning Electron Microscopic study (FE-SEM): *T*he FE-SEM images and EDX measurement were carried out for drop-cast solution over a silicon wafer and powder over a carbon tape using the FEI Apreo LoVac instrument.

Brunauer-Emmett-Teller (BET): The powder sample was activated at 90 ˚C in the vacuum oven for 24 h. Microtrac Bel-BELSORP mini II model surface area analyzer was used to collect nitrogen adsorption isotherms, BET surface area, and pore size distribution (PDS) plot.

X-ray Photoelectron Spectroscopy (XPS): The XPS analysis was carried out using the Thermo Scientific Kalpha instrument. The source used for the XPS analysis is an Al K-α source [X-ray source 1486.8 eV]. The presence of C, N, O, and P was confirmed.

***Solid UV and Fluorescence spectroscopy measurement*:** The UV spectra in the solid state were recorded with a Jasco UV-670, and the solid-state emission spectra were recorded with Fluorolog, Horiba.

***Absolute quantum yield and lifetime decay measurement*:** The solid-state absolute quantum yield was measured using a calibrated integrating sphere method with an absolute error of ±2%. Time-resolved fluorescence measurements were performed using a time-correlated single-photon counting (TCSPC) unit (Horiba Deltaflex). The laser used for all samples was 403 nm. All measurements were performed at room temperature. The decay fitting was completed, keeping the χ^2 value close to unity.

Table S1: Comparative evaluation of the general differences in a few parameters between stated polymerization processes

Parameters	Conventional Condensation protocols	Oxygen-free polymerization	This report (Oxygen-mediated polymerization)
Reaction Conditions (temperature, time, stoichiometry, atmosphere)	**Temperature:** Typically commences at a range of temperatures, including room temperature for some systems, but elevated temperatures are often vital to initiate the reaction and remove by-products.<br>Catalysts (acids, bases, metal complexes, and enzymes) are also used for	**Temperature:** It can be performed at temperatures ranging from low (even below 0°C) to high (up to 200°C), depending on the monomer and initiator used.<br>Requires a source of free radicals (e.g., thermal decomposition of	**Temperature:** It is performed at room temperature (25 °C) in the presence of base and molecular oxygen. This reaction proceeds with only one phosphonate, no other precursors, such as an aldehyde. (Unlike the earlier report, the conventional HWE-reaction-based report).

	some reactions to promote the reaction.<br>**Time:** The stepwise growth needs a relatively longer time (slower reaction rate)<br>**Stoichiometry:**<br>Needs monomers with two or more functional groups that react to form a new bond and eliminate a small molecule<br>**Atmosphere:**<br>It doesn't require a specific atmosphere if the precursors are stable.	peroxides or azo compounds, UV light, redox reactions) to initiate the reaction.<br>**Time:** Being involved in a highly reactive free-radical-based mechanism, it is very rapid.<br>**Stoichiometry:**<br>Involves monomers with double or triple bonds that simply add to each other to form a chain without the elimination of any atoms.<br>**Atmosphere:**<br>Need an inert atmosphere to prevent oxygen's inhibitory effects.	**Time:** It takes 6-10 hours to form the polymer. It is also a condensation polymerization where water-soluble phosphate is formed as a side product. The stepwise growth needs a relatively longer time (slower reaction rate). Unlike to previous reports with 1-3 days reactions, it is relatively faster.<br>**Stoichiometry:**<br>Needs only bis-/tris-phosphonates and 3 equiv base under an oxygen balloon to get the polymers.<br>**Atmosphere:**<br>It needs molecular oxygen. The atmospheric oxygen would not be adequate to make the reaction faster. Hence O_2 atmosphere is needed. Hence, this is an oxygen-mediated polymerization reaction.
Polymer quality (molecular weight, dispersity (Mw/Mn), thermal stability, optical properties),	**Molecular weight:** Normally leads to polymers with higher molecular weights.<br>**Dispersity:** Typically, this results in polymers with lower dispersity (more uniform molecular weight distribution)<br>**Thermal stability:** Often results in polymers with strong covalent bonds between repeating units and potentially strong intermolecular forces.<br>**Optical properties:**	**Molecular weight:** Oxygen's presence can hinder the reaction, especially at the surface, leading to lower molecular weights or incomplete polymerization.<br>**Dispersity:** High (Broad molecular weight distribution:<br>**Thermal stability:**<br>Generally lower than condensation polymers, especially if the polymer has a tendency to decompose or	**Molecular weight:** Make a high molecular weight polymer within a short period of time [Determined from GPC for one of the analogous soluble polymers M_w = 71851; M_n: 69891]<br>**Dispersity:** The dispersity is close to 1 (1.03), which indicates good homogeneity and monodispersed features.<br>**Thermal stability:**<br>These vinylenic fully conjugated polymers showed thermal stability up to 400 °C.

	Condensation polymers tend to have higher molecular weights and stronger intermolecular forces, leading to increased crystallinity and potentially higher refractive indices.	depolymerize at elevated temperatures<br>**Optical properties:**<br>Radical polymers often have a more amorphous structure with lower molecular weights, resulting in lower refractive indices and potentially greater light scattering.	**Optical properties:**<br>These are amorphous with a higher molecular weight and show excellent absorption features in the wavelength range from 400-450 nm. The solid-state fluorescence nature with a wavelength range (450 to 550 nm) is also impressive.
Work-up procedures (purification efficiency, scalability, environmental impact)	**Purification efficiency:**<br>Involves the elimination of small molecules like water, often requiring more extensive purification to remove these byproducts and unreacted monomers.<br>Often necessitates techniques like vacuum processing or azeotropic distillation for efficient by-product removal<br>**Scale up:**<br>Generally easier to scale up due to its simpler reaction mechanism and fewer limitations on monomer choice.<br>**Environmental impact:**<br>Releases small molecules as byproducts, which can be relatively benign. The overall environmental impact depends on factors like monomer source, polymer disposal, and energy consumption.	**Purification efficiency:**<br>Ideally, no small molecules are eliminated during the polymerization process<br>primarily focuses on removing residual initiators and unreacted monomers.<br>Commonly employs methods like dialysis or precipitation to purify the polymer from residual monomers and solvents<br>**Scale up:**<br>Radical polymerization, while versatile, faces challenges in achieving high molecular weight and controlling polymer properties at large scales.<br>**Environmental impact:**<br>Some initiators used in radical polymerization may be toxic or produce harmful byproducts.	**Purification efficiency:**<br>Formed polymer can be easily separated through filtration. Purifications are only by washing with organic solvents to remove trapped water soluble phosphate or phosphonates.<br>**Scale up:**<br>Easier to scale up. As these phosphonates are easily accessible, Just Phosphonate /base and an oxygen balloon would produce the polymer on a large scale.<br>**Environmental impact:**<br>Releases water-soluble phosphate salt molecules as byproducts, which may not be much harmful for all but . The overall environmental impact depends on factors like monomer source, polymer disposal, and energy consumption.

3. General experimental procedure for the synthesis of polymers (TPV, TTV, PPV, ABV, AMV, BPV) from monomer phosphonate:

An oven-dried 50 mL round-bottom (RB) flask was cooled under a Schlenk line. Next, 0.1 g (1 eq.) of the respective phosphonate was well dissolved in 7 mL dry DMF under an Argon

atmosphere at room temperature, followed by the addition of KO^{t}Bu (9 eq.), and was stirred for 6 h under oxygen (balloon). The completion of the reaction was monitored through thin-layer chromatography (TLC). After the completion, the reaction was quenched with ice-cold water, and insoluble precipitates were formed, which were filtered and washed with DCM, methanol, THF, and acetone (50 mL × 3 times) to remove any unreacted monomer, oligomer, and any other impurities. The residual solid was dried under vacuum for 24 h at 60 °C to deliver a solid polymer.

4. Synthesis of monomer precursor TPP:

The compound **BNP** was synthesized using an earlier reported procedure.[1] Then, an oven-dried 50 mL two-neck RB was cooled under a vacuum Schlenk line. 0.1 g (0.197 mmol) of **BNP** was dissolved in 5 mL of chloroform. One neck was attached with an N_2 balloon, and the other was connected with a dropping funnel through which 0.0523 mL (0.591 mmol) of CF_3SO_3H was added dropwise into the RB at 0 °C. The mixture was stirred at 25 °C for 24 h, poured into ice water, and neutralized with ammonium hydroxide. The precipitate was filtered out and then washed with water and acetone several times. Then the compound was recrystallized from toluene to deliver a white solid.

Yield 72% (0.215 g), M.P. 85-90 °C; FT-IR (ν, cm^{-1}): 3493, 2984, 1509, 1364, 1251, 1206, 1017. ^{1}H NMR (in 400 MHz, $CDCl_3$) δ 8.67 (d, J = 7.76 Hz, 6H), 7.49 (dd, J = 6.28, 2.36 Hz, 6H), 4.09-4.02 (m, 12H), 3.28 (d, J = 22.12 Hz, 6H), 1.28 (t, J = 7.04 Hz, 18H). ^{13}C NMR (101 MHz, $CDCl_3$) δ 171.3, 136.6, 134.9, 130.3, 129.3, 62.5 (d, J = 6.6 Hz), 34.1 (d, J = 137.9 Hz), 16.5 (d, J = 5.9 Hz). ^{31}P NMR (162 MHz, $CDCl_3$) δ 25.6 (s). HRMS (ESI-TOF) *m/z*: $[M+H]^+$ Calcd for $C_{36}H_{49}N_3O_9P_3$ 760.2682; Found 760.2686.

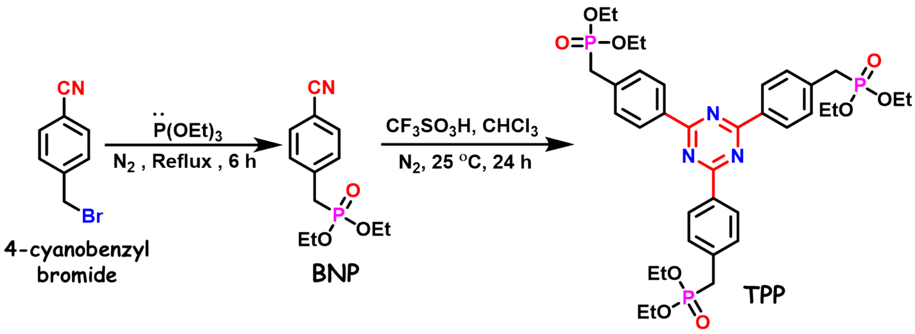

Scheme S1: Synthesis of monomer precursor **TPP**.

5. Synthesis of TPV polymer:

An oven-dried 50 mL RB was cooled under a vacuum Schlenk line. 0.1g (1eq.) of **TPP** was dissolved in 5 mL dry DMF under an Argon atmosphere at room temperature, followed by the addition of KO^{t}Bu (9eq.), and was stirred for 6 h under oxygen (balloon). The completion of the reaction was monitored through thin layer chromatography (TLC). After the completion, the reaction was quenched with ice-cold water, and insoluble precipitates were formed, which were filtered and washed with DCM, methanol, THF, and acetone (50 mL × 3 times) to remove any unreacted monomer, oligomer, and any other impurities. The residual solid was dried under vacuum for 24 h at 60 °C to deliver a yellow solid with 75% yield.

6. Optimization:

This reaction was monitored through ^{31}P NMR with different time intervals by taking the direct reaction mixture. Initially, only phosphonate was there after 30 mins of response, almost 95% of phosphonate was consumed, and after 4 h, 1% phosphonate was remaining, which was completely consumed after 6 h, as confirmed by TLC. At almost δ = 0.01 ppm, a strong peak of water-soluble phosphate salt (-P(OEt)$_2$(O)O$^-$K$^+$) was formed. Here are the ^{31}P NMR spectra of different time intervals.

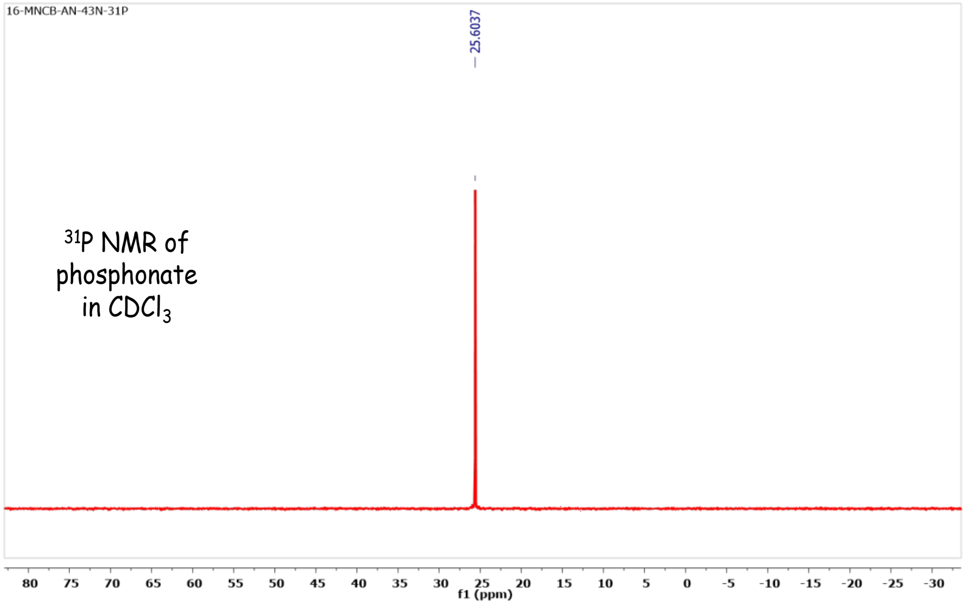

Figure S1: ^{31}P NMR (162 MHz, $CDCl_3$) of **TPP** (monomer phosphonate).

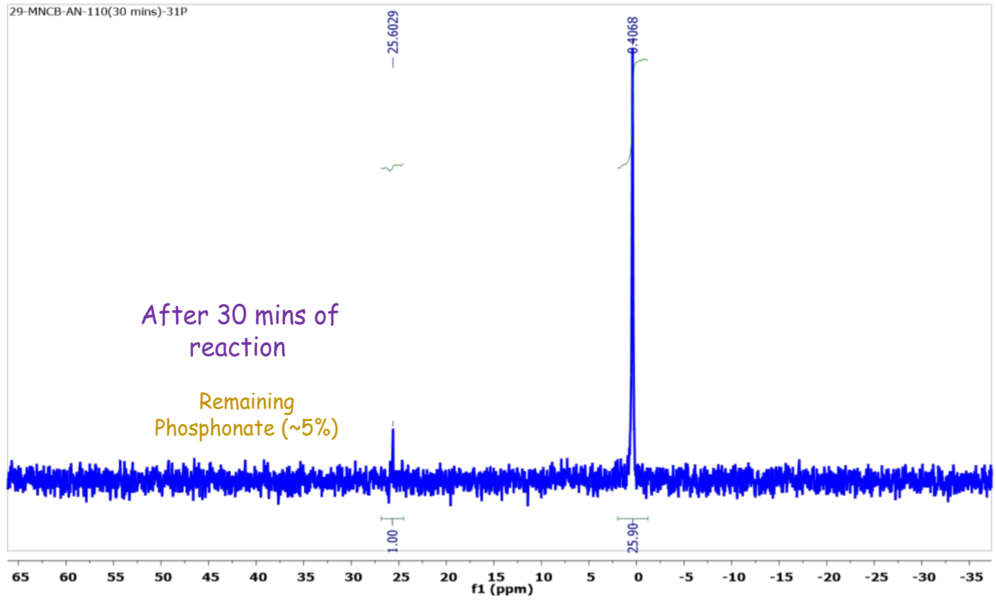

Figure S2: ^{31}P NMR (162 MHz, $CDCl_3$) of reaction mixture after 30 minutes of reaction progress.

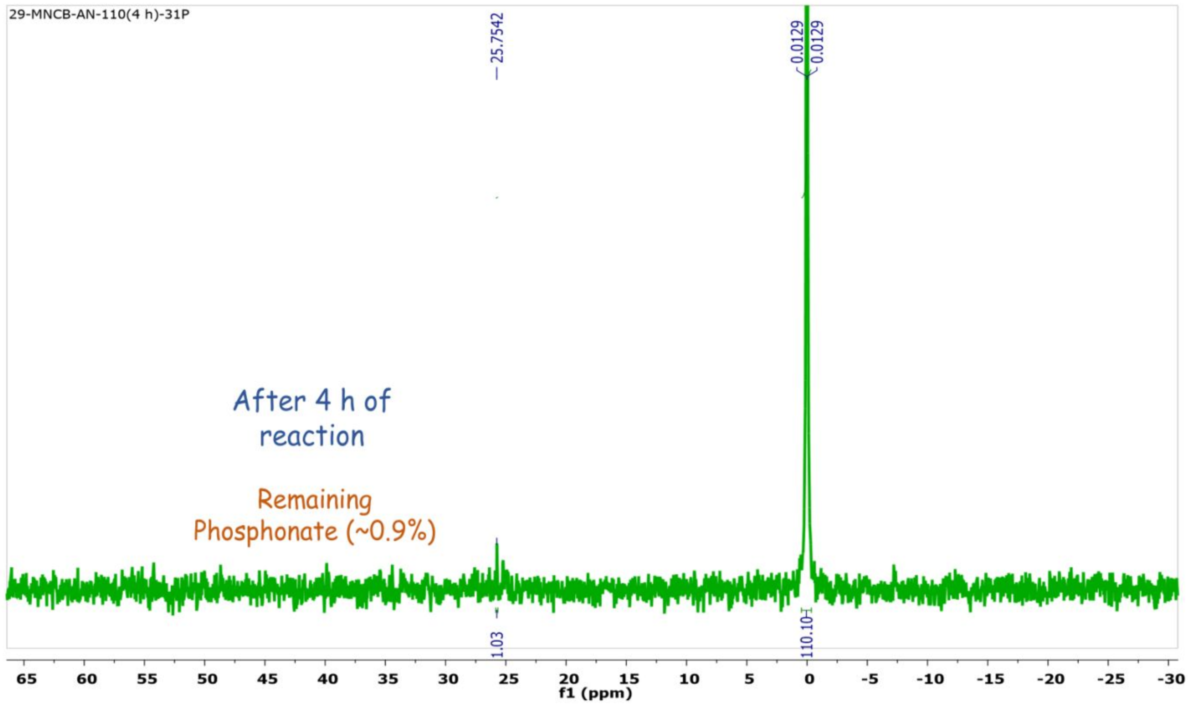

Figure S3: ^{31}P NMR (162 MHz, $CDCl_3$) of reaction mixture after 4 h of reaction progress.

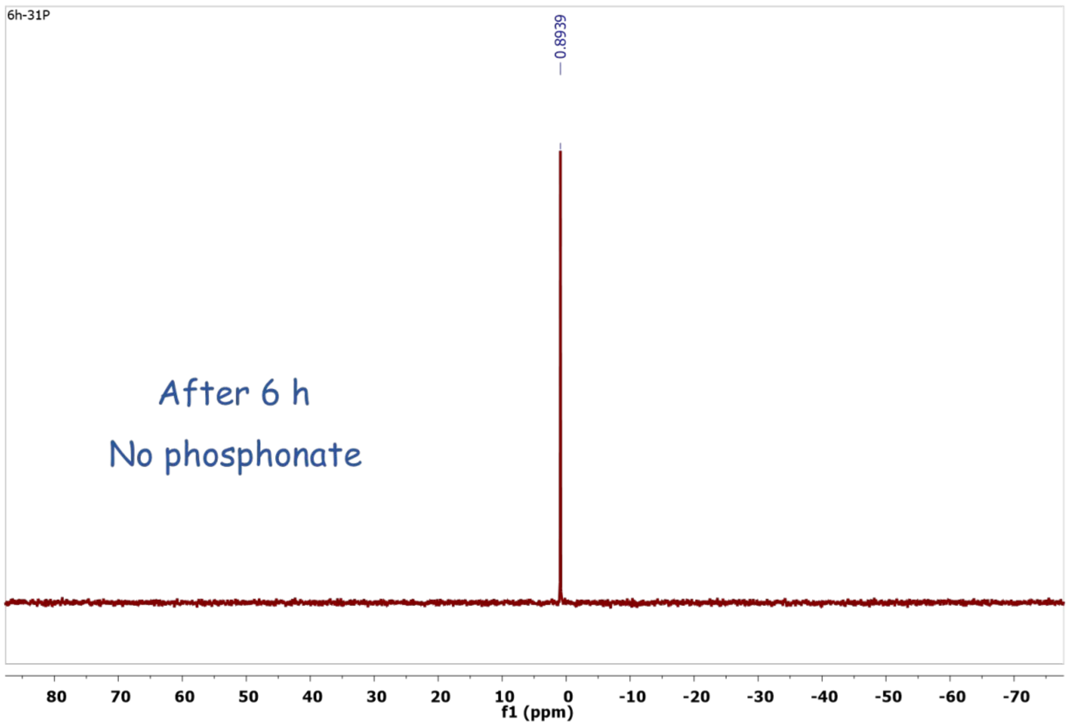

Figure S4: ^{31}P NMR (162 MHz, $CDCl_3$) of reaction mixture after 6 h of reaction progress.

This reaction was optimized further with diverse bases and solvents to find a better way of getting the polymer in maximum yields.

Table S2: Optimization table.

Entry	Base	Solvent	% Yield of Polymer	Unreacted Starting Materials/ Oligomer
1.	^{t}BuOK (6 eq.)	DMF	60	40
2.	NaH (6 eq.)	DMF	50	50
3.	Cs_2CO_3 (6 eq.)	DMF	-	100
4.	^{t}BuOK (6 eq.)	THF	20	80
5.	^{t}BuOK (6 eq.)	DMSO	-	100
6.	^{t}BuOK (6 eq.)	DMAc	-	100
7.	^{t}BuOK (6 eq.)	CH_3CN	-	100
8.	^{t}BuOK (6 eq.)	1,4-Dioxane	20	80
9.	NaH (6 eq.)	THF	20	80

10.	NaH (6 eq.)	DMSO	-	100
11.	NaH (6 eq.)	DMAc	-	100
12.	NaH (6 eq.)	CH_3CN	-	100
13.	NaH (6 eq.)	1,4-Dioxane	-	100
14.	^{t}BuOK (5 eq.)	DMF	50	50
15	^{t}BuOK (6 eq.)	DMF	60	40
16.	^{t}BuOK (7 eq.)	DMF	60	40
17	^{t}BuOK (8 eq.)	DMF	65	35
18.	**^{t}BuOK (9 eq.)**	**DMF**	**75**	**25**
19.	^{t}BuOK (10 eq.)	DMF	75	25
20.	^{t}BuOK (11 eq.)	DMF	75	25

7. Synthesis of linear polymers:

Synthesis of BPV

The linker **BPP** was synthesised by following the reported procedure.[2] Then, using the same general experimental procedure stated above, we synthesized the linear polymer **BPV**.

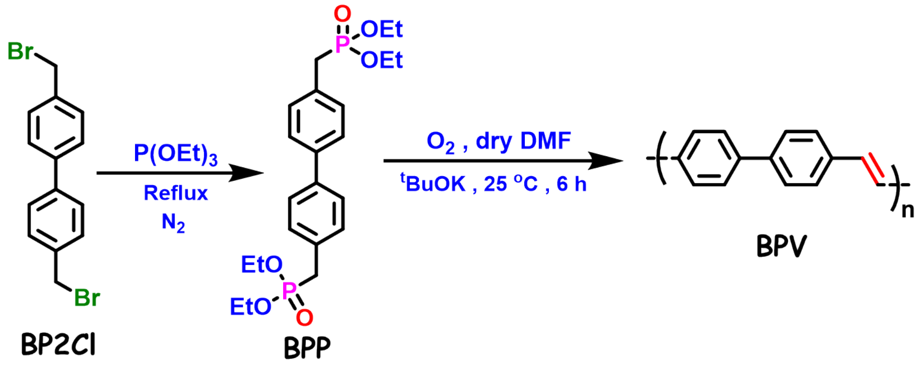

Scheme S2: Synthesis protocol for linear polymer **BPV**.

Synthesis of AMV

The precursor **AMP** was synthesised as described in the literature.[3] Then the linear polymer **AMV** was synthesised following the general experimental procedure.

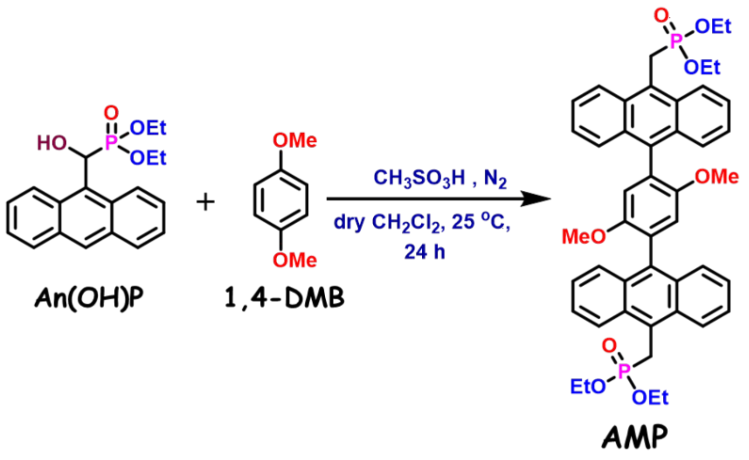

Scheme S3: Synthetic route for **AMP**.

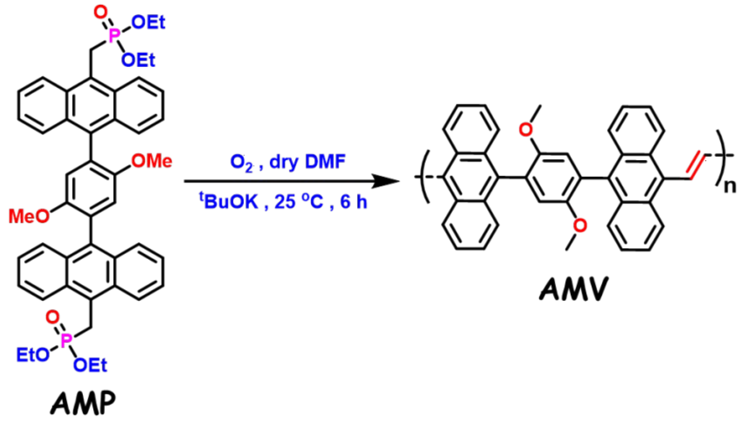

Scheme S4: Synthetic route for **AMV**.

Synthesis of ABV

Synthesis of 2,2′-diheptylbiphenol

0.1 g (0.537 mmol) 2,2′-biphenol was taken in an oven-dry 50 mL RB and dissolved in 10 mL DMSO. 0.075 g (1.34 mmol) KOH was added and stirred for half an hour under N_2, then 0.24 g (1.34 mmol) 1-bromoheptane was added and heated at 120 ℃ (oil bath) for 24 h under N_2 atmosphere. The completion of the reaction was checked by TLC. Then quenched with water, washed with brine, and extracted with dichloromethane (20 mL x 2). The product was purified by column chromatography using fractions of ethyl acetate in petroleum ether (5/90: EtOAc/ Petroleum ether) and appeared as white thick oil.

Yield 78% (0.16 g); ^{1}H NMR (400 MHz, $CDCl_3$) δ 7.29-7.26 (m, 2H), 7.25-7.24 (m, 2H), 6.98-6.92 (m, 4H), 3.89 (t, *J* = 6.6 Hz, 4H), 1.64-1.55 (m, 4H), 1.28-1.22 (m, 16H), 0.87 (t, *J* = 7.0 Hz, 6H). ^{13}C NMR (101 MHz, $CDCl_3$) δ 156.7, 131.7, 128.5, 128.3, 120.1, 112.3, 68.6, 31.9, 29.4, 29.1, 26.0, 22.7, 14.2. HRMS (ESI-TOF) *m/z*: $[M+H]^+$ Calcd for $C_{26}H_{39}O_2$ 383.2950; Found 383.2952

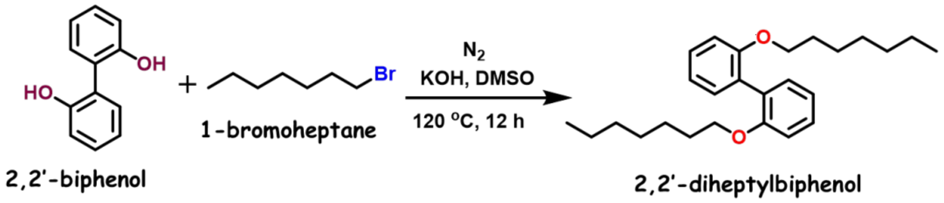

Scheme S5: Synthetic route for 2,2′-diheptylbiphenol.

Synthesis of linker ABP

An oven-dried 50 mL 2-neck RB was cooled under a vacuum Schlenk line. 0.10 g (0.29 mmol) of **An(OH)P** was added to it, followed by the addition of 10 mL of dry CH_2Cl_2. One neck was attached with an N_2 balloon, and the other was closed with a silicon septum. The solution was stirred for 5 min for complete solubilisation. 0.056 mL (0.87 mmol) methanesulfonic acid was injected from the other neck, then a solution of 2,2′-diheptylbiphenol (0.055 g, 0.145 mmol) in 1 mL CH_2Cl_2 was injected into the RB. The reaction was allowed to stir for 24 h at 25 °C. Completion of the reaction was monitored by TLC. The reaction mixture was quenched with water, washed with brine, and extracted with dichloromethane (20 mL x 2). The resulting organic layer was dried over anhydrous sodium sulphate and concentrated. The product was purified by column chromatography using fractions of ethyl acetate in petroleum ether (80/20: EtOAc/petroleum ether). Finally, the product came as white solid.

Yield 60% (0.204 g). M.P. 105-110 °C; FT-IR (ν, cm^{-1}): 3407, 2929, 2854, 1606, 1503, 1469, 1374, 1237, 1021, 952. ^{1}H NMR (400 MHz, $CDCl_3$) δ 8.37 (d, *J* = 8.9 Hz, 4H), 7.82 (d, *J* = 8.8 Hz, 4H), 7.56 – 7.50 (m, 4H), 7.46 (d, *J* = 2.2 Hz, 2H), 7.34-7.30 (m, 4H), 7.24 (d, *J* = 2.2 Hz, 2H), 7.12 (d, *J* = 8.4 Hz, 2H), 4.28 (d, *J* = 22.4 Hz, 4H), 4.04 (t, *J* = 6.6 Hz, 4H), 3.99 – 3.83 (m, 8H), 1.79 – 1.70 (m, 4H), 1.37 – 1.16 (m, 16H), 1.12 (t, *J* = 7.1 Hz, 12H), 0.86 (t, *J* = 7.2

Hz, 6H). ^{13}C NMR (101 MHz, $CDCl_3$) δ 156.1, 137.9, 134.7, 131.2, 130.7, 130.3, 128.2, 127.5, 125.7, 124.9, 124.7, 123.3, 112.0, 68.7, 62.3 (d, *J* = 6.8 Hz), 31.9, 29.6, 29.2, 27.4 (d, *J* = 141.1), 26.1, 22.7, 16.4 (d, *J* = 5.9 Hz), 14.2. ^{31}P NMR (162 MHz, $CDCl_3$) δ 25.9 (s). HRMS (ESI-TOF) *m/z*: $[M+H]^+$ Calcd for $C_{64}H_{77}O_8P_2$ 1035.5094; Found 1035.5093.

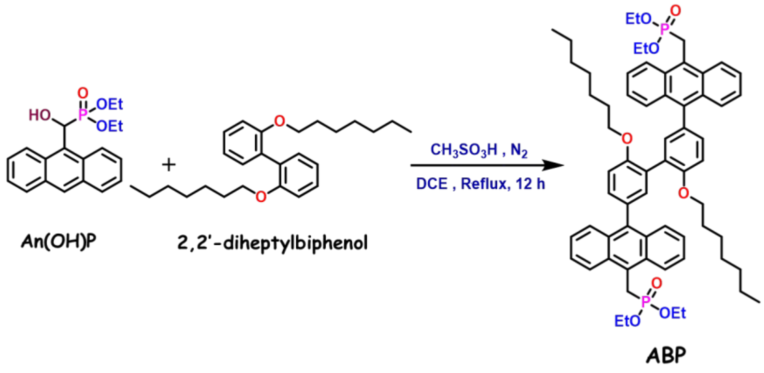

Scheme S6: Synthetic route for **ABP**

Synthesis of ABV polymer

The synthetic procedure for linear polymer **ABV** is the same as mentioned in the above general experimental procedure.

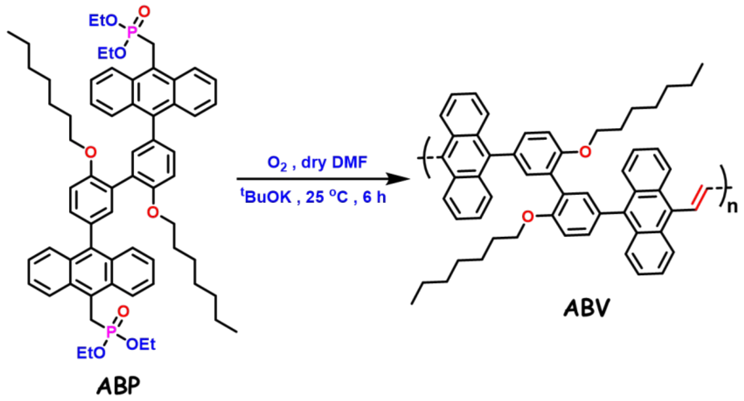

Scheme S7: Synthetic route for **ABV** polymer.

Synthesis of AEV

Synthesis of linker ***PEG3-HQ***

0.1 g (0.908 mmol) hydroquinone was taken in an oven-dry 50 mL RB and dissolved in 10 mL DMSO. 0.127 g (2.27 mmol) KOH was added and stirred for half an hour under N_2, then 0.515g (2.5 eq.) **PEG3-Br** was added and heated at 120 °C (oil bath) for 12 h under a N_2 atmosphere. The completion of the reaction was checked by TLC, then quenched with water, washed with brine, and extracted with dichloromethane (20 mL x 2). The product was purified by column chromatography using fractions of ethyl acetate in petroleum ether (30/70: EtOAc/ Petroleum ether).

Colourless oil, yield 65% (0.24 g). ^{1}H NMR (400 MHz, $CDCl_3$) δ 6.82 (s, 4H), 4.06-4.05 (m, 4H), 3.82 – 3.80 (m, 4H), 3.72 – 3.70 (m, 2H), 3.68 – 3.63 (m, 5H), 3.55 – 3.53 (m, 2H), 3.37 (s, 2H). ^{13}C NMR (101 MHz, $CDCl_3$) δ 153.2, 115.7, 72.6, 72.0, 70.9, 70.7, 69.9, 68.2, 59.2. HRMS (ESI-TOF) *m/z*: $[M+H]^+$ Calcd for $C_{20}H_{35}O_8$ 403.2332; Found 403.2334.

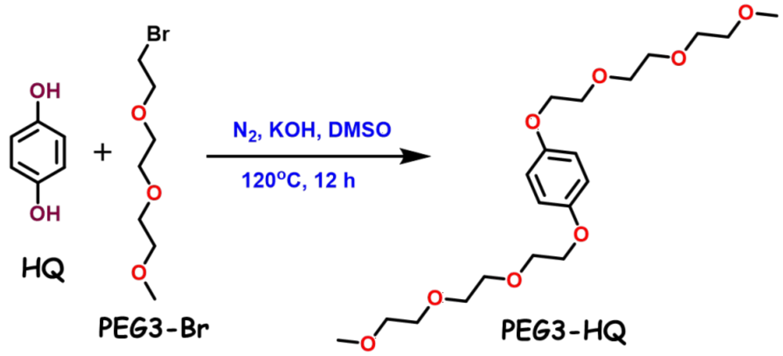

Scheme S8: Synthetic route for **PEG3-HQ**.

*Synthesis of linker **AEP***

An oven-dried 50 mL 2-neck RB was cooled under a vacuum Schlenk line. 0.10 g (0.29 mmol) of **An(OH)P** was added to it, followed by the addition of 10 mL of dry dichloroethane (DCE). One neck was attached with an N_2 balloon, and the other was closed with a silicon septum. The solution was stirred for 5 min for complete solubilisation. 0.056 mL (0.87 mmol) methanesulfonic acid was injected from the other neck, then a solution of PEG3-HQ (0.058 g) in 1 mL DCE was injected into the RB. The reaction was allowed to stir and refluxed (oil bath temperature: 90 °C) for 12 h under a N_2 atmosphere. Completion of the reaction was monitored by TLC. The reaction mixture was quenched with water, washed with brine, and extracted with

dichloromethane (20 mL x 2). The resulting organic layer was dried over anhydrous sodium sulphate and concentrated. The product was purified by column chromatography using fractions of methanol in dichloromethane (DCM) (2/98: MeOH/DCM). Finally, the product came as a whitish solid.

Yield 62% (0.19 g), M.P. 142-147 °C; FT-IR (ν, cm^{-1}): 2892, 1460, 1247, 1022, 961. ^{1}H NMR (400 MHz, $CDCl_3$) δ 8.44 (d, *J* = 8.9 Hz, 4H), 7.90 (d, *J* = 8.7 Hz, 4H), 7.63- (m, 4H), 7.50 (m, 4H), 7.08 (s, 2H), 4.33 (d, *J* = 22.4 Hz, 4H), 4.04 – 3.88 (m, 8H), 3.87 – 3.81 (m, 4H), 3.33 – 3.31 (m, 8H), 3.26 (s, 6H), 3.26 – 3.23 (m, 4H), 3.01-2.91 (m, 4H), 2.85-2.78 (m, 4H), 1.15 (t, *J* = 7.1 Hz, 12H). ^{13}C NMR (101 MHz, $CDCl_3$) δ 151.7, 134.1, 130.4, 128.7, 127.8, 125.8, 125.3, 123.9, 118.1, 71.8, 70.3, 69.6, 69.3, 62.2 (d, *J* = 6.8 Hz), 58.9, 27.5 (d, *J* = 141.2 Hz), 16.4 (d, *J* = 5.9 Hz). ^{31}P NMR (162 MHz, $CDCl_3$) δ 25.8 (s). HRMS (ESI-TOF) *m/z*: $[M+H]^+$ Calcd for $C_{58}H_{73}O_{14}P_2$ 1055.4476; Found 1055.4506.

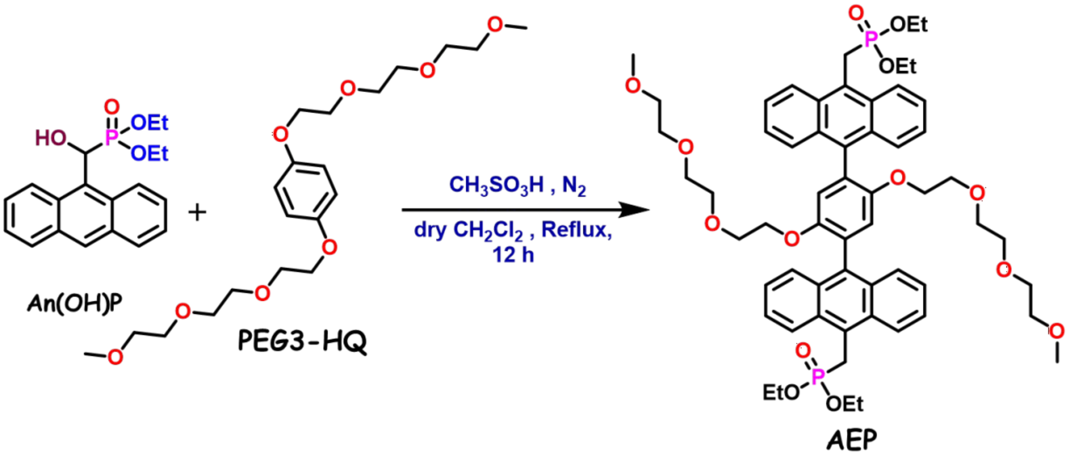

Scheme S9: Synthetic route for **AEP.**

*Synthesis of linear polymer **AEV** and isolated aldehyde **HQ2An2CHO**:*

An oven-dried 50 mL RB was cooled under a vacuum Schlenk line. 0.1 g (0.0947 mmol) of **AEP** was well dissolved in 7 mL dry DMF under an Argon atmosphere at 25 °C, followed by the addition of KO^{t}Bu (0.095g, 0.8523 mmol), and was stirred for 6 h under oxygen (balloon). The completion of the reaction was monitored through thin layer chromatography (TLC). After the completion, there were two spots in TLC; the aldehyde spot was confirmed by 2,4-

dinitrophenylhydrazine (DNP) and separated through column chromatography by using ethyl acetate in hexane (50/50: EtOAc/ Hexane). After removing the aldehyde, we separated the polymer through column chromatography using fractions of methanol in dichloromethane (DCM) (10 /90: MeOH/DCM). Finally, the product came as a brown solid.

AEV: Yield 50 % (0.05 g), decomp. at 135 °C; FT-IR (ν, cm^{-1}): 2921, 2868, 1666, 1502, 1283, 1200, 1103, 1029, 944, 757. ^{1}H NMR (400 MHz, $CDCl_3$) δ 8.84 (br, s), 8.08 (br, s), 7.62 (br, s), 3.99 (br, s), 3.36 (br, s), 1.26 (br, s). ^{13}C NMR (101 MHz, $CDCl_3$) δ 151.9, 134.2, 130.5, 129.6, 127.7, 125.8, 118.3, 71.8, 70.46, 69.4, 59.0, 29.8.

Isolated aldehyde **HQ2An2CHO** (intermediate): It is isolated for validation and characterized by various spectroscopic methods. ^{1}H NMR (in 400 MHz, $CDCl_3$) δ 11.63 (s, 2H), 9.05-9.03 (m, 4H), 7.95-7.92 (m, 4H), 7.72-7.71 (m, 4H), 7.69-7.56 (m,4H), 7.11 (s, 2H), 3.88 (t, J = 4.68, 4H), 3.26-3.25 (m, 4H), 3.24-3.23 (m, 14H), 2.99-2.97 (m, 4H), 2.96-2.80 (m, 4H). ^{13}C NMR (in 101 MHz, $CDCl_3$) δ 193.6, 131.9, 130.1,128.9, 128.0, 126.0, 123.8, 117.2, 71.8, 70.5, 70.3, 69.7, 69.2, 59.0. HRMS (ESI-TOF) *m/z*: $[M+H]^+$ Calcd for $C_{50}H_{51}O_{10}$ 811.3482; Found 811.3479.

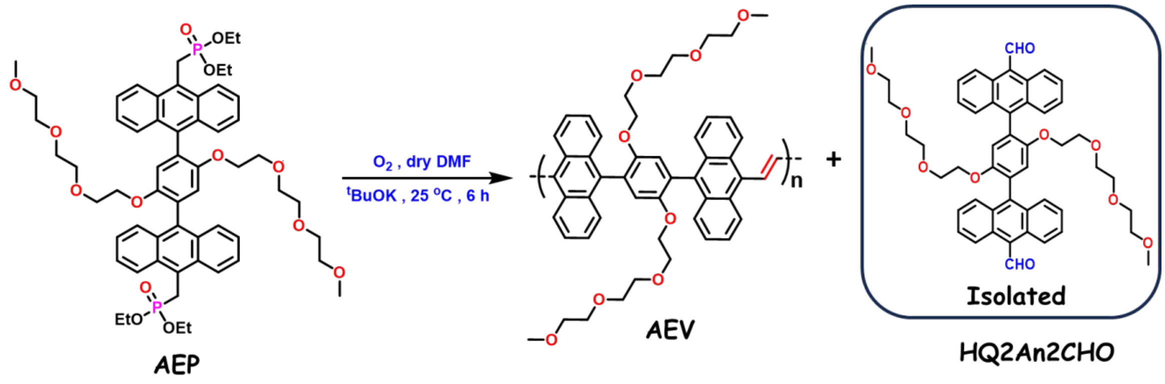

Scheme S10: Synthetic route for **AEV** and **HQ2An2CHO**.

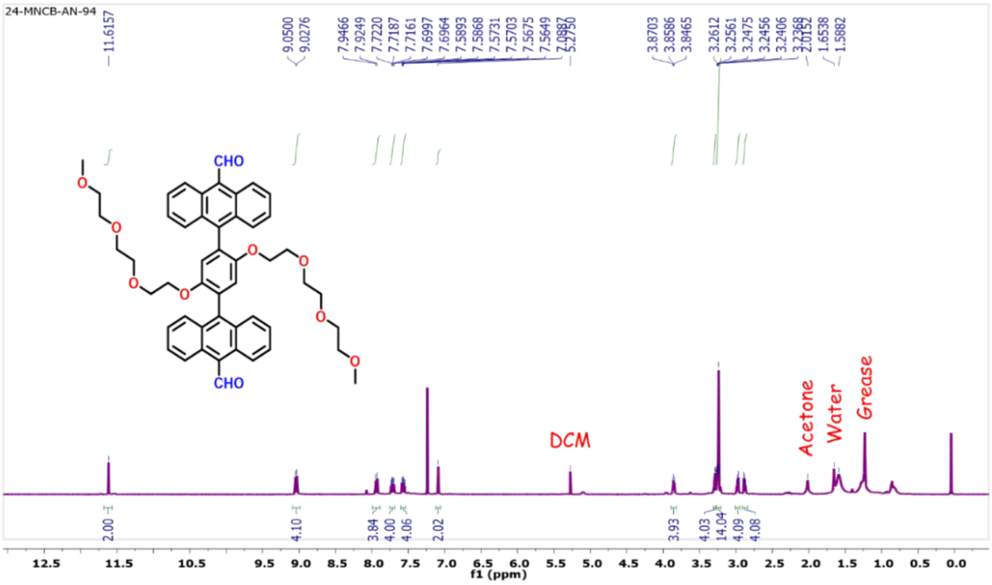

Figure S5: ^{1}H NMR (400 MHz, $CDCl_3$) of isolated aldehyde.

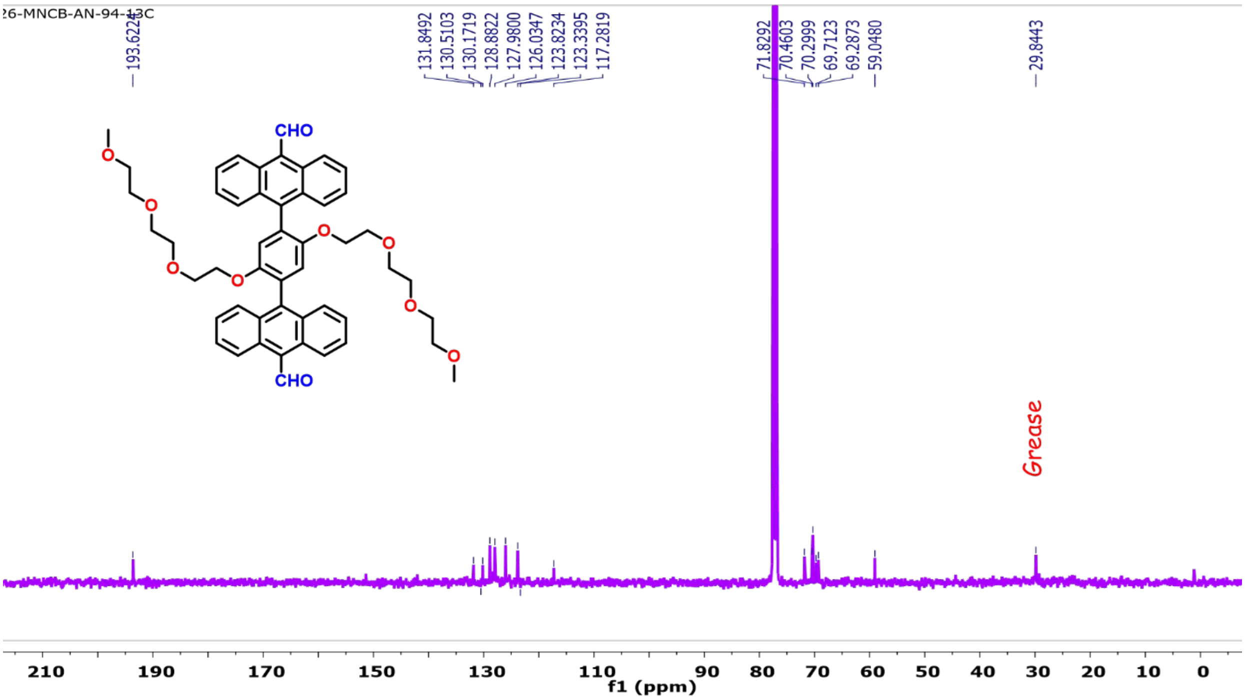

Figure S6: ^{13}C NMR (101 MHz, $CDCl_3$) of isolated aldehyde

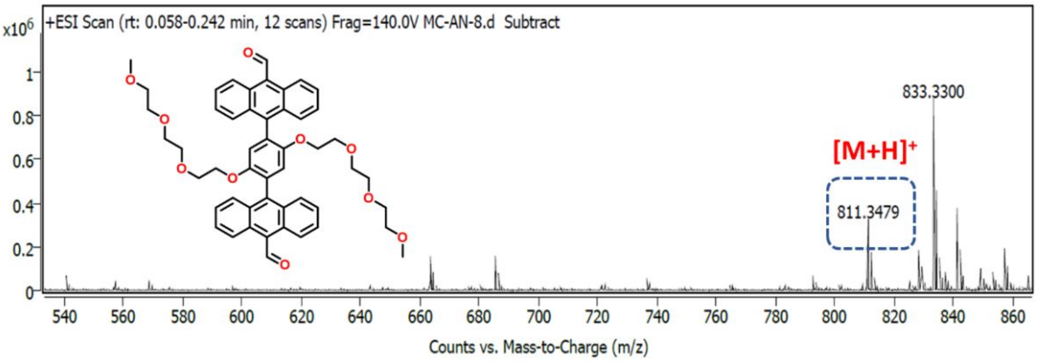

Figure S7: HRMS of isolated aldehyde.

8. Reaction mixture studies using solid and solution state ^{31}P NMR

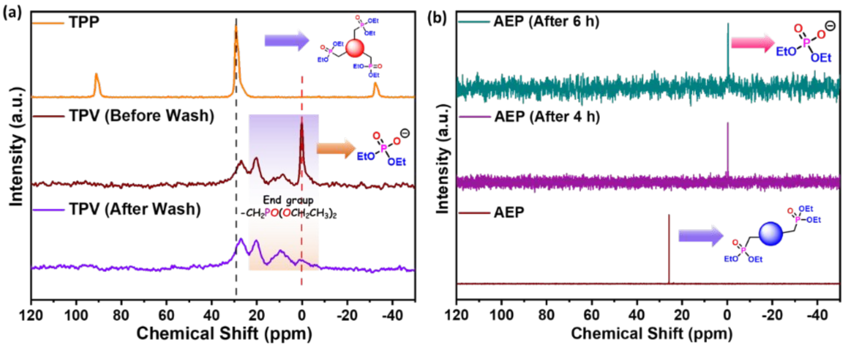

Figure S8: (a) The solid-state ^{31}P NMR (242.9 MHz) spectra of the starting materials **TPP** (after 32 scans, reaction mixture of **TPV** (before wash, after 1223 scans) and **TPV** (after wash, after 2040 scans). (b) The solution state ^{31}P NMR (162 MHz, $CDCl_3$) for the conversion of **AEP** into soluble polymer **AEV**.

9. Synthesis of compounds **TNP**, **TTP**, and **PPP**

Synthesis of TNP

In an oven-dried 50 mL RB was cooled under a vacuum Schlenk line, 0.1 g (0.811 mmol) of 5-methylthiophene-2-carbonitrile (**MTN**) was dissolved in 10 mL of dry carbon tetrachloride (CCl_4). Then 0.173 g (0.974 mmol) *N*-bromosuccinimide (NBS) and 13.33 mg (0.081 mmol) azobisisobutyronitrile (AIBN) were added and refluxed (oil bath temperature:90 ^{o}C) for 6 h under N_2. TLC checked the completion of the reaction. Without further purification, the 0.1 g (0.494 mmol) compound **BrMTN** was taken in an oven-dried 50 mL RB, then dissolved in 0.424 mL (2.47 mmol) triethyl phosphite. The reaction was heated for 6 h (oil bath temperature: 140 ^{o}C) under a N_2 atmosphere. Reaction completion was checked through TLC, and the

reaction mixture was quenched with water, washed with brine, and extracted with dichloromethane (20 mL x 2). The resulting organic layer was dried over anhydrous sodium sulphate and concentrated. The product was purified by column chromatography using fractions of ethyl acetate in petroleum ether (25/75: EtOAc/Hexane).

Brownish liquid, yield 78% (0.10 g). ^{1}H NMR (in 400 MHz, $CDCl_3$) δ 7.49-7.48 (m,1H), 7.00-6.98 (m,1H), 4.13- 4.06 (m, 4H), 3.36 (d, J = 20.84, 2H), 1.31-1.28 (m, 6H). ^{13}C NMR (101 MHz, $CDCl_3$) δ ppm: 141.4 (d, J = 10.3 Hz), 137.6, 127.9 (d, J = 8.3 Hz), 114.1, 108.9, 62.8 (d, J = 6.7 Hz), 28.5 (d, J = 144 Hz), 16.4 (d, J = 5.9 Hz). ^{31}P NMR (162 MHz, $CDCl_3$) δ 22.0 (s). HRMS (ESI-TOF) *m/z*: $[M+H]^+$ Calcd for $C_{10}H_{15}NO_3PS$ 260.0510; Found 260.0511.

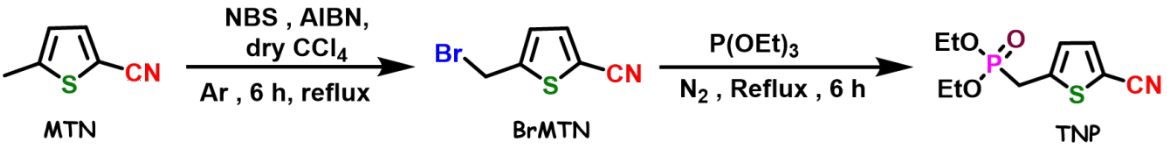

Scheme S11: Synthetic route for **TNP**.

Synthesis of linker TTP

An oven-dried 2-neck 50 mL RB was cooled under a vacuum Schlenk line, and 0.1 g (0.385 mmol) **TNP** was dissolved in 10 mL of dry chloroform ($CHCl_3$). One neck was attached with an N_2 balloon, and the other was connected with a dropping funnel through which 0.102 mL (1.157 mmol) of CF_3SO_3H was added dropwise into the RB at 0 °C. The mixture was stirred at 25 °C for 24 h, then poured into ice water and neutralized with ammonium hydroxide. The completion of the reaction was checked by TLC. Then the reaction mixture was washed with brine and extracted with dichloromethane (20 mL x 2). The resulting organic layer was dried over anhydrous sodium sulphate and concentrated. The product was purified by column chromatography using fractions of methanol in DCM (2/98: MeOH/DCM). Finally, the product came as a white solid. This compound was reported in the literature.[4]

Yield 65% (0.195 g), M.P. 162-167 °C; FT-IR (ν, cm^{-1}): 3376, 3178, 1658, 1601, 1459, 1387, 1223, 1020, 956. ^{1}H NMR (in 400 MHz, $CDCl_3$) δ 7.41-7.39 (m, 3H), 6.96-6.95 (m, 3H), 4.11-4.05 (m, 12H), 3.34 (d, J = 21.2 Hz, 6H), 1.29 (t, J = 7.08 Hz, 18H). ^{13}C NMR (101 MHz, $CDCl_3$) δ 167.5, 140.7, 140.6, 132.1, 129.2 (d, J = 7.9 Hz), 62.9 (d, J = 6.7 Hz), 29.1 (d, J =

143.2 Hz), 16.7 (d, J = 5.7 Hz). ^{31}P NMR (162 MHz, CDC13) δ 23.9 (s). MS (ESI) *m/z*: $[M+H]^+$ Calcd for $C_{30}H_{43}N_3O_9P_3S_3$ 778.14; Found 778.15.

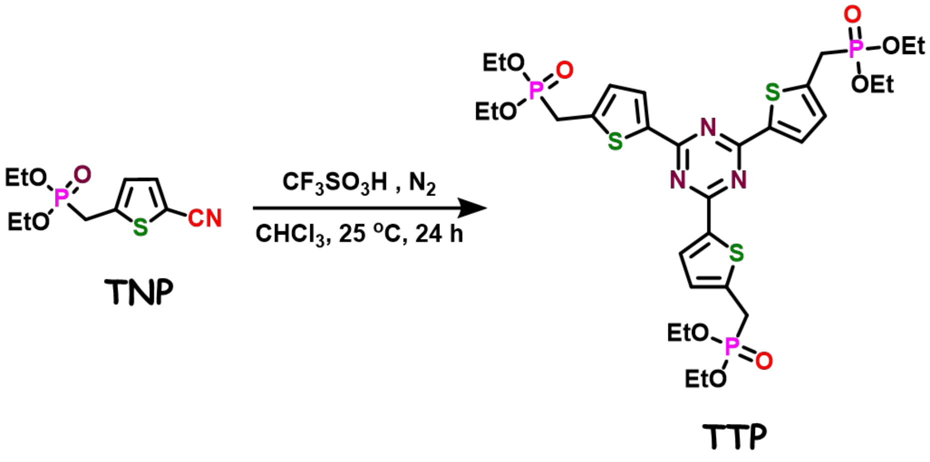

Scheme S12: Synthetic route for **TTP**

Synthesis of linker PPP

0.1 g (0.287 mmol) 4,4”-Dimethyl-5’-(p-tolyl)-1,1’:3’,1”-terphenyl was taken in an oven-dry 50 mL RB and dissolved in 7 mL dry CCl_4. 0.153g (8.608 mmol) N-bromo succinimide followed by 4.71 mg (0.0286 mmol) AIBN, were added and refluxed (oil bath temperature: 90 ^{o}C) for 12 h under an Argon atmosphere. The completion of the reaction was checked by TLC. Without doing further purification, the 0.1 g (0017 mmol) compound **TPP3Br** was taken in an oven-dried 50 mL RB and dissolved in 0.437 mL (2.55 mmol) triethyl phosphite. The reaction was heated (oil bath temperature: 140 ^{o}C) for 12 h under a N_2 atmosphere. Reaction completion was checked through TLC, and the excess triethyl phosphite was removed by distillation. The reaction mixture was quenched with water, washed with brine, and extracted with dichloromethane (20 mL x 2). The product was purified by column chromatography using fractions of Methanol in DCM (2/98: MeOH/DCM). Finally, the product came as yellowish oil.

Yield 55% (0.071 g), FT-IR (ν, cm^{-1}): 2981, 2919, 1449, 1392, 1247, 1022, 961. ^{1}H NMR (in 400 MHz, $CDCl_3$) δ 7.75 (s, 3H), 7.65-7.63 (m, 6H), 7.42-7.39 (m, 6H), 4.09-4.02 (m, 12H), 3.21(d, J = 21.6 Hz, 6H), 1.27 (t, J = 7.08 Hz, 18H). ^{13}C NMR (101 MHz, $CDCl_3$) δ 142.0,

139.8, 131.2, 130.4, 127.6, 125.0, 62.4 (d, *J* = 6.7 Hz), 33.6 (d, *J* = 138.66 Hz), 16.6 (d, *J* = 5.9 Hz). ^{31}P NMR (162 MHz, CDCl3) δ 26.2 (s). HRMS (ESI-TOF) *m/z*: $[M + H]^+$ Calcd for $C_{39}H_{52}O_9P_3$ 757.2824; Found 757.2830.

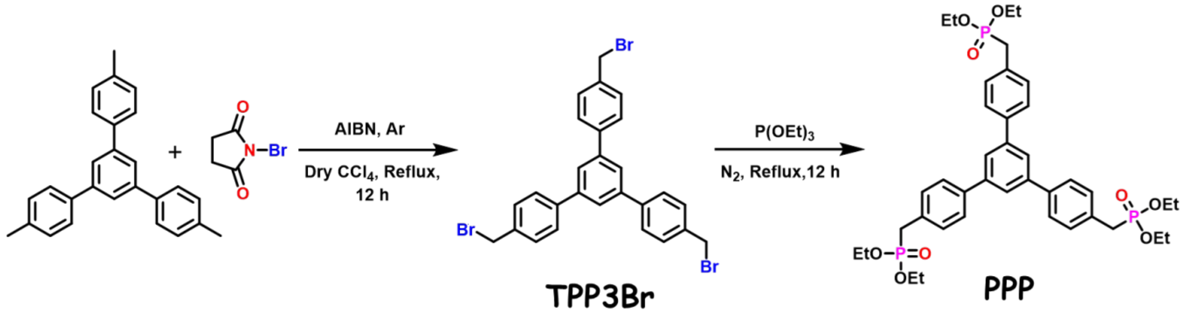

Scheme S13: Synthetic route for **PPP**

10. Synthesis of TTV and PPV polymer

The polymer **TTV** and **PPV** were synthesised following the same general experimental procedure, as stated above.

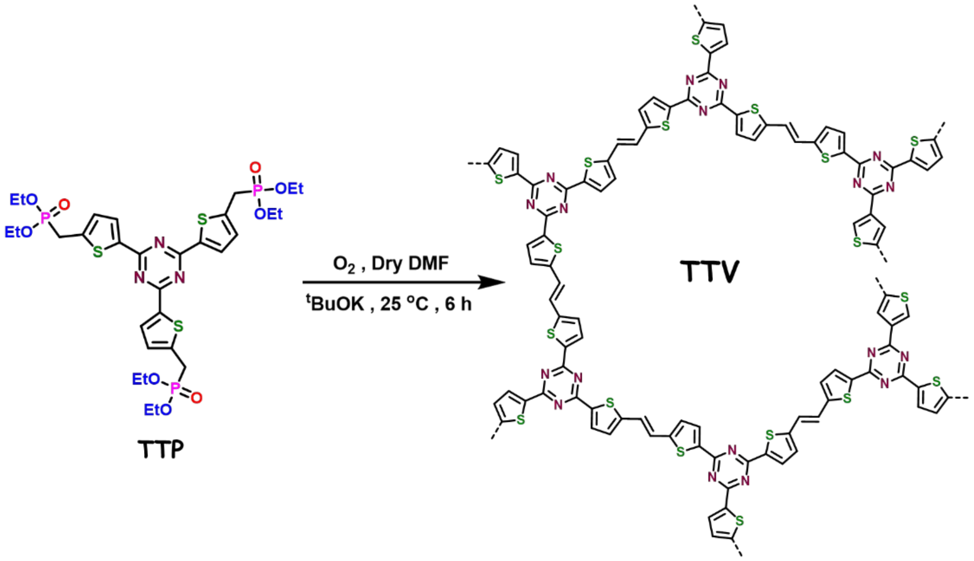

Scheme S14: Synthetic protocol for cyclic polymer **TTV**.

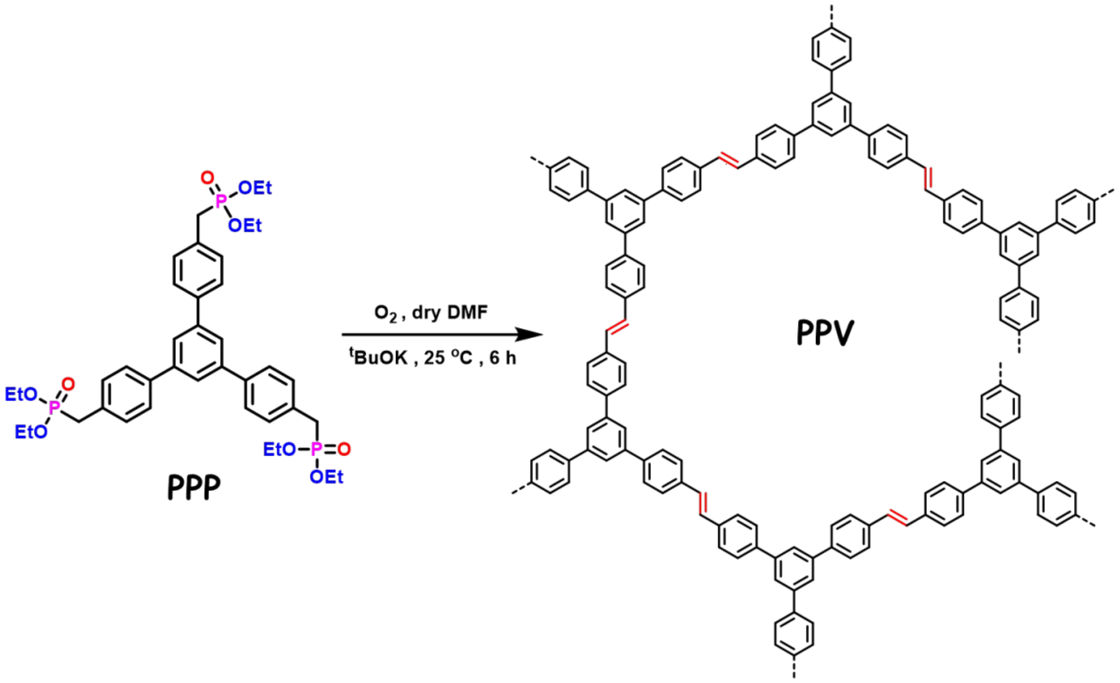

Scheme S15: Synthesis protocol for cyclic polymer **PPV**.

11. Characterisation of TPV:

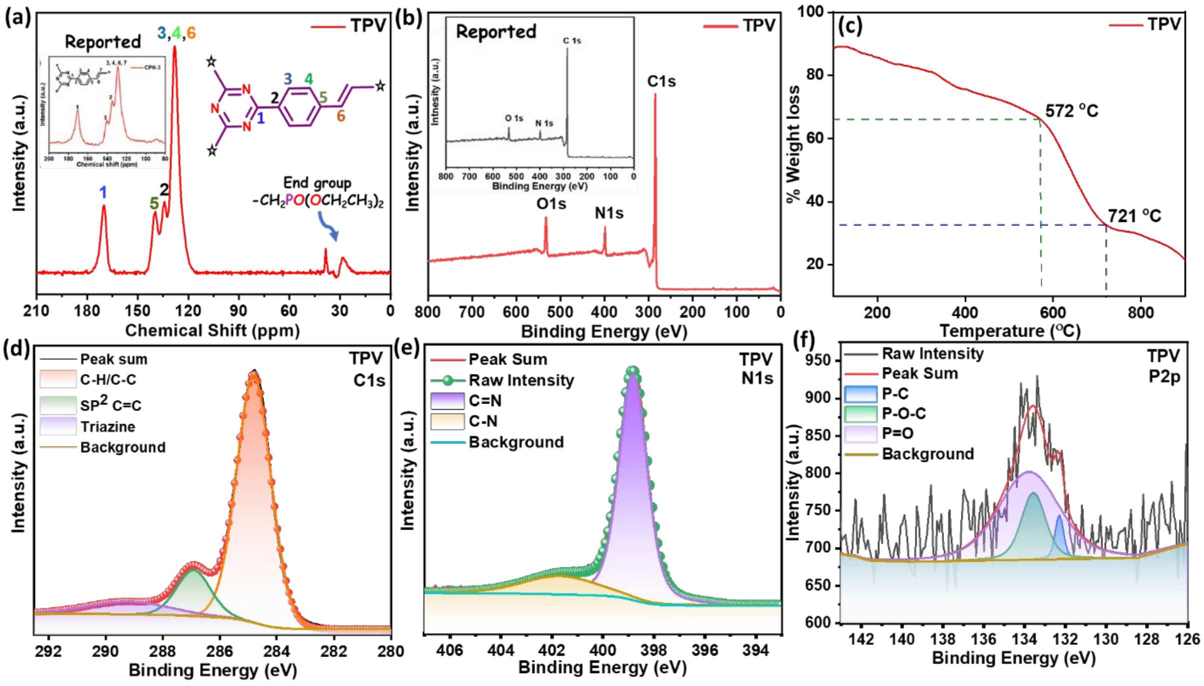

Figure S9: (a) Solid-state ^{13}C NMR (b) XPS spectra (inside reported one which matches with our experimental outcomes),[5] (c) TGA; Deconvoluted and high-resolution XPS spectra of **TPV:** (d) C1s, (e) N1s, (f) P2p.

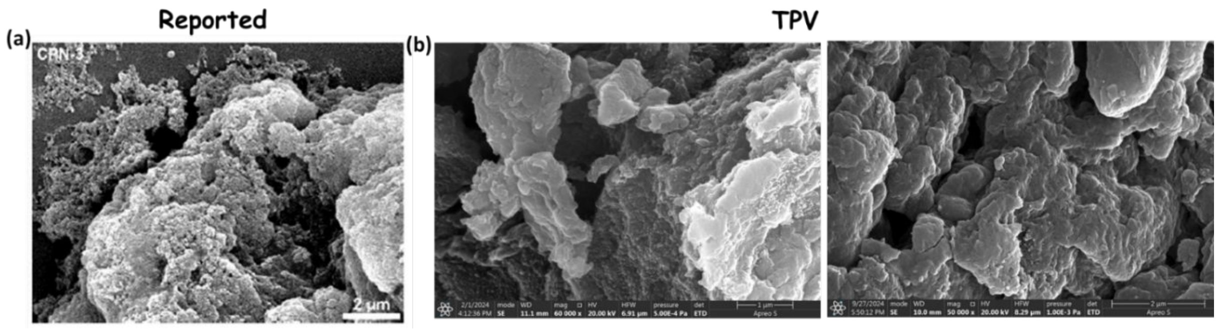

Figure S10: SEM images of (a) reported[5] and (b) as-synthesised polymer.

12. Synthesis of linker TTP and TTA

The synthesis of linker TTP has been presented before.

Synthesis of linker TTA

0.1 g (0.729 mmol) 5-formyl thiophene-2-carbonitrile was taken in an oven-dried 50 mL RB and dissolved in 5 mL chloroform ($CHCl_3$). 0.194 mL (0.729 mmol) of CF_3SO_3H was added dropwise through a dropping funnel at 0 °C under N_2 atmosphere. The mixture was stirred at 25 °C for 24 h, then poured into ice-water and neutralised with ammonium hydroxide. The precipitate was filtered out and then washed with water and acetone several times to get it pure. Finally, the product appeared as a white solid.

Yield 60% (0.18 g), M.P. 188-193 °C. ^{1}H NMR (in 400 MHz, DMSO-*d*$_6$) δ 9.95 (s, 3H), 8.00 (d, *J* = 3.92 Hz, 3H), 7.86 (d, *J* = 3.92 Hz, 3H). ^{13}C NMR (101 MHz, DMSO-*d*$_6$) δ 185.6, 162.5, 148.5, 146.0, 138.1, 129.8. This compound was reported in the literature.[6]

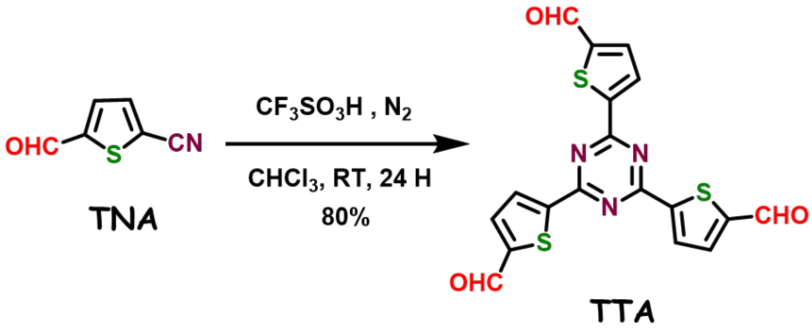

Scheme S16: Synthesis protocol for **TTA** linker.

13. Synthesis of TTV through novel HWE strategy

An oven-dried 50 mL RB was cooled under a vacuum Schlenk line. 0.1g (0.1286 mmol) of **TTP** was well dissolved in 7 mL dry DMF under an Argon atmosphere at 25 °C, followed by the addition of KO^{t}Bu (0.115 g, 1.0288 mmol) and (0.053 g, 0.1286 mmol) **TTA,** then stirred for 12 h at 25 ℃. The completion of the reaction was monitored through thin layer chromatography (TLC). After the completion, the reaction was quenched with ice-cold water and insoluble precipitates were formed, which were filtered and washed with DCM, methanol, THF and acetone (50 mL × 3 times) to remove any unreacted monomer, oligomer and any other impurities. The residual solid was dried under vacuum for 24 h at 60 ℃ to deliver a yellow solid.

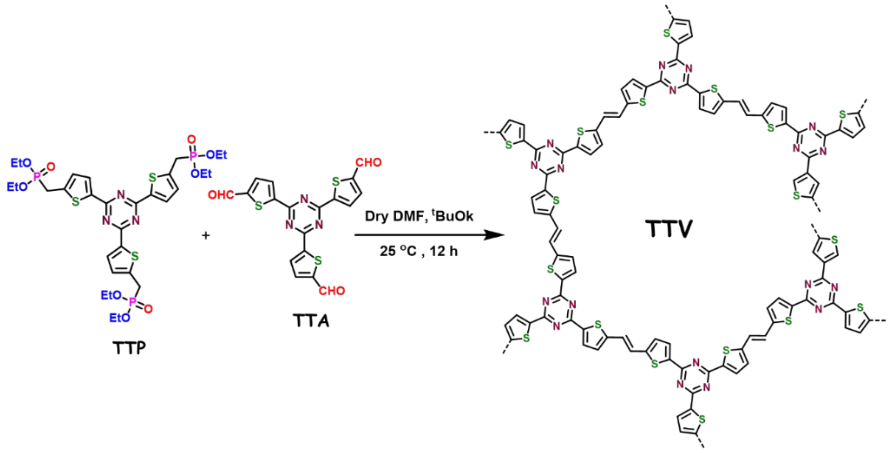

Scheme S17: Synthesis protocol for **TTV** polymer using aldehyde.

14. Characterisation of TTV

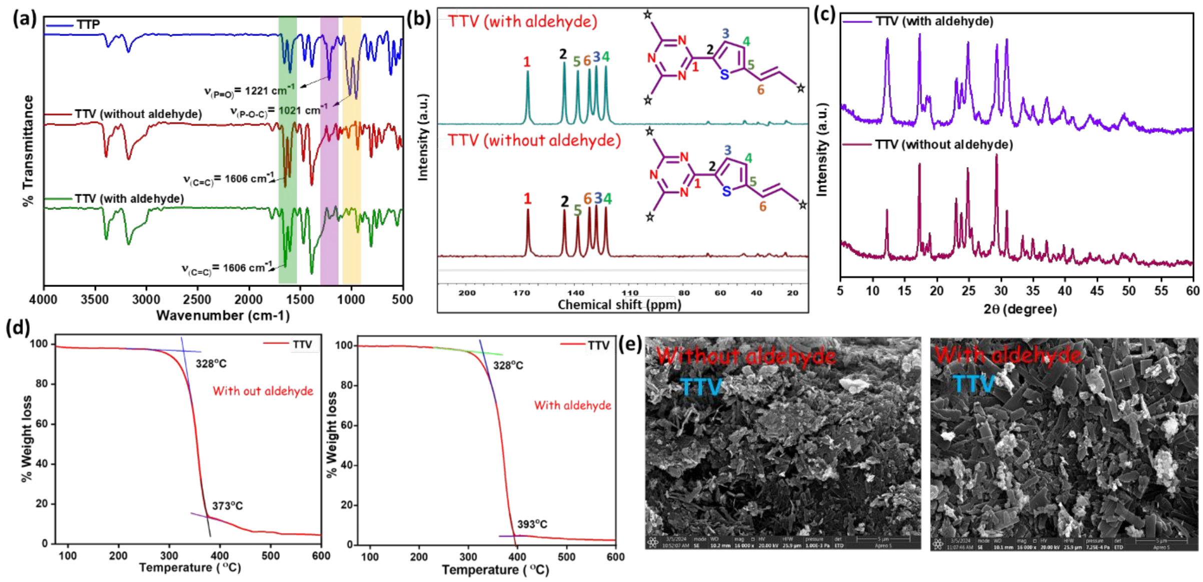

Figure S11. Comparison of the polymers (**TTV**) synthesized from phosphonate by using aldehyde precursor and oxidative dephosphorylation method. (a) FT-IR; (b) ss-^{13}C NMR; (c) PXRD; (d) TGA, and (e) SEM images.

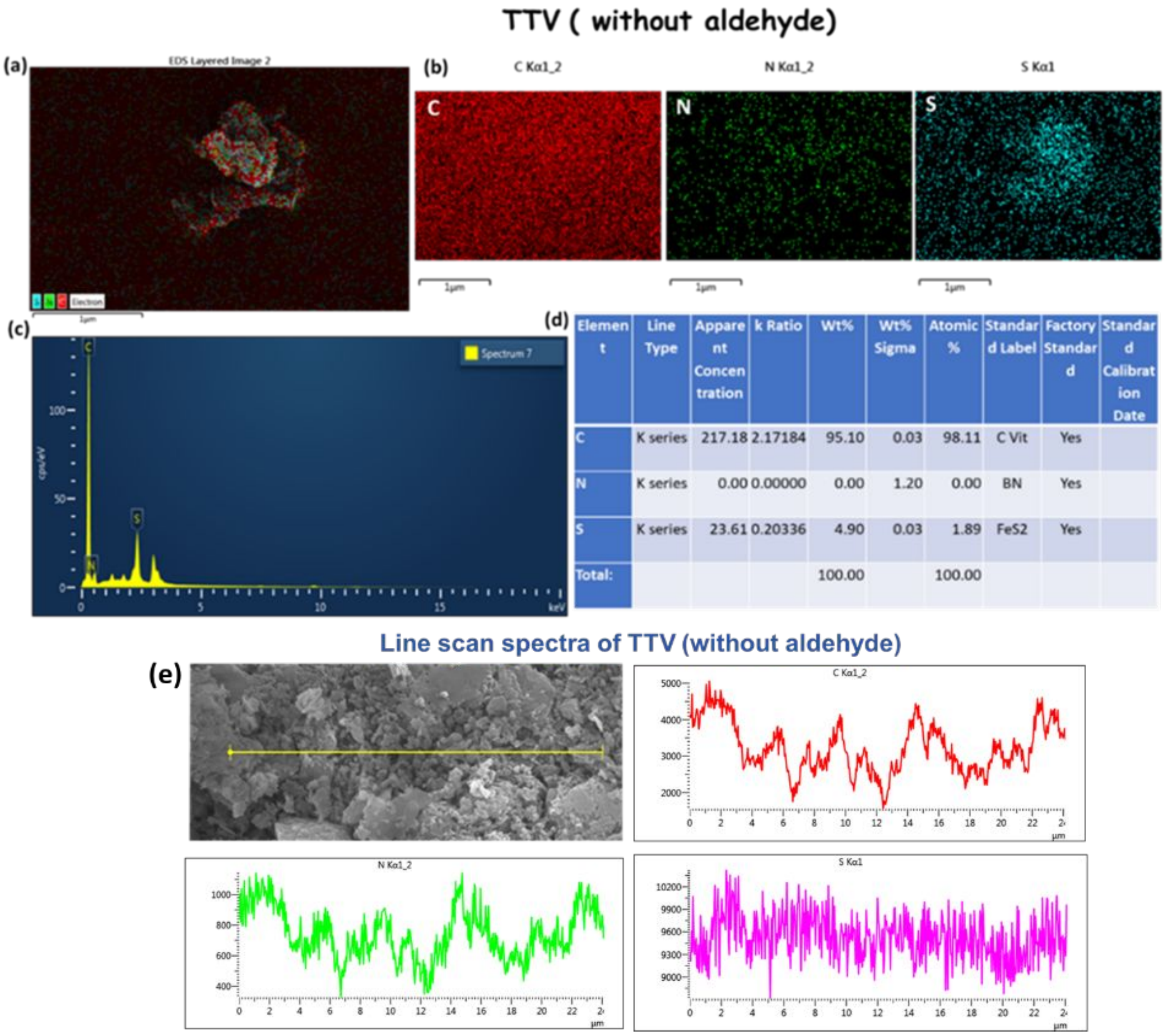

Element	Line Type	Apparent Concentration	k Ratio	Wt%	Wt% Sigma	Atomic %	Standard Label	Factory Standard	Standard Calibration Date
C	K series	217.18	2.17184	95.10	0.03	98.11	C Vit	Yes	
N	K series	0.00	0.00000	0.00	1.20	0.00	BN	Yes	
S	K series	23.61	0.20336	4.90	0.03	1.89	FeS2	Yes	
Total:				100.00		100.00			

Figure S12: (a) Elemental mapping for **TTV** polymer (synthesized directly from phosphonate, without using aldehyde), (b) Individual colour map of carbon, nitrogen, sulphur, (c) EDX plot and (d) % content of elements. (e) Line scan spectra of the synthesized polymer

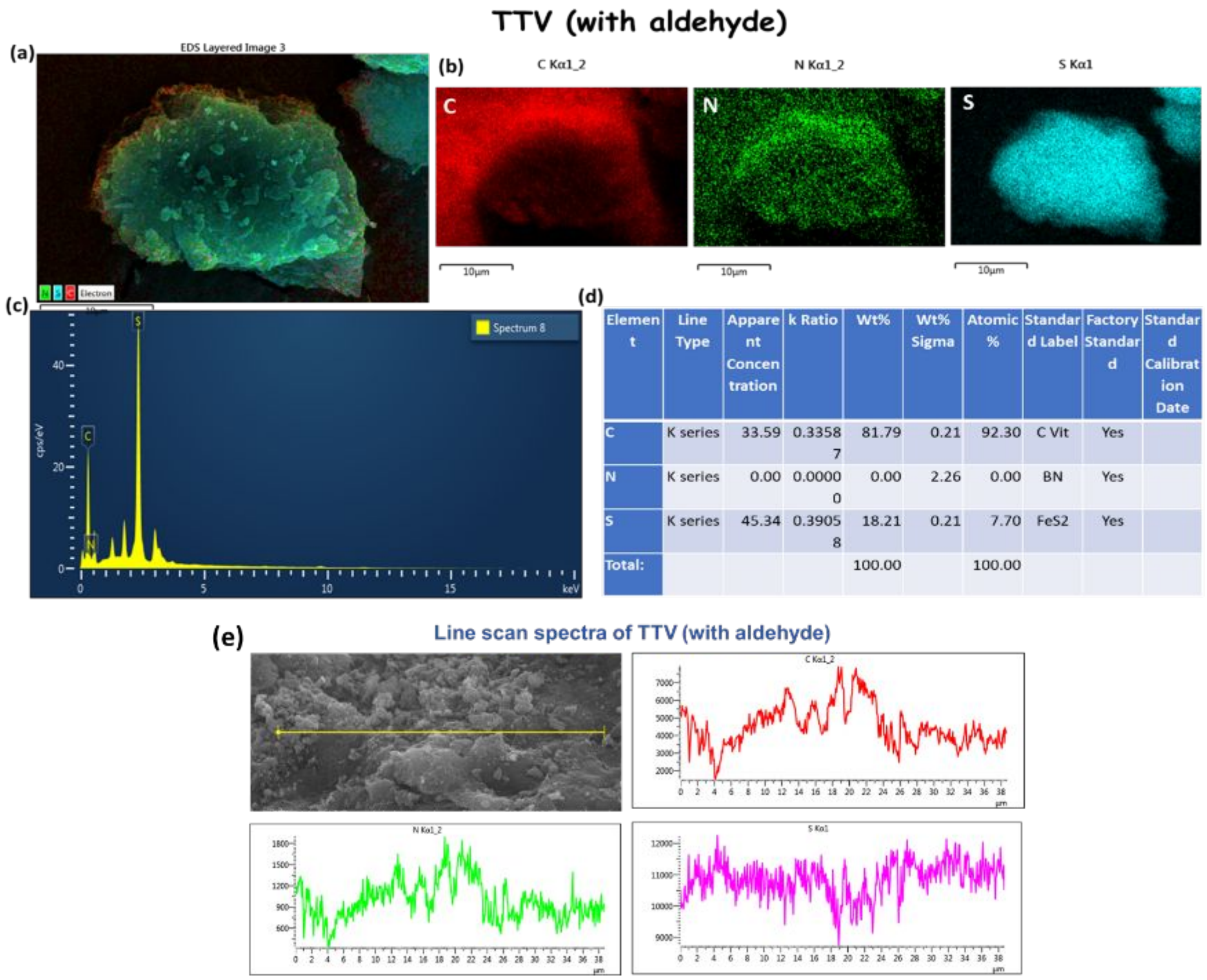

Element	Line Type	Apparent Concentration	k Ratio	Wt%	Wt% Sigma	Atomic %	Standard Label	Factory Standard	Standard Calibration Date
C	K series	33.59	0.33587	81.79	0.21	92.30	C Vit	Yes	
N	K series	0.00	0.00000	0.00	2.26	0.00	BN	Yes	
S	K series	45.34	0.39058	18.21	0.21	7.70	FeS2	Yes	
Total:				100.00		100.00			

Figure S13: (a) Elemental mapping for **TTV** polymer (synthesized using aldehyde), (b) Individual colour map of carbon, nitrogen, sulphur, (c) EDX plot, and (d) % content of elements. (e) Line scan spectra of the synthesized polymer.

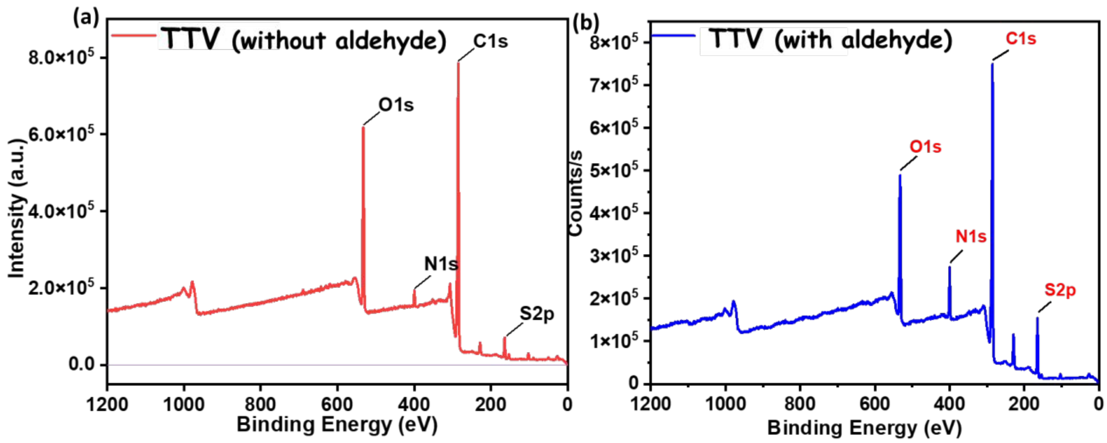

Figure S14: XPS spectra of (a) **TTV** (without aldehyde), (b) **TTV** (with aldehyde).

15. All characterization of PPV

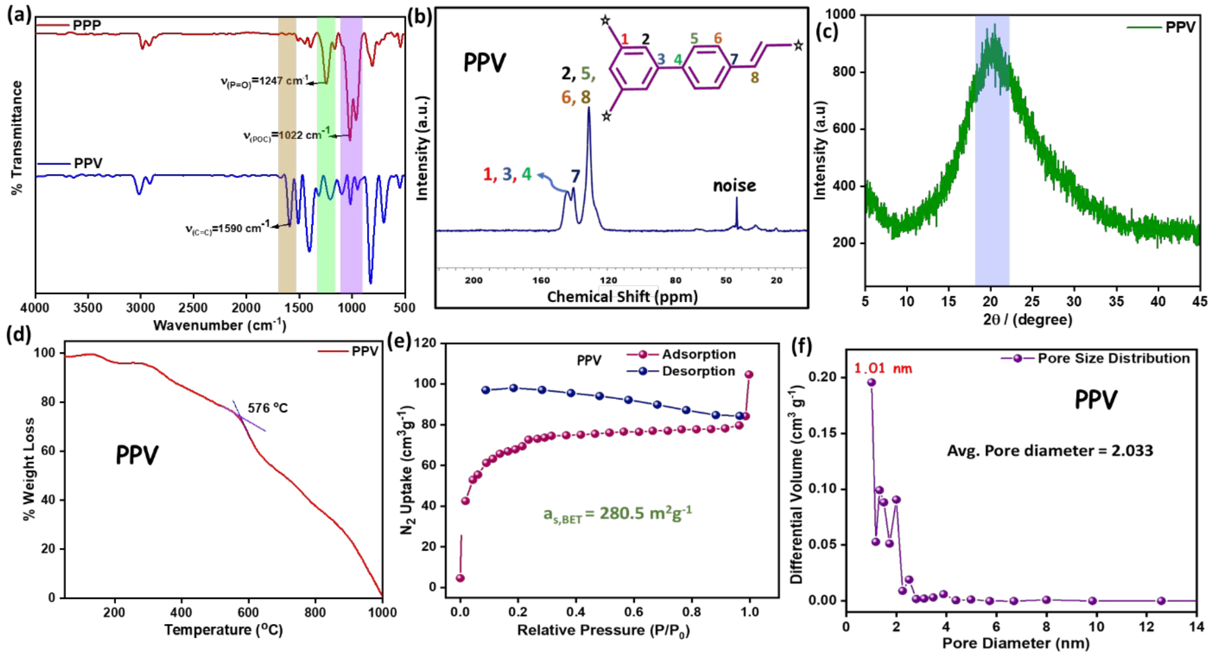

Figure S15: (a) FT-IR spectra, (b) Solid-state ^{13}C NMR spectra, (c) PXRD pattern, (d) TGA diagram, (e) BET adsorption isotherm, (f) Pore size distribution, for **PPV** polymer.

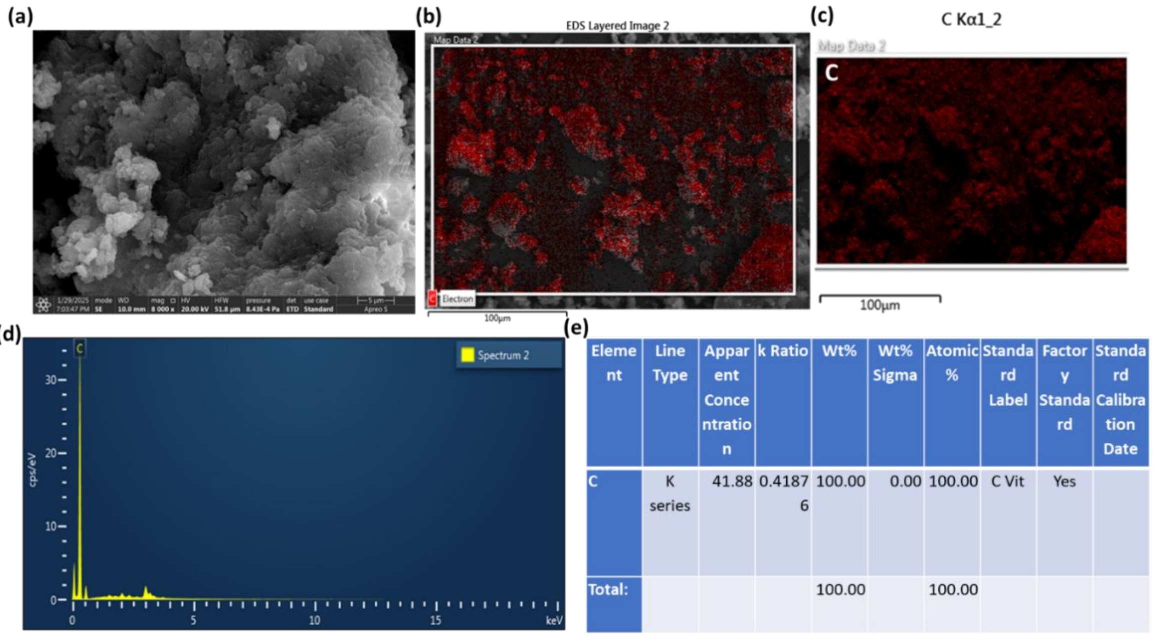

Element	Line Type	Apparent Concentration	k Ratio	Wt%	Wt% Sigma	Atomic %	Standard Label	Factory Standard	Standard Calibration Date
C	K series	41.88	0.41876	100.00	0.00	100.00	C Vit	Yes	
Total:				100.00		100.00			

Figure S16: (a) SEM images, (b) elemental mapping, (c) individual colour map of carbon, (d) EDX plot, and (e) % content of elements, for **PPV** polymer.

16. Characterization of 1D-polymers [BPV, AMV, ABV, and AEV]

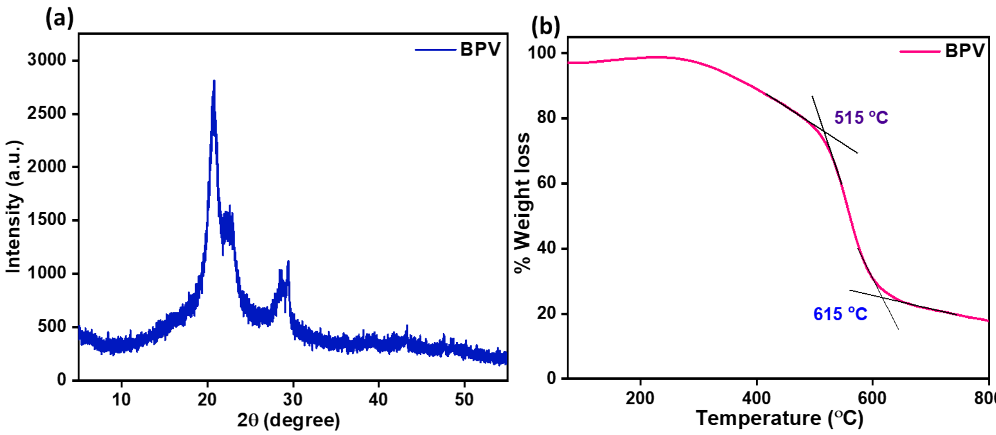

Figure S17: (a) PXRD pattern, and (b) TGA diagram for **BPV** polymer.

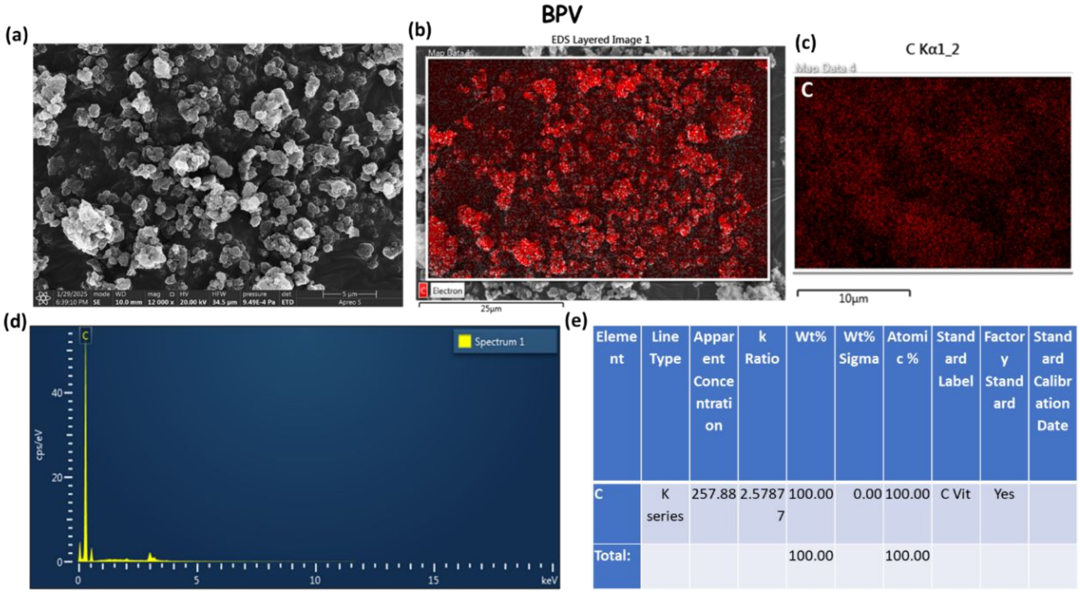

Element	Line Type	Apparent Concentration	k Ratio	Wt%	Wt% Sigma	Atomic %	Standard Label	Factory Standard	Standard Calibration Date
C	K series	257.88	2.57877	100.00	0.00	100.00	C Vit	Yes	
Total:				100.00		100.00			

Figure S18: (a) SEM images, (b) elemental mapping, (c) individual colour map of carbon, (d) EDX plot, and (e) % content of elements, for **BPV** polymer.

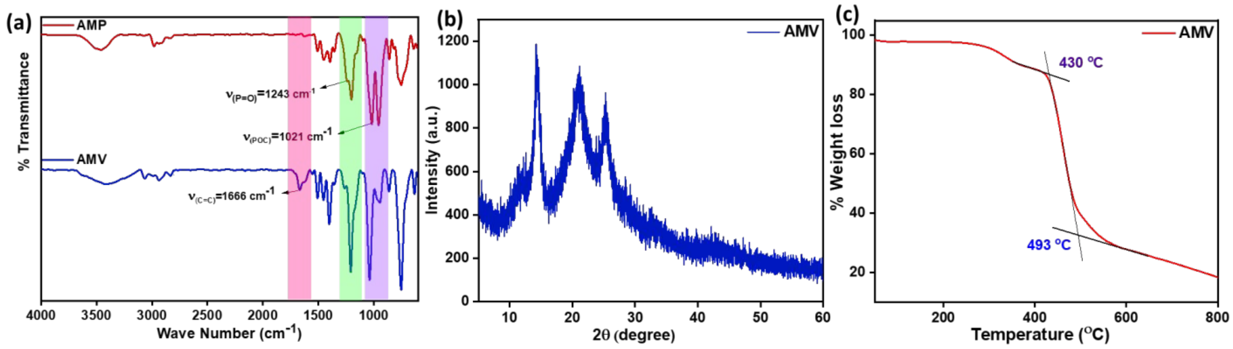

Figure S19: (a) IR spectra, (b) PXRD pattern, and (c) TGA diagram for **AMV** polymer.

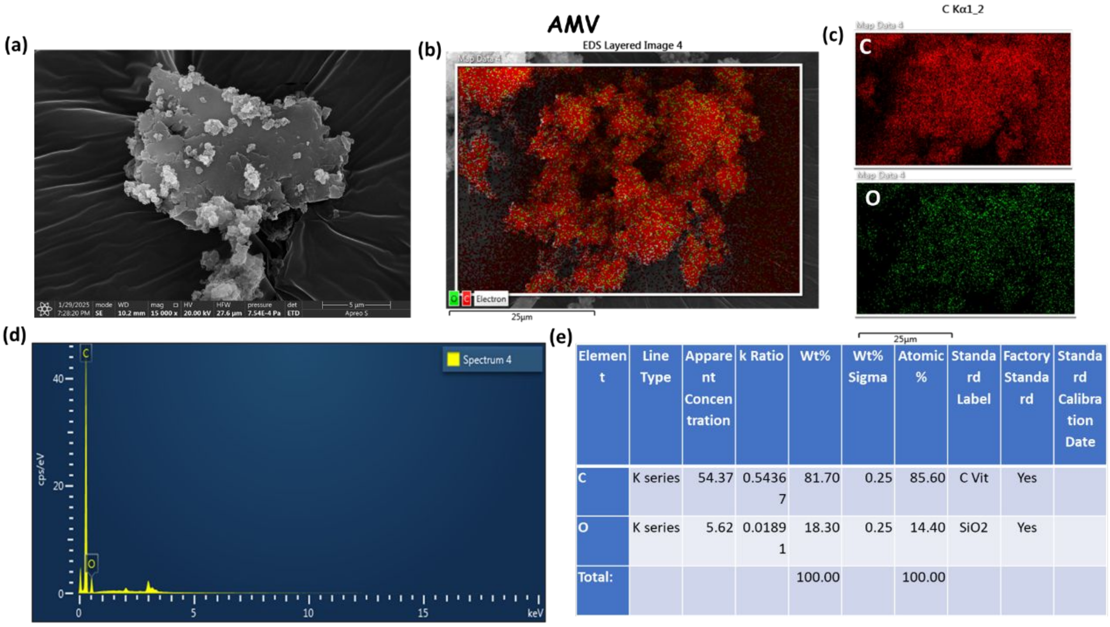

Element	Line Type	Apparent Concentration	k Ratio	Wt%	Wt% Sigma	Atomic %	Standard Label	Factory Standard	Standard Calibration Date
C	K series	54.37	0.54367	81.70	0.25	85.60	C Vit	Yes	
O	K series	5.62	0.01891	18.30	0.25	14.40	SiO2	Yes	
Total:				100.00		100.00			

Figure S20: (a) SEM images, (b) elemental mapping, (c) individual colour map of carbon, (d) EDX plot, and (e) % content of elements, for **AMV** polymer.

Characterization of ABV

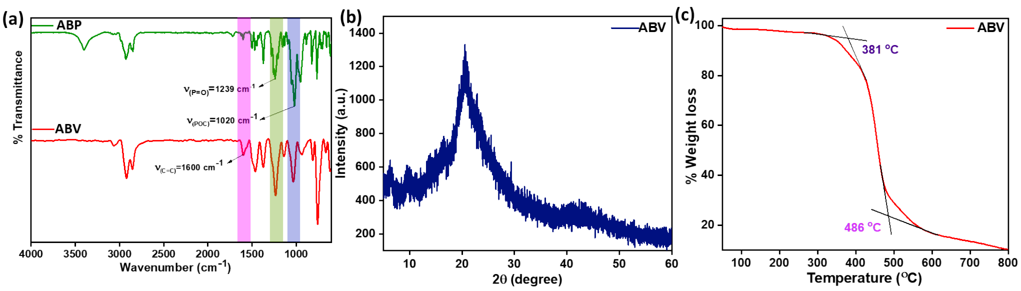

Figure S21: (a) IR spectra, (b) PXRD pattern, and (c) TGA diagram for **ABV** polymer.

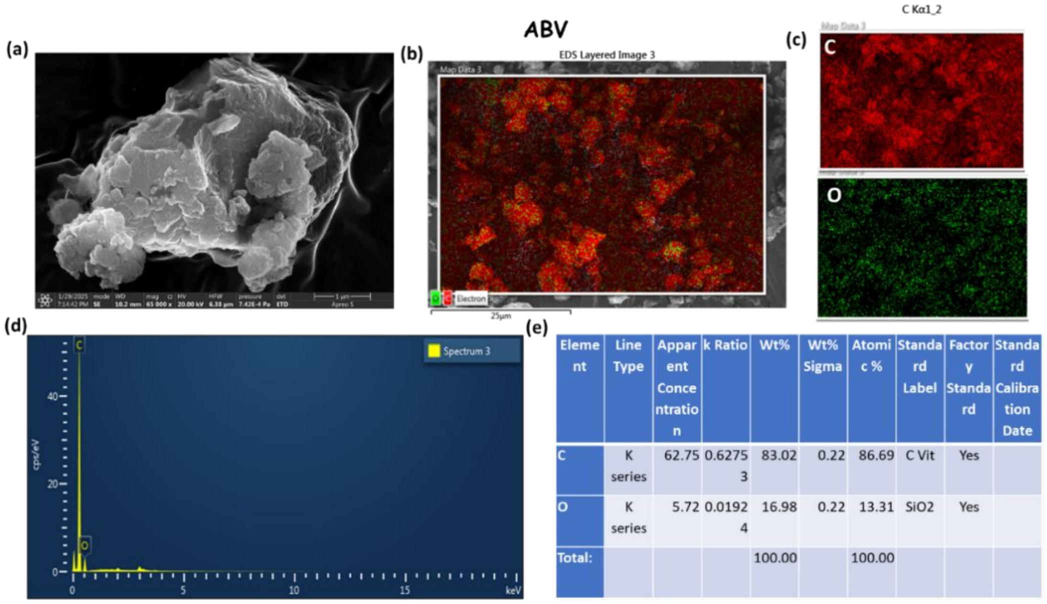

Element	Line Type	Apparent Concentration	k Ratio	Wt%	Wt% Sigma	Atomic %	Standard Label	Factory Standard	Standard Calibration Date
C	K series	62.75	0.62753	83.02	0.22	86.69	C Vit	Yes	
O	K series	5.72	0.01924	16.98	0.22	13.31	SiO2	Yes	
Total:				100.00		100.00			

Figure S22: (a) SEM images, (b) elemental mapping, (c) individual colour map of carbon, (d) EDX plot, and (e) % content of elements, for **ABV** polymer.

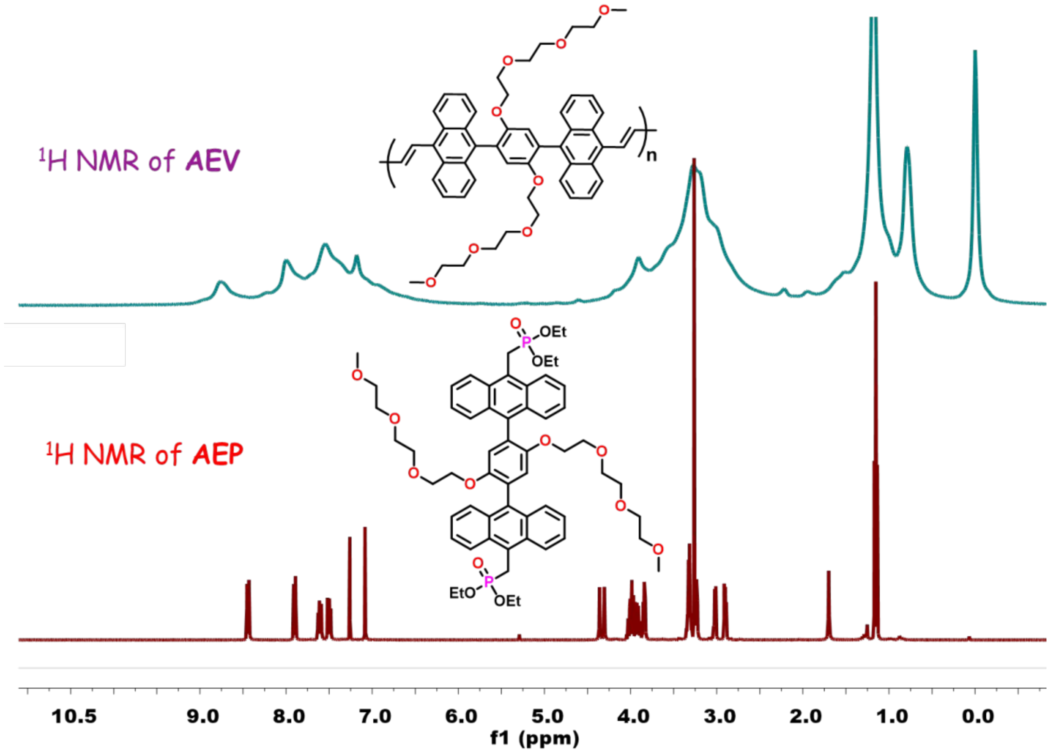

Figure S23: Stacked ^{1}H NMR (400 MHz, $CDCl_3$) spectra of **AEV** polymer (up) and corresponding monomer **AEP** (down).

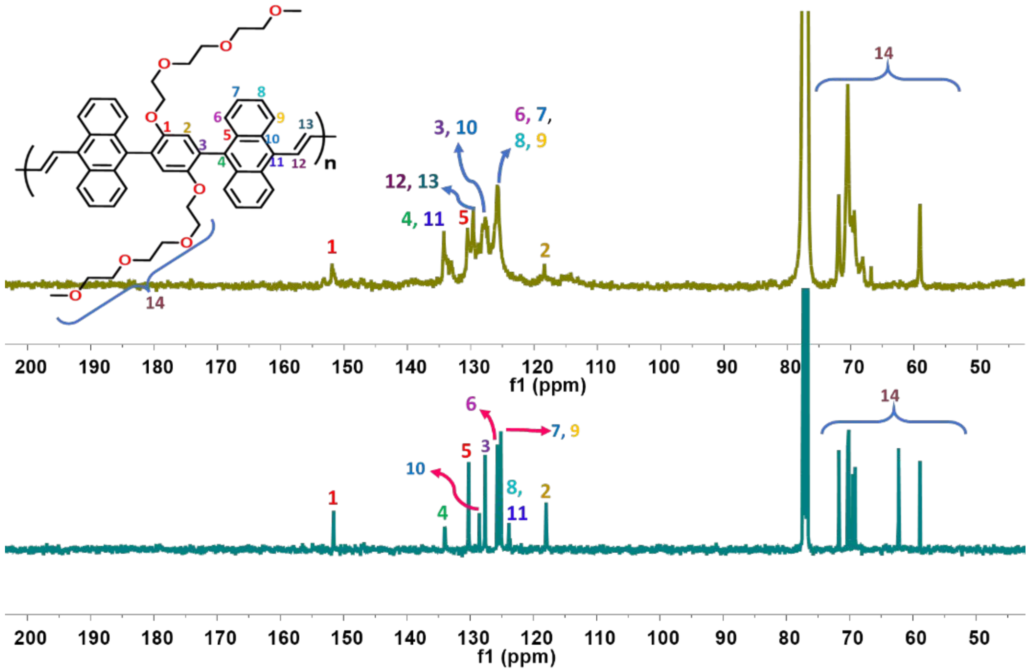

Figure S24: Stacked ^{13}C NMR (101 MHz, $CDCl_3$) spectra of **AEV** polymer (up) and corresponding monomer **AEP** (down).

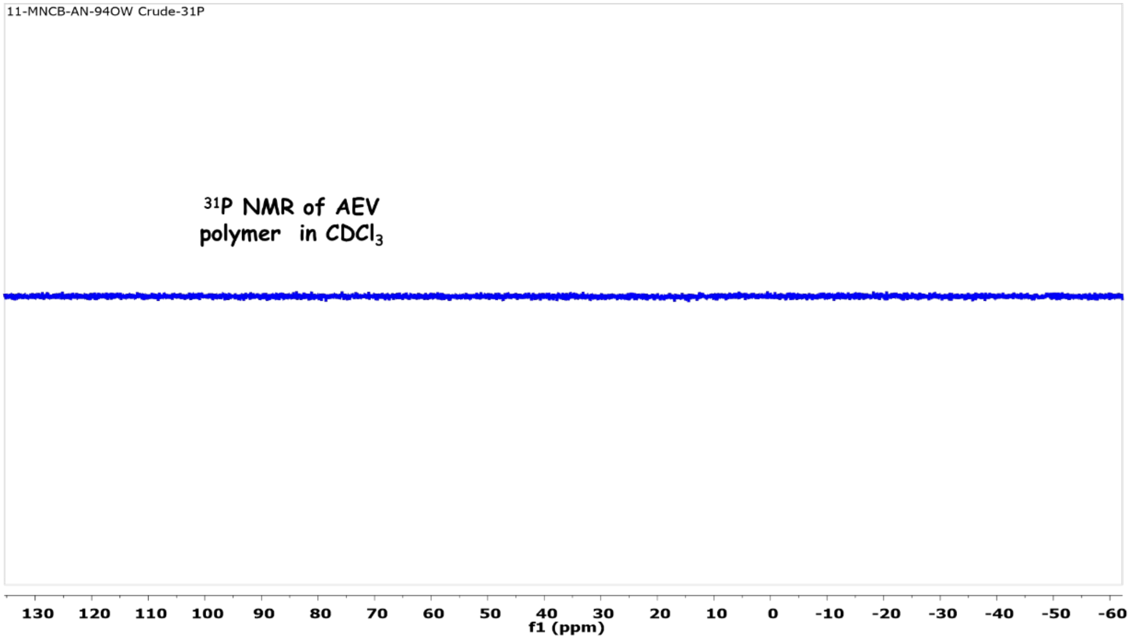

Figure S25: ^{31}P NMR (162 MHz, $CDCl_3$) spectra of **AEV** polymer

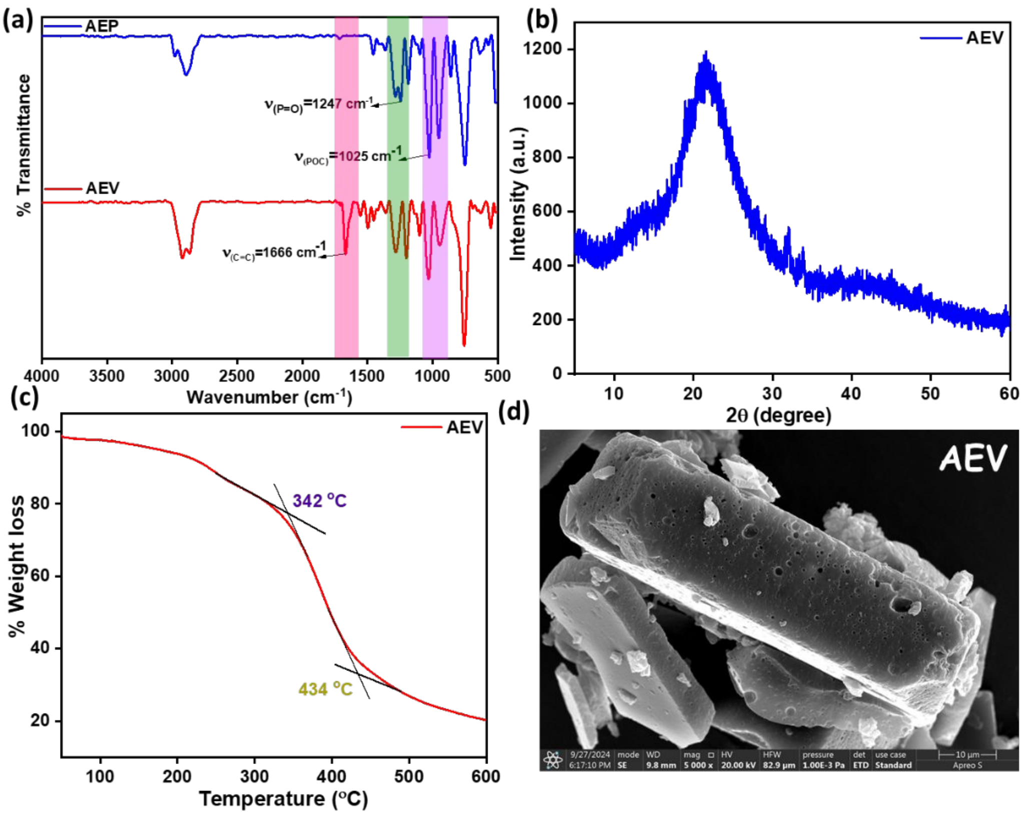

Figure S26: (a) FT-IR spectra, (b) PXRD pattern, (c) TGA diagram and (d) SEM images for **AEV** polymer.

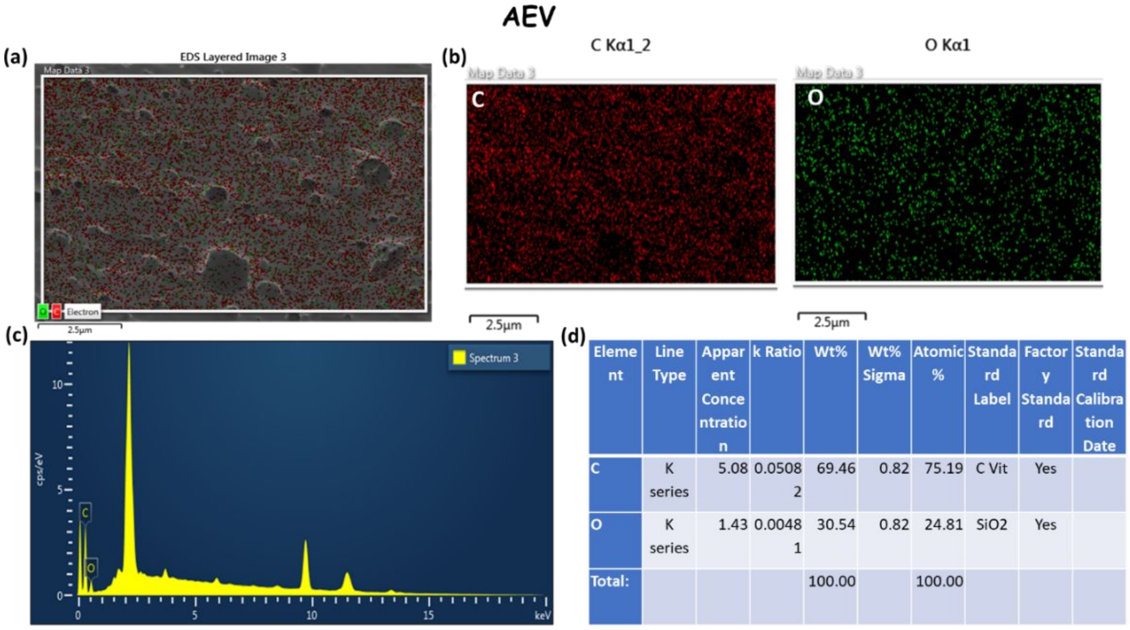

Element	Line Type	Apparent Concentration	k Ratio	Wt%	Wt% Sigma	Atomic %	Standard Label	Factory Standard	Standard Calibration Date
C	K series	5.08	0.05082	69.46	0.82	75.19	C Vit	Yes	
O	K series	1.43	0.00481	30.54	0.82	24.81	SiO2	Yes	
Total:				100.00		100.00			

Figure S27: (a) Elemental mapping, (b) individual colour map of carbon, (c) EDX plot, and (e) % content of elements, for **AEV** polymer.

17. All absorbance data:

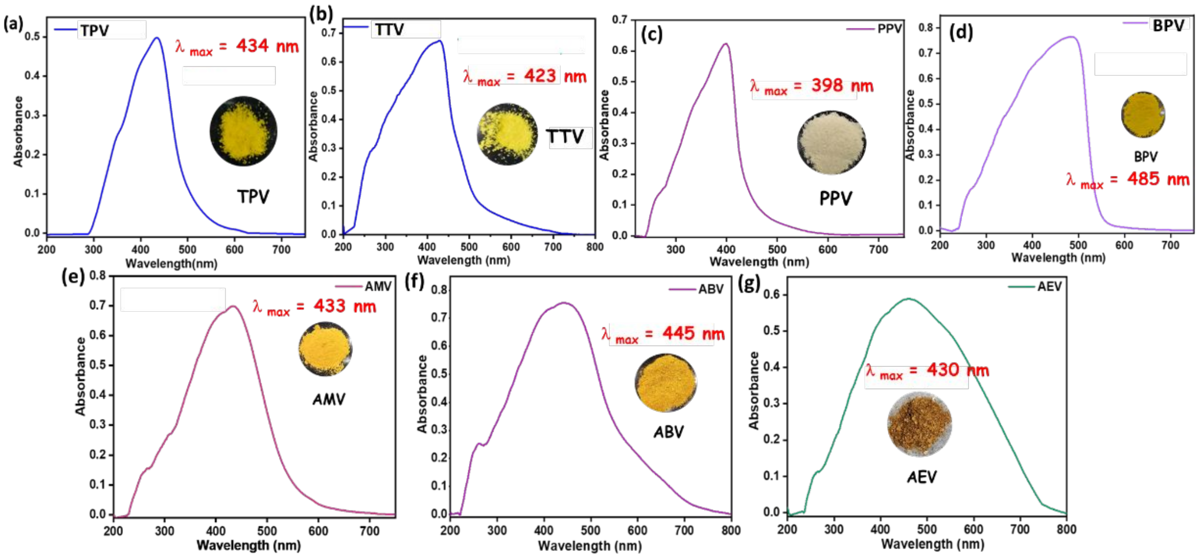

Figure S28: Solid absorbance for (a) **TPV**, (b) **TTV**, (c) **PPV**, (d) **BPV**, (e) **AMV**, (f) **ABV**, and (g) **AEV**.

18. All optical band gap plots:

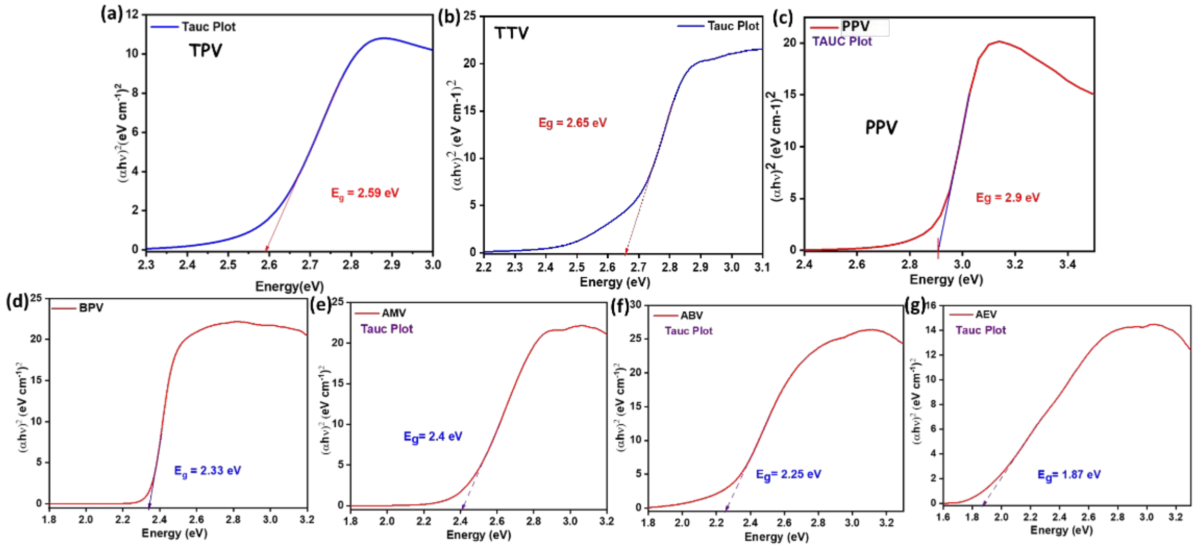

Figure S29: Optical band gap for (a) **TPV**, (b) **TTV**, (c) **PPV**, (d) **BPV**, (e) **AMV**, (f) **ABV** and (g) **AEV**, determined from the Kubelka-Munk transformed reflectance spectra.

19. All emission plots

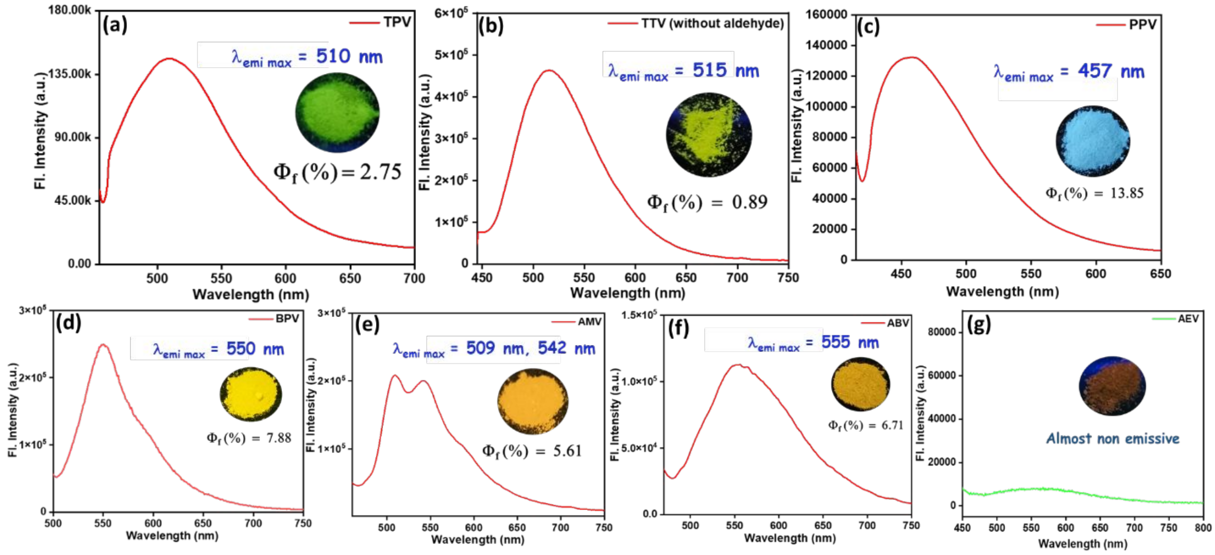

Figure S30: Solid emission for (a) **TPV**, (b) **TTV**, (c) **PPV**, (d) **BPV,** (e) **AMV**, (f) **ABV**, and (g) **AEV.**

20. All lifetime plots:

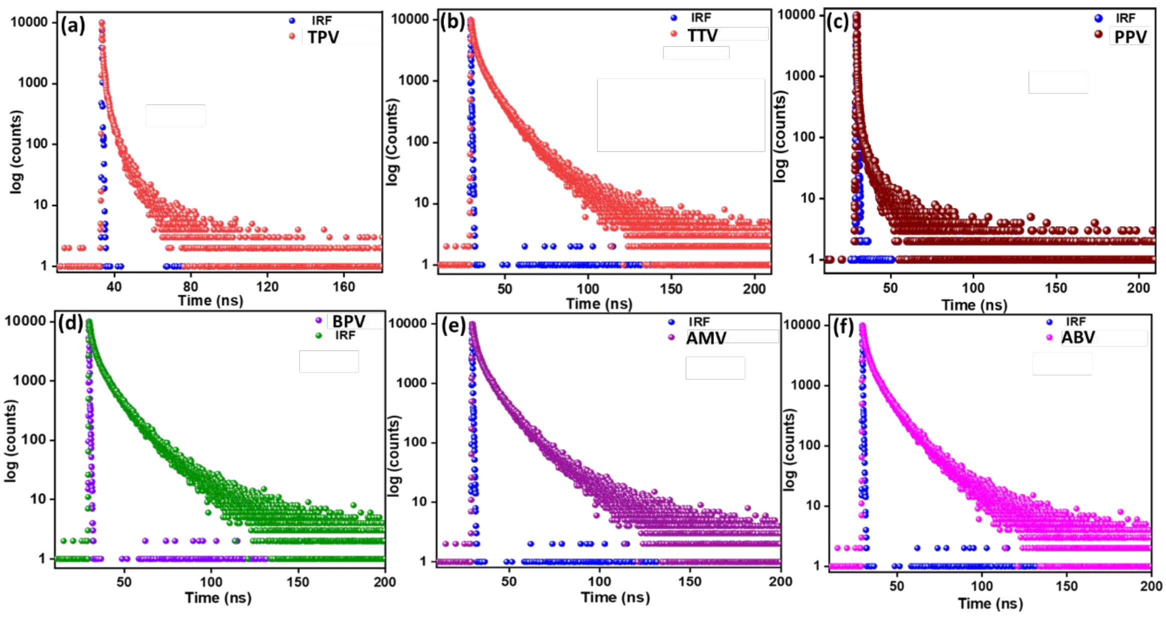

Figure S31: Lifetime plot for (a) **TPV**, (b) **TTV**, (c) **PPV**, (d) **BPV**, (e) **AMV,** and (f) **ABV**.

Table S3: Table for optical band gap.

1-D Polymer	Optical Band Gap (eV)	2-D Polymer	Optical Band Gap (eV)
**BPV**	2.33	**TPV**	2.59
**AMV**	2.4	**TTV**	2.65
**ABV**	2.25	**PPV**	2.9
**AEV**	1.87		

Table S4: Table for absorbance, emission and absolute quantum yield.

**1-D Polymer**	λ (abs max) nm	λ (emi max) nm	Φ_f (%)	**2-D Polymer**	λ (abs max) nm	λ (emi max) nm	Φ_f (%)
**BPV**	485	550	7.88	**TPV**	434	510	2.75
**AMV**	433	509, 542	5.61	**TTV**	423	515	0.89
**ABV**	445	555	6.71	**PPV**	398	457	13.85
**AEV**	430	-	-				

Table S5: Table for absolute quantum yield, average lifetime, radiative and non-radiative rate constant.

**1-D Polymer**	Φ_f (%)	<τ> (ns)	Radiative rate constant (k_r $\times 10^7$ s^{-1})	Non-radiative rate constant (k_{nr} $\times 10^7$ s^{-1})	**2-D Polymer**	Φ_f (%)	<τ> (ns)	Radiative rate constant (k_r $\times 10^7$ s^{-1})	Non-radiative rate constant (k_{nr} $\times 10^7$ s^{-1})
**BPV**	7.88	0.232	33.9	397	**TPV**	2.75	0.44	6.25	221
**AMV**	5.61	0.209	26.8	451.6	**TTV**	0.89	0.244	3.65	406
**ABV**	6.71	0.624	10.75	149.5	**PPV**	13.85	0.172	80.5	500.8
**AEV**	-	-	-	-					

Radiative rate constant (k_r) = Φ_f / τ (s^{-1}).

Non-radiative rate constant (k_{nr}) = $(1-\Phi_f) / \tau$ (s^{-1}).

Table S6: Table for average lifetime

**Polymers**	α_1	α_2	α_3	α_4	τ_1	τ_2	τ_3	τ_4	<τ> (ns)	χ^2
**TPV**	0.89	0.1	0.01	-	0.21	1.69	7.97	-	0.44	1.01
**TTV**	0.064	0.924	0.01	-	1.638	0.082	5.88	-	0.244	0.99

**PPV**	0.018	0.0016	0.98	-	1.64	10.72	0.127	-	0.172	1.05
**BPV**	1.899	0.499	1.36	0.5	0.91	0.232	6.68	0.232	0.232	1
**AMV**	0.948	0.05	-	-	0.136	1.55	-	-	0.209	1.02
**ABV**	0.788	0.211	-	-	0.357	1.618	-	-	0.624	1.06

21. Photo and Chemical Stability Experiment:

The solid powder was irradiated inside the Fluorolog-Horiba Scientific instrument using a 450-watt xenon arc lamp, with excitation at its maximum absorbance wavelength for 90 minutes. Light was applied to the sample at 5-second intervals throughout the experiment.

We chose one linear (**BPV**) and one 2-D polymer (**TPV**) to check chemical stability. The solid samples (5 mg) were immersed in 5 mL of 12 M KOH and 5 mL of 12 M HCl_{aq} for 10 days. The samples were neutralised before FTIR measurement. FTIR spectra proved the unchanged chemical identity as well as the stability of the polymers.

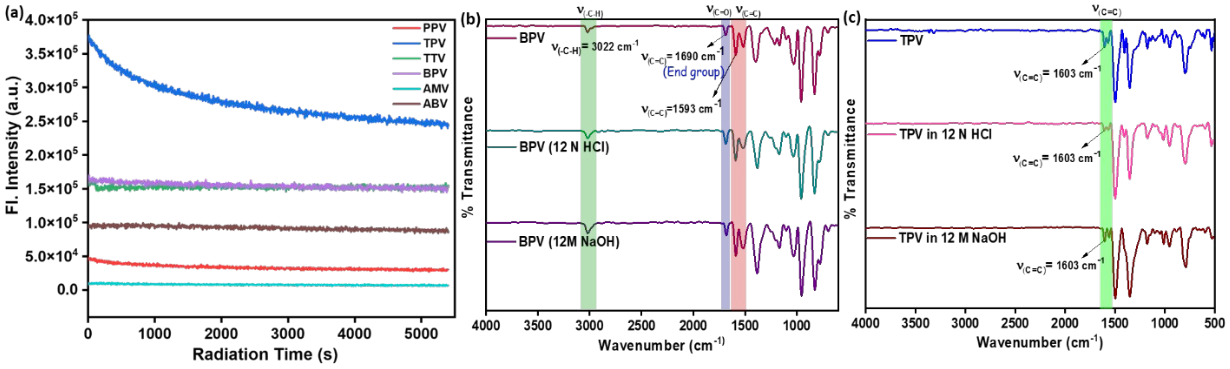

Figure S32: (a) Photostability of the polymers; FT-IR spectra of (b) **BPV**, (c) **TPV**, after immersion in 12 M KOH and 12 M HCl_{aq}.

22. All spectroscopic characterizations

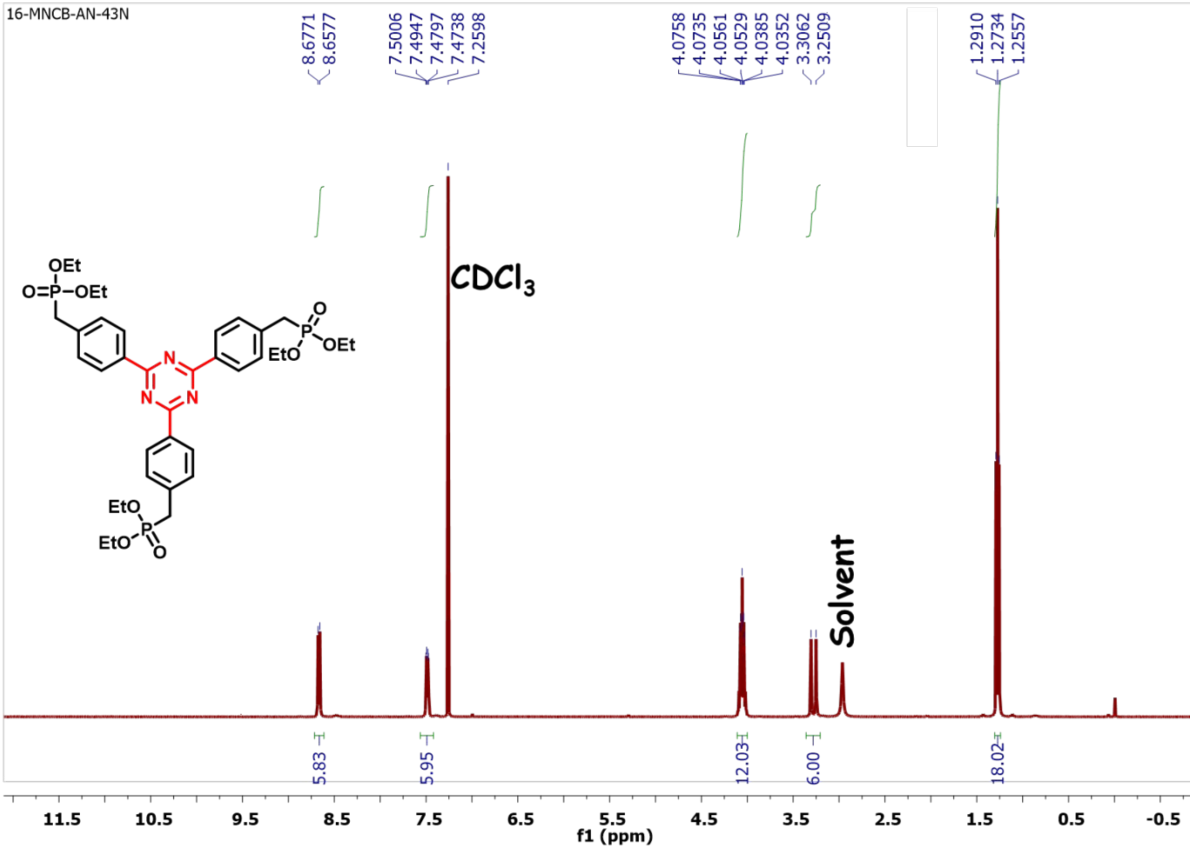

Figure S33: ^{1}H NMR (400 MHz, $CDCl_3$) spectrum of **TPP**

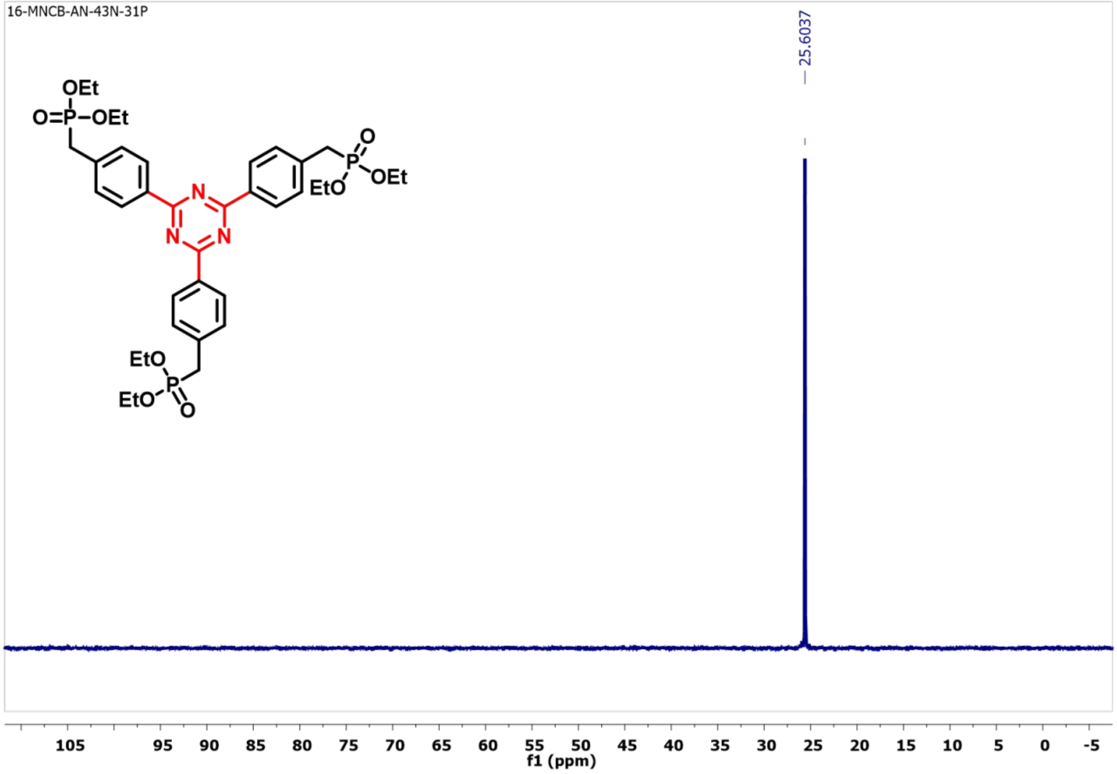

Figure S34: ^{31}P NMR (162 MHz, $CDCl_3$) spectrum of **TPP**

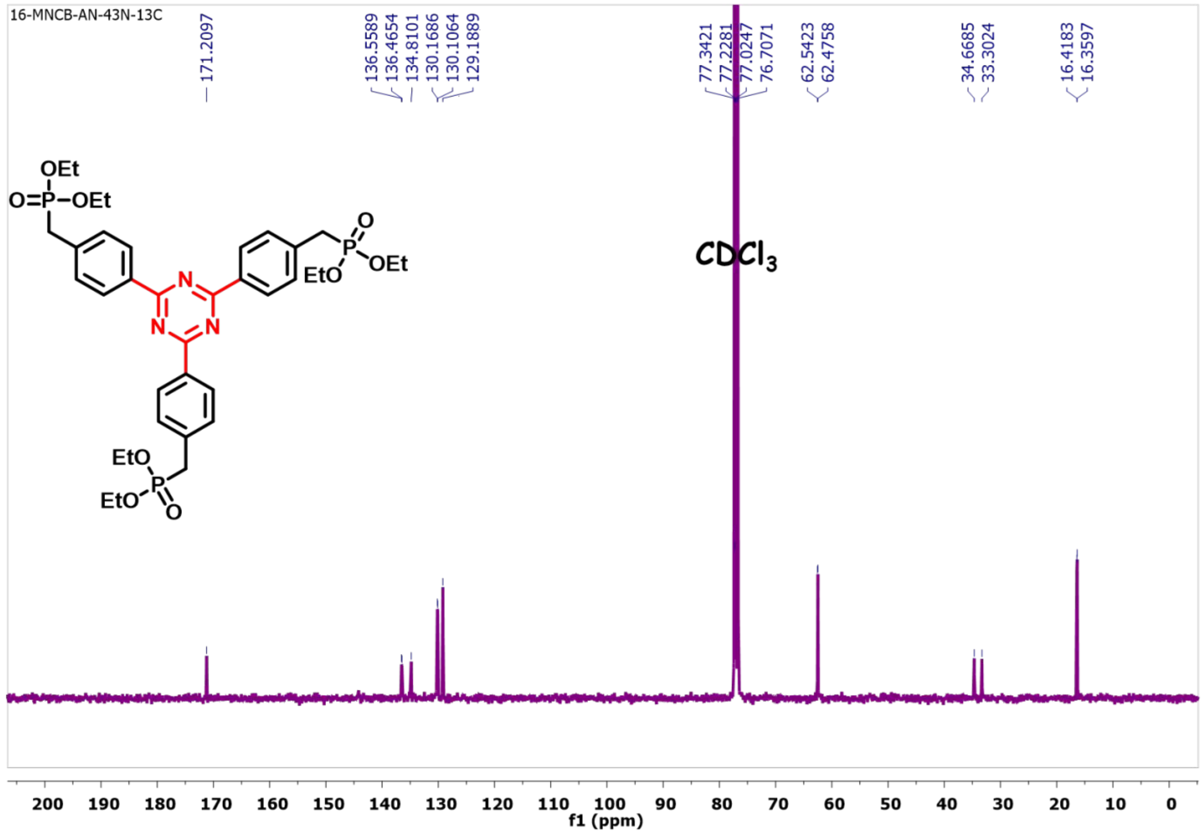

Figure S35: ^{13}C NMR (101 MHz, $CDCl_3$) spectrum of **TPP**

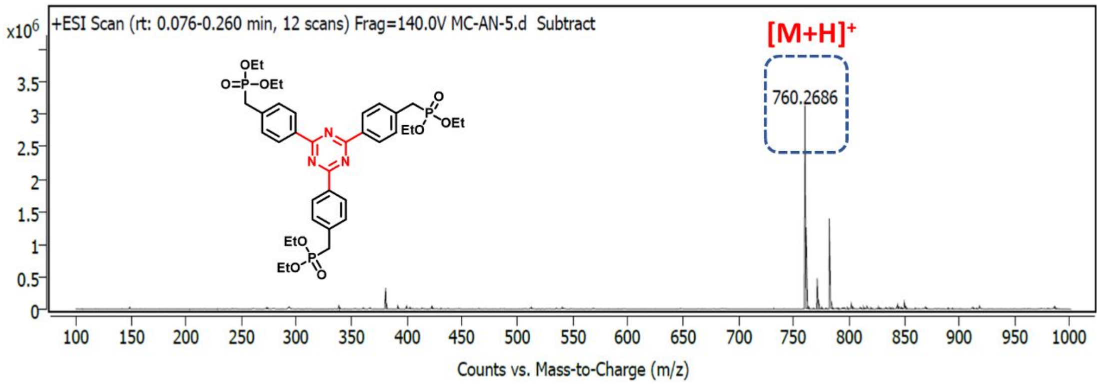

Figure S36: HRMS spectrum of **TPP.**

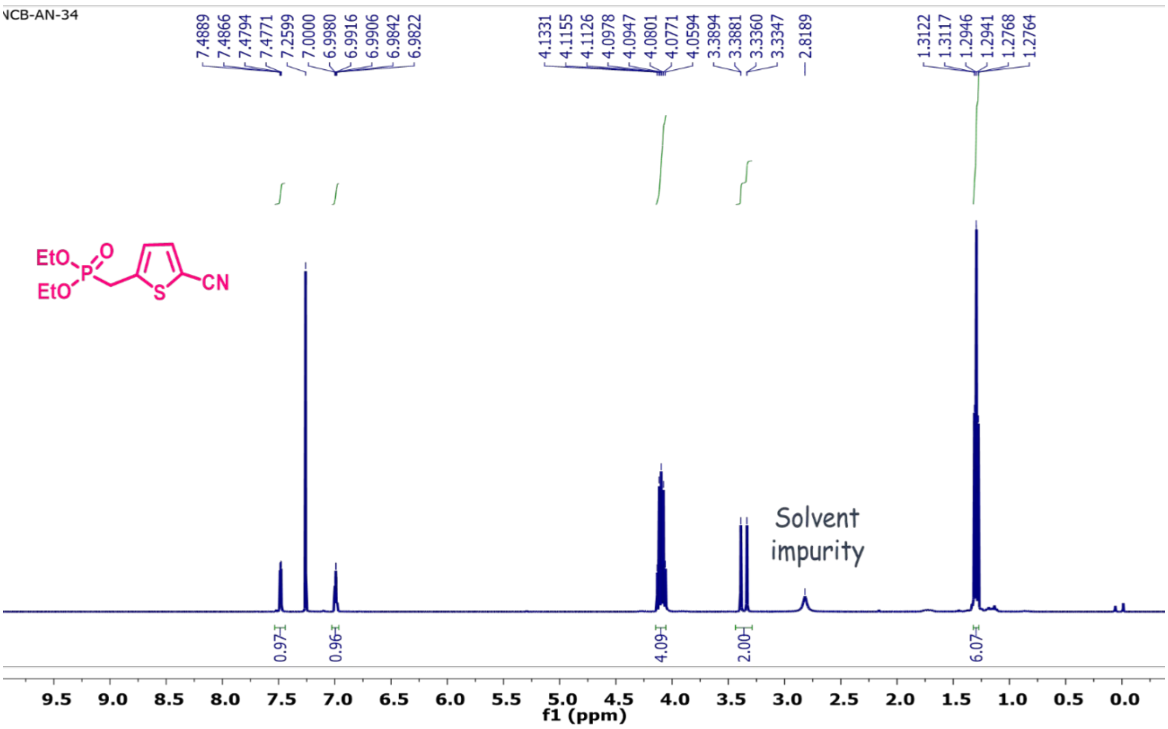

Figure S37: ^{1}H NMR (400 MHz, $CDCl_3$) spectrum of **TNP**

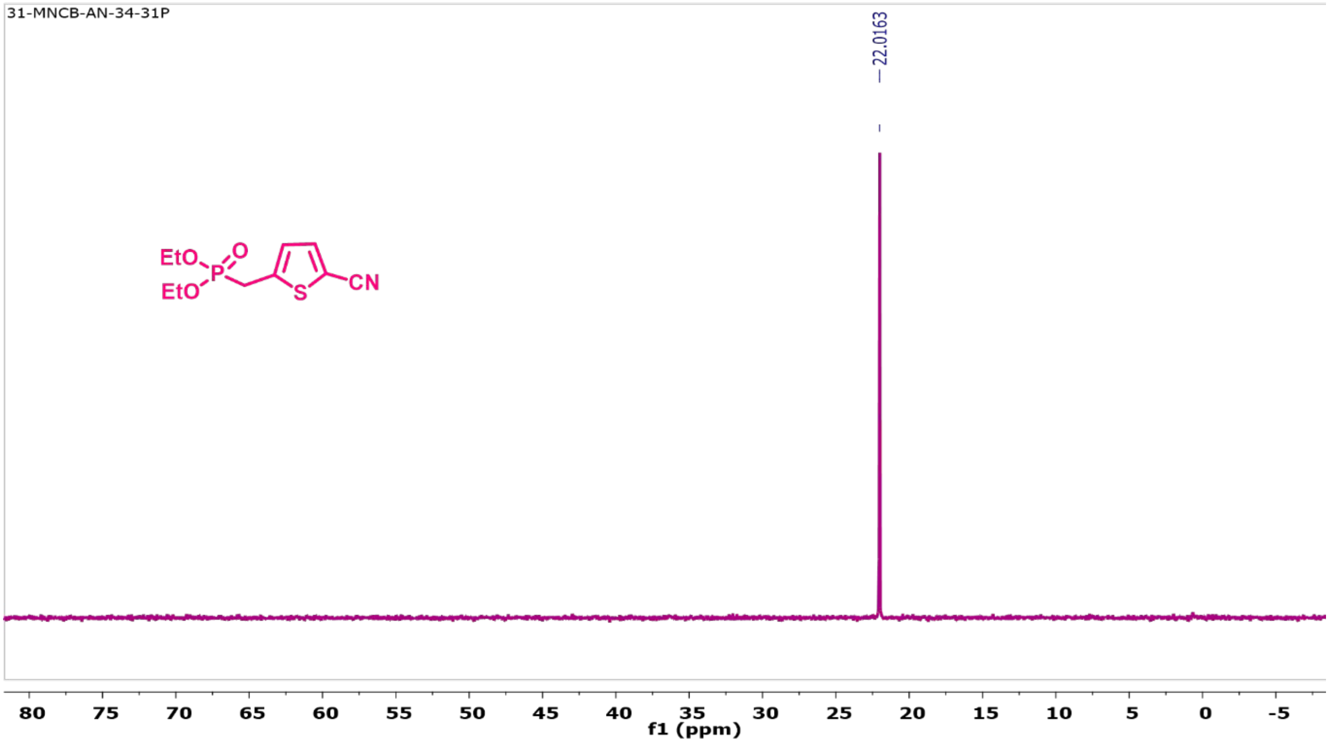

Figure S38: ^{31}P NMR (162 MHz, $CDCl_3$) spectrum of **TNP**

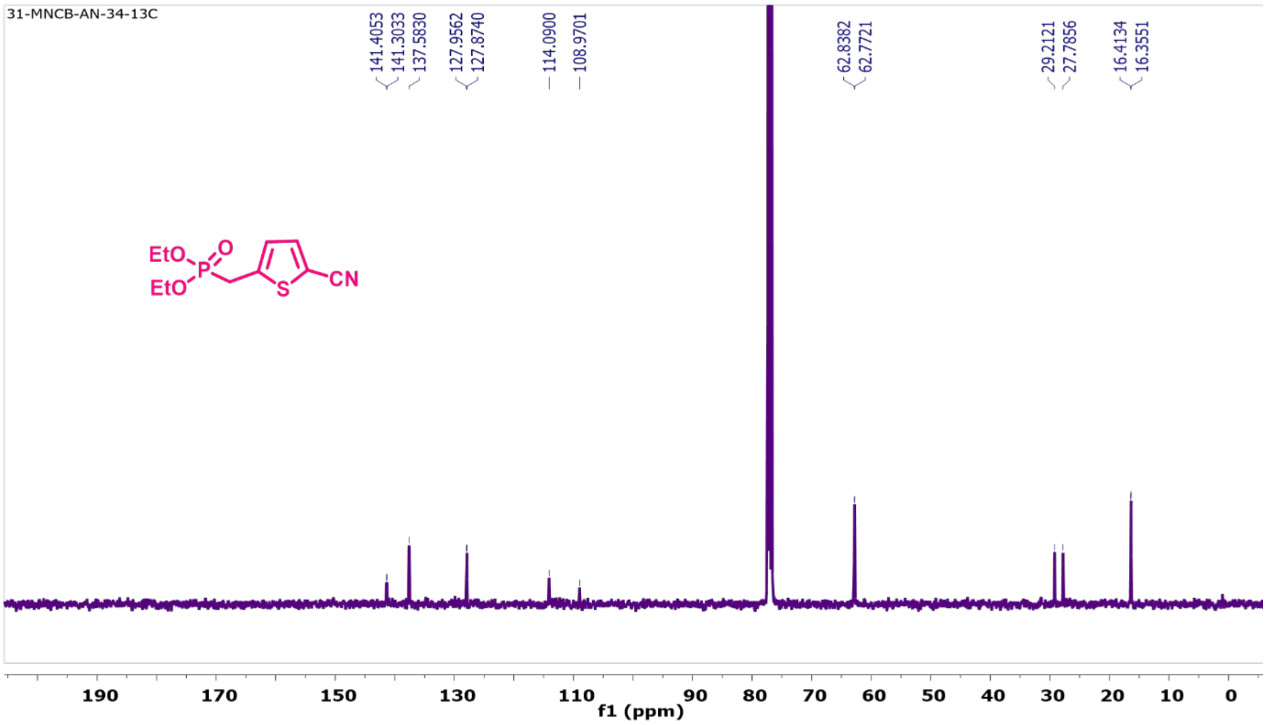

Figure S39: ^{13}C NMR (101 MHz, $CDCl_3$) spectrum of **TNP**

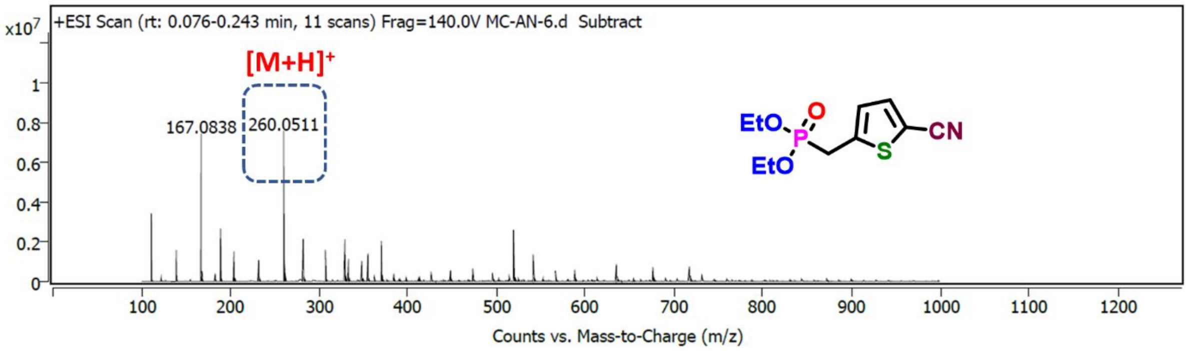

Figure S40: HRMS of **TNP**.

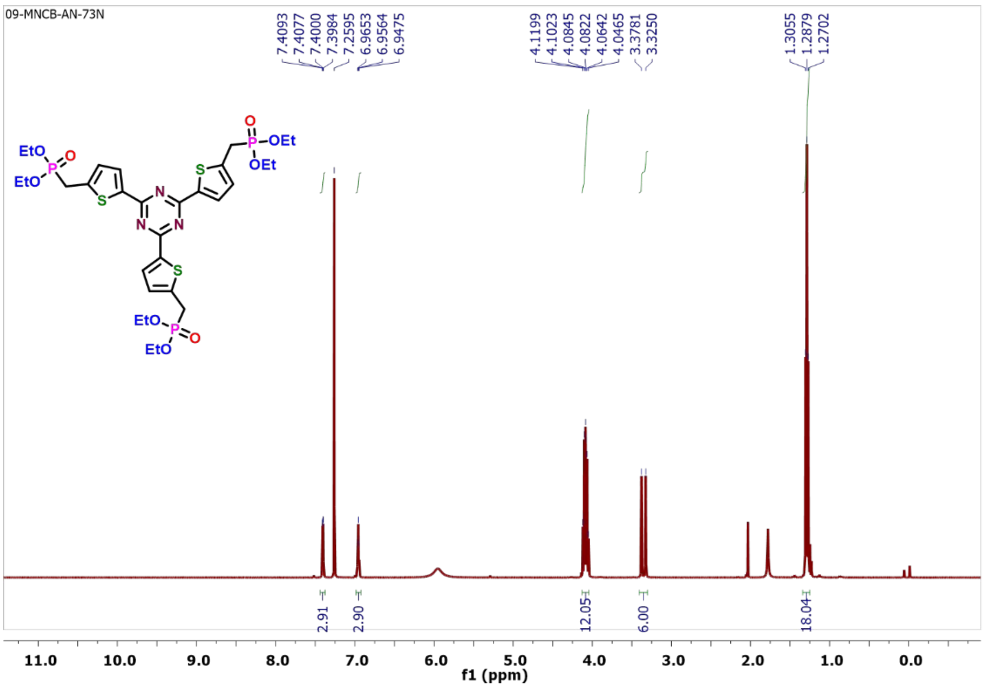

Figure S41: ^{1}H NMR (400 MHz, $CDCl_3$) spectrum of **TTP**

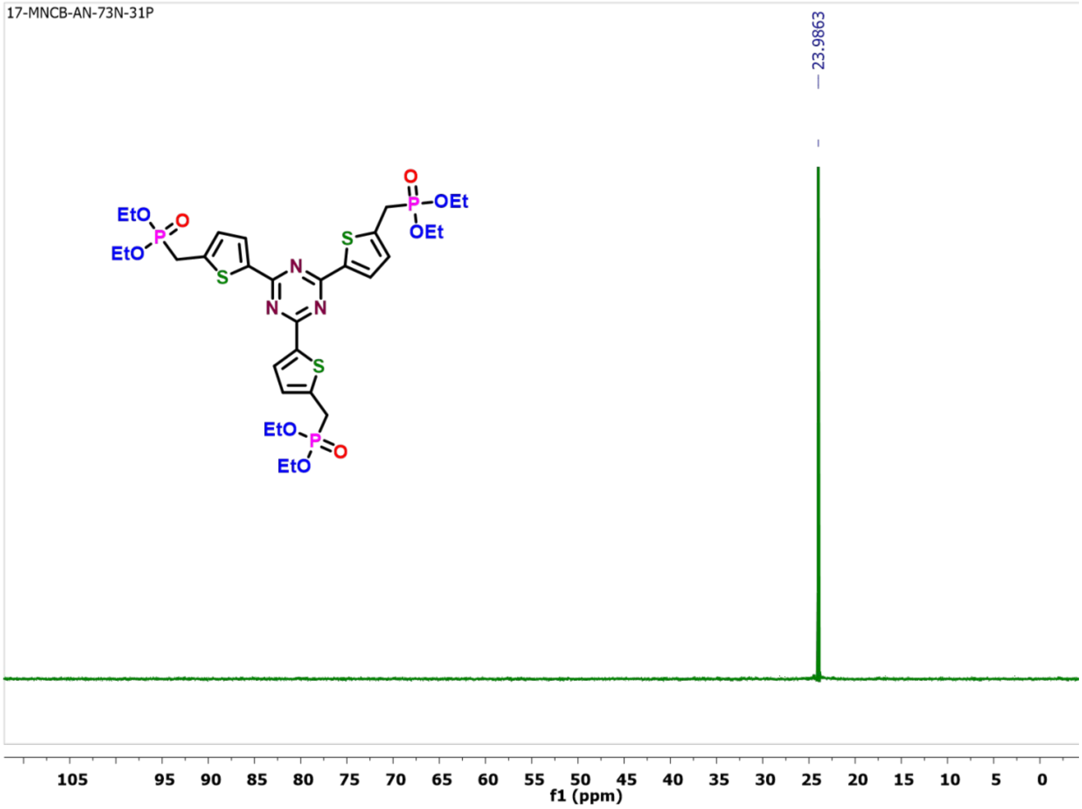

Figure S42: ^{31}P NMR (162 MHz, $CDCl_3$) spectrum of **TTP**

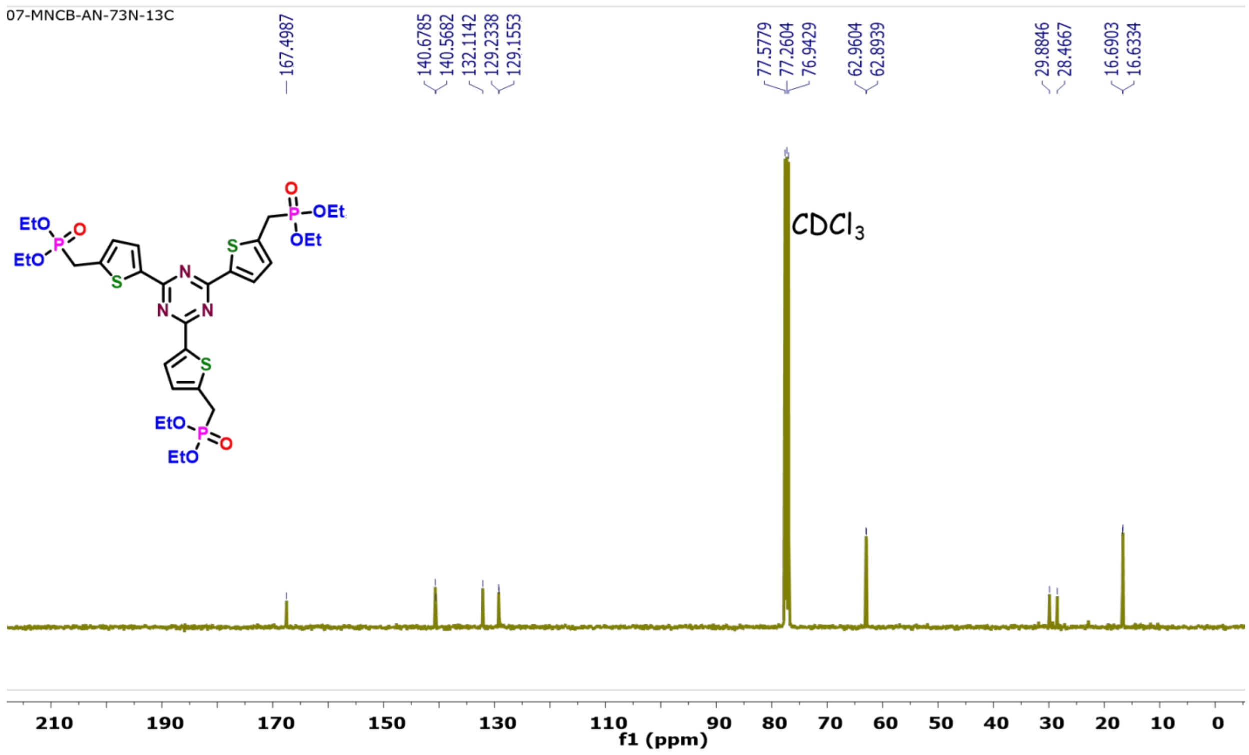

Figure S43: ^{13}C NMR (101 MHz, $CDCl_3$) spectrum of **TTP**

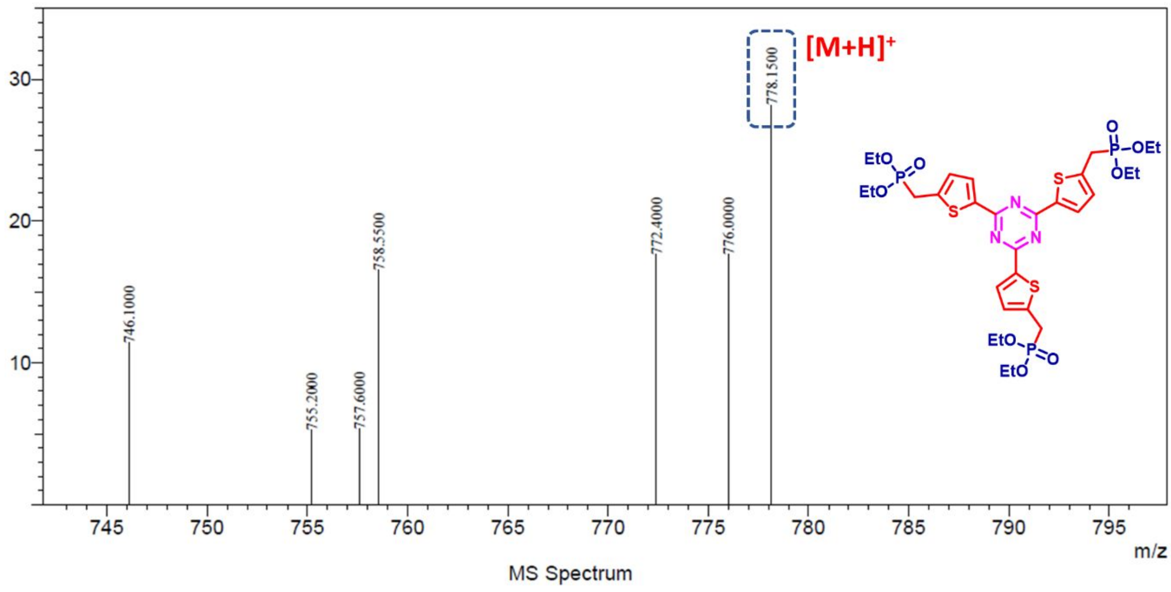

Figure S44: LC-MS (partial) of **TTP.**

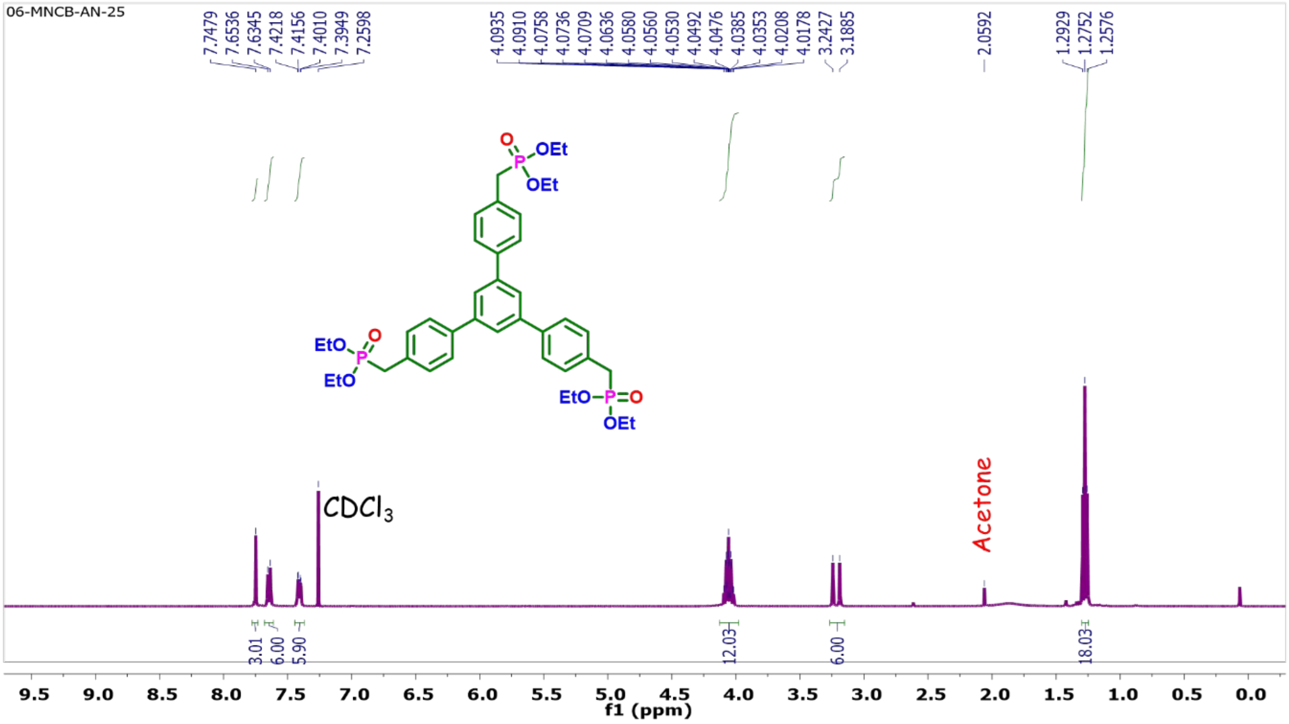

Figure S45: ^{1}H NMR (400 MHz, $CDCl_3$) spectrum of **PPP**

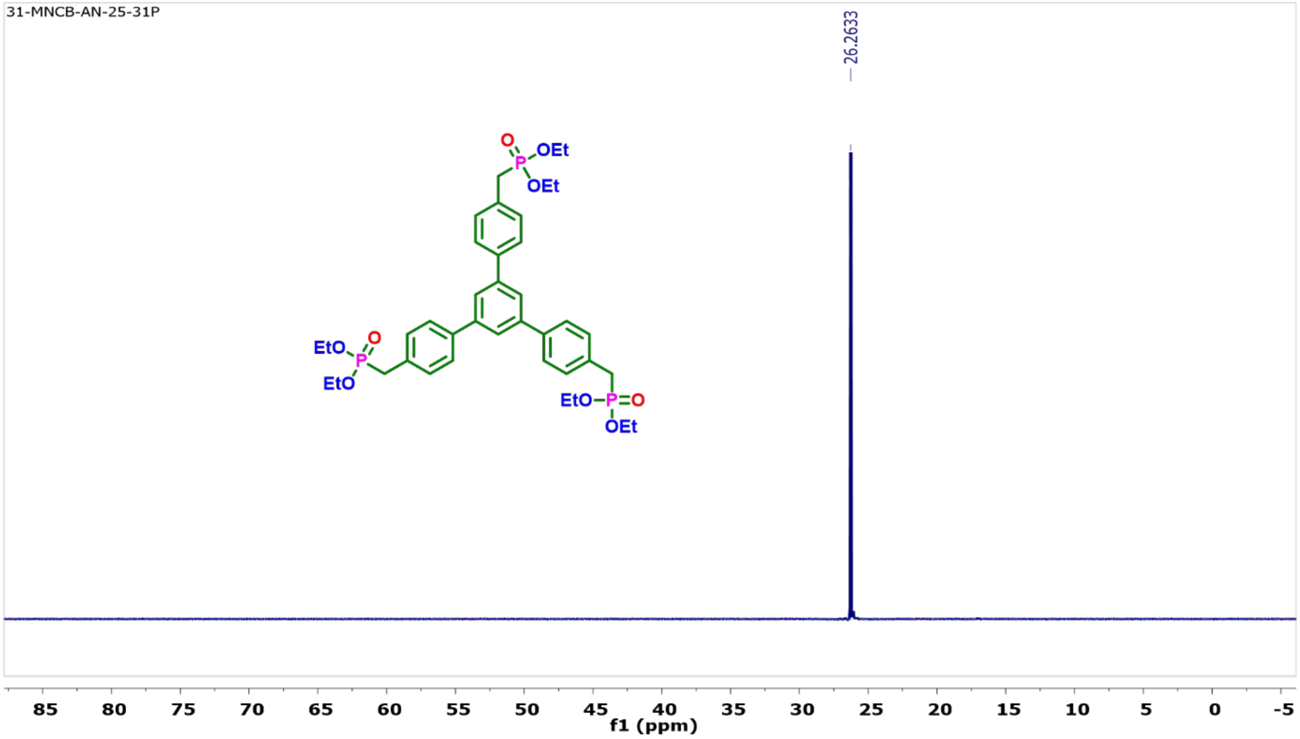

Figure S46: ^{31}P NMR (162 MHz, $CDCl_3$) spectrum of **PPP**

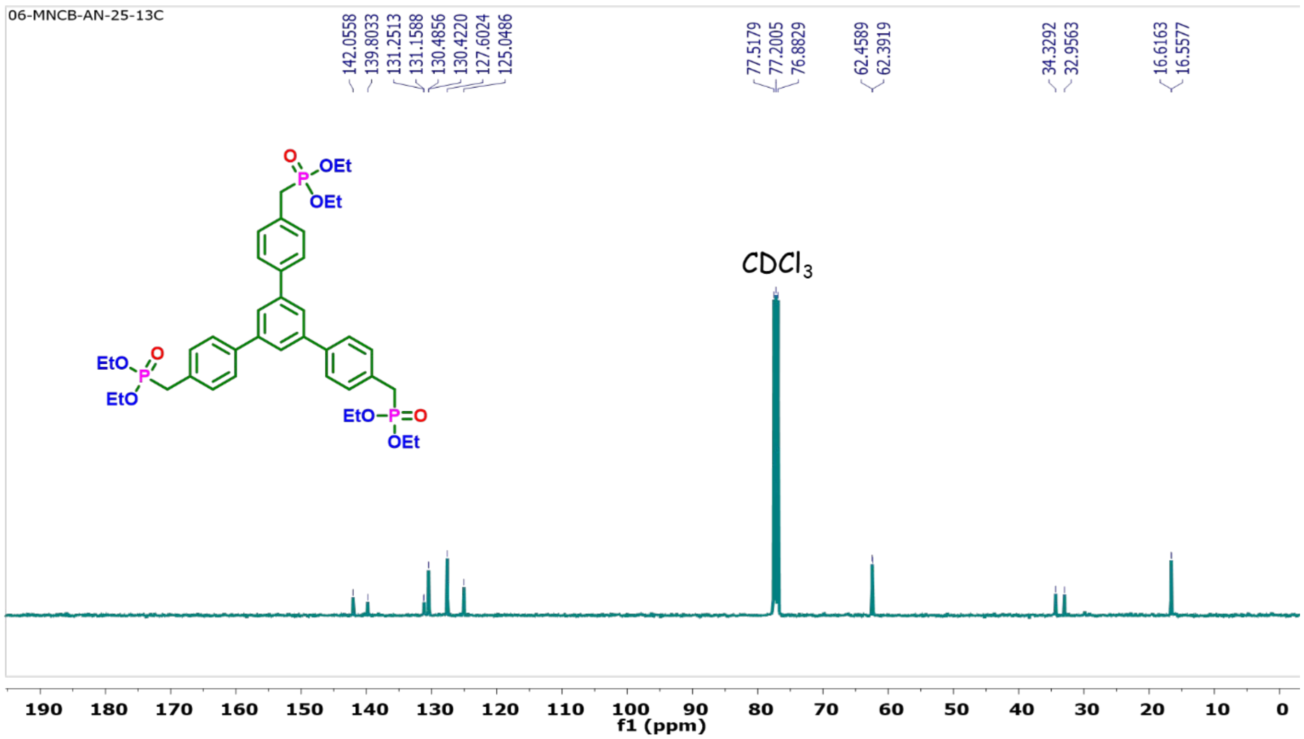

Figure S47: ^{13}C NMR (101 MHz, $CDCl_3$) spectrum of **PPP**

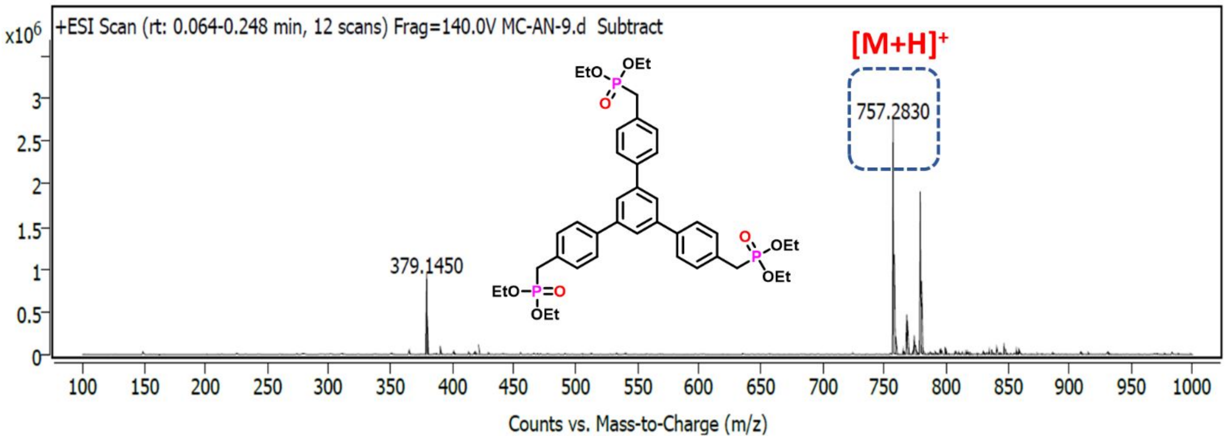

Figure S48: HRMS of **PPP.**

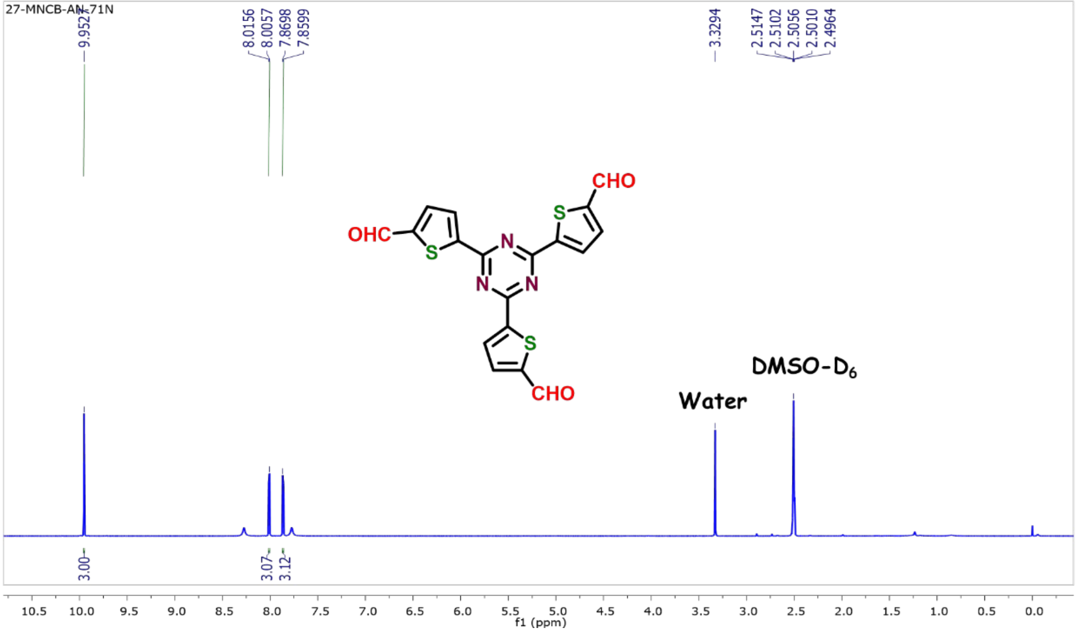

Figure S49: ^{1}H NMR (400 MHz, DMSO-d^{6}) spectrum of **TTA**

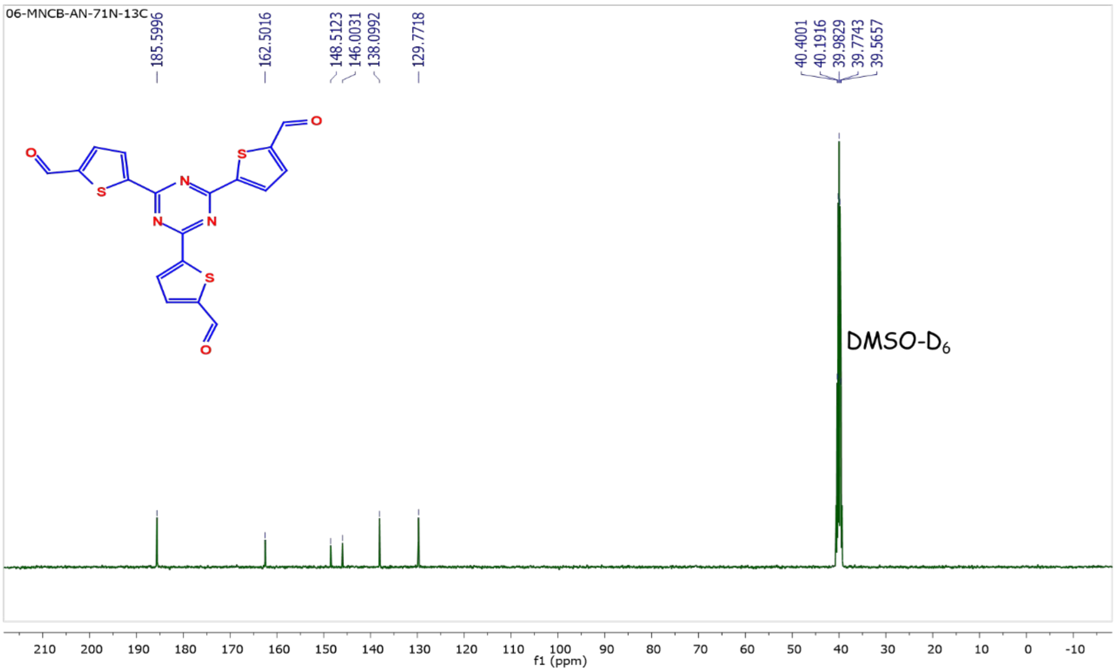

Figure S50: ^{13}C NMR (101 MHz, DMSO-d^{6}) spectrum of **TTA**

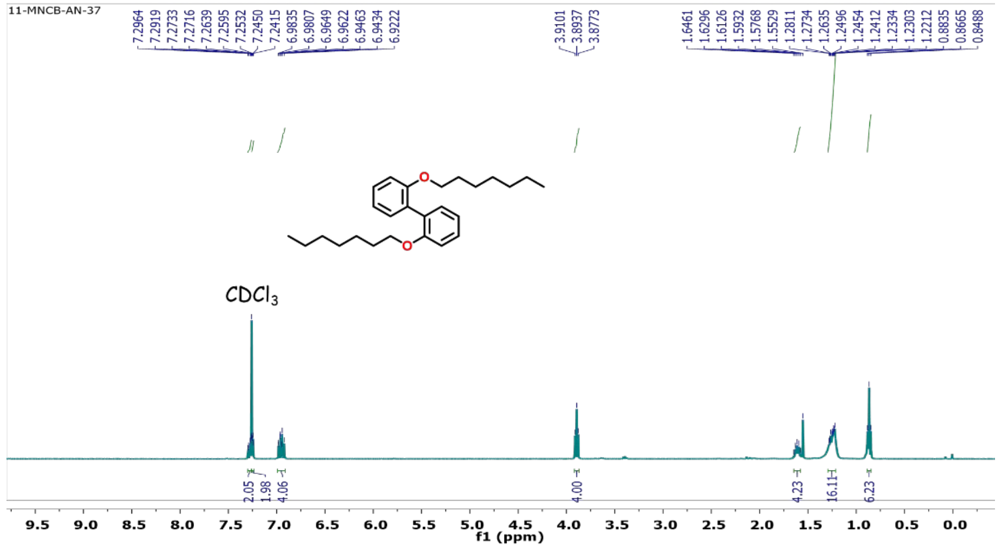

Figure S51: ^{1}H NMR (400 MHz, $CDCl_3$) spectrum of 2,2′-diheptylbiphenol

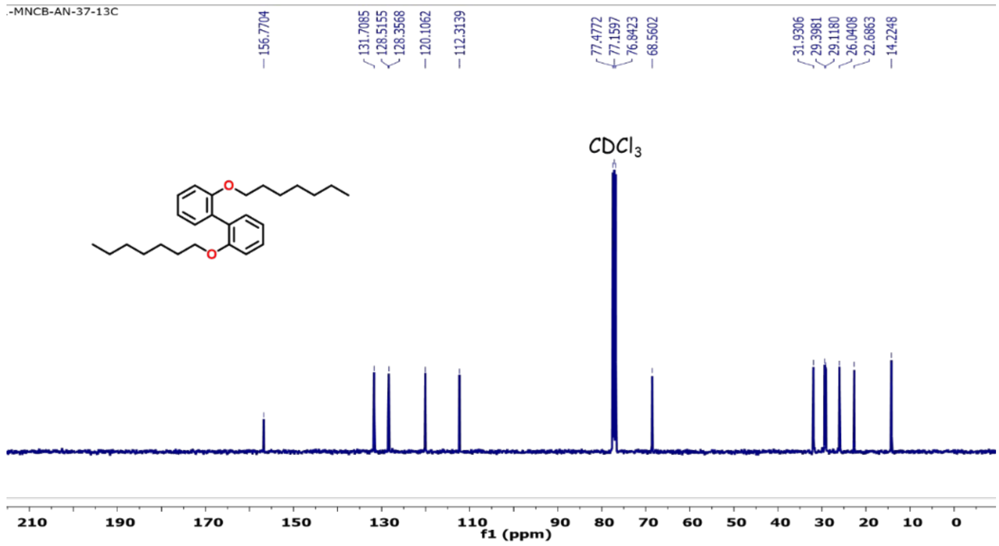

Figure S52: ^{13}C NMR (101 MHz, $CDCl_3$) spectrum of 2,2’-diheptylbiphenol

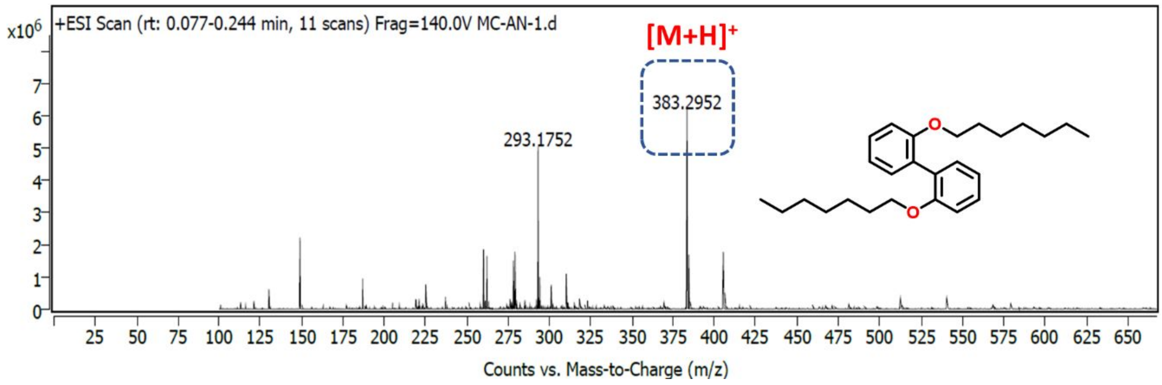

Figure S53: HRMS spectrum of 2,2’-diheptylbiphenol.

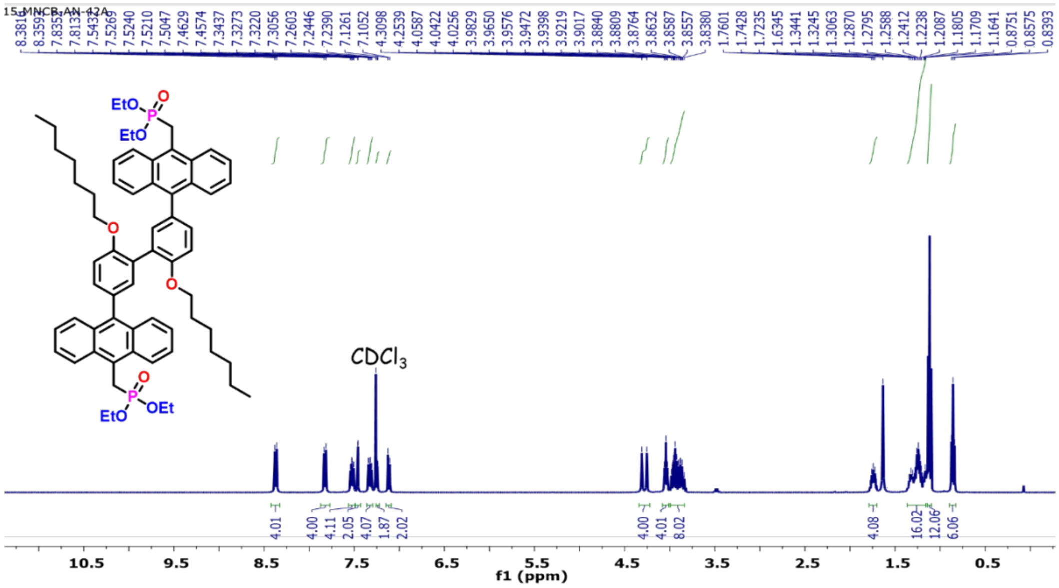

Figure S54: ^{1}H NMR (400 MHz, $CDCl_3$) spectrum of **ABP**

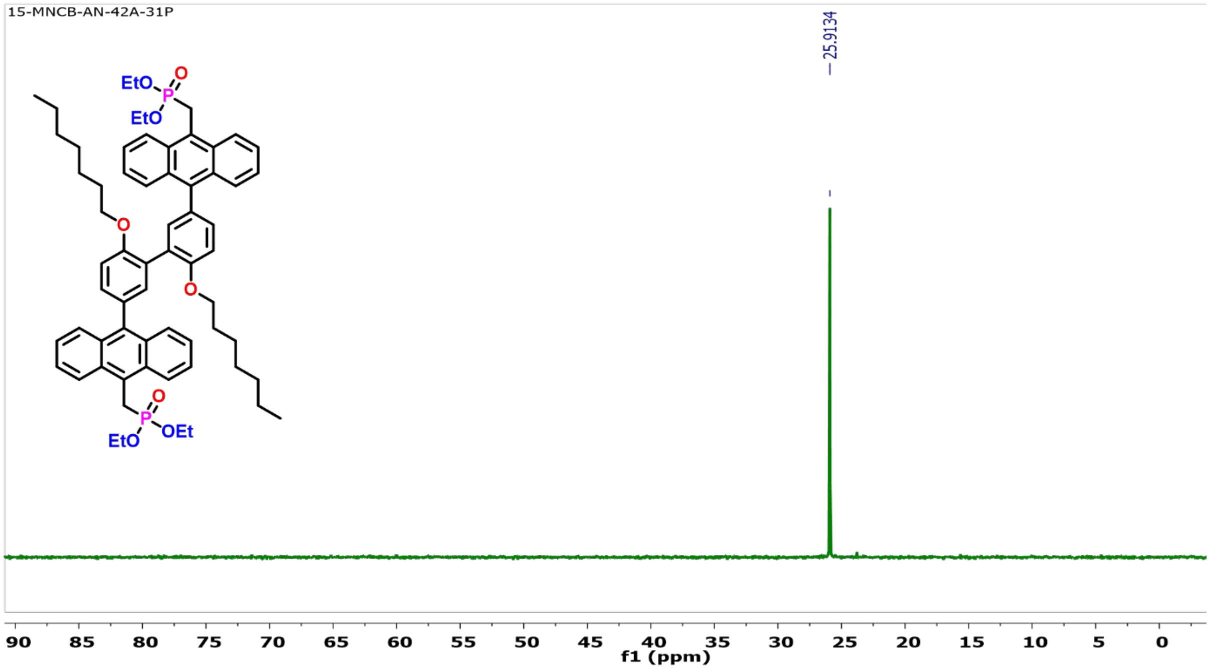

Figure S55: ^{31}P NMR (162 MHz, $CDCl_3$) spectrum of **ABP**

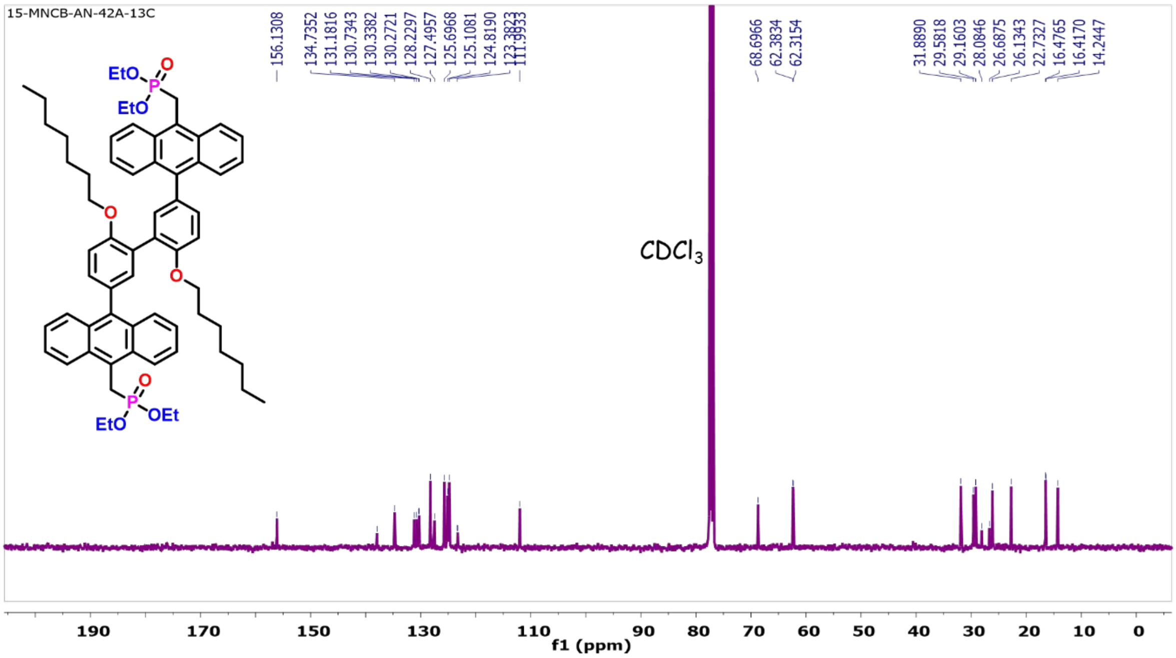

Figure S56: ^{13}C NMR (101 MHz, $CDCl_3$) spectrum of **ABP**

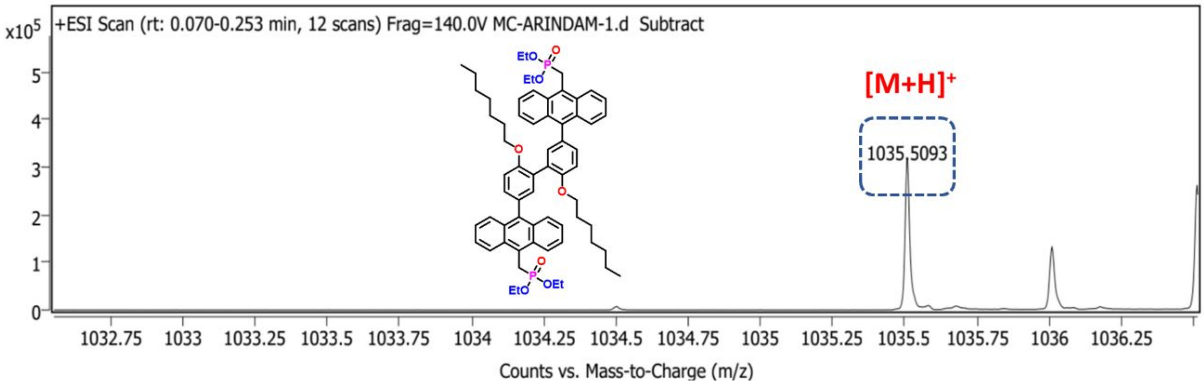

Figure S57: HRMS of **ABP.**

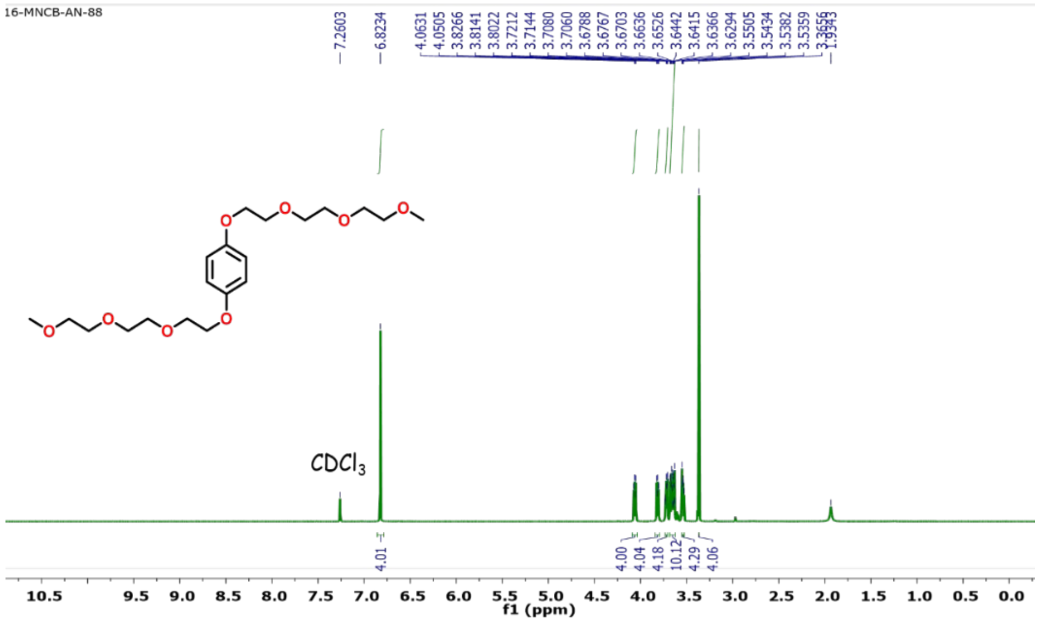

Figure S58: ^{1}H NMR (400 MHz, $CDCl_3$) spectrum of **PEG3-HQ**

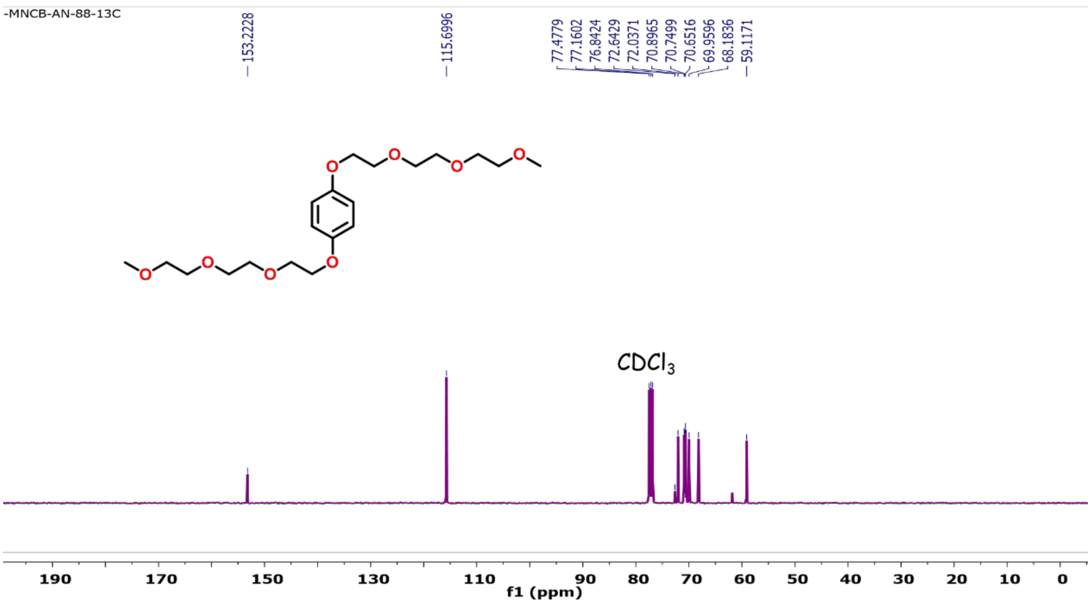

Figure S59: ^{13}C NMR (101 MHz, $CDCl_3$) spectrum of **PEG3-HQ**

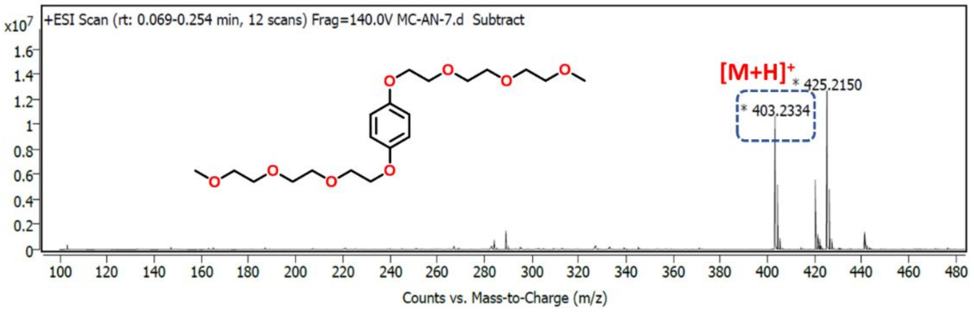

Figure S60: HRMS spectrum of **PEG3-HQ.**

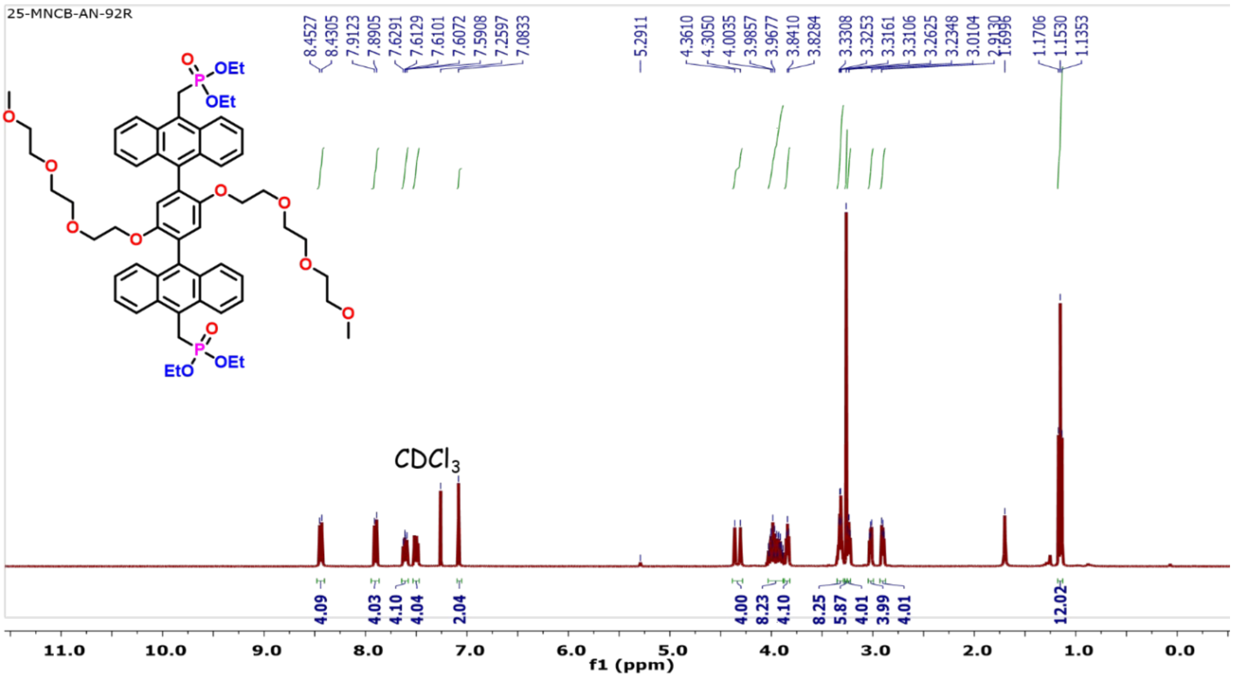

Figure S61: ^{1}H NMR (400 MHz, $CDCl_3$) spectrum of **AEP**

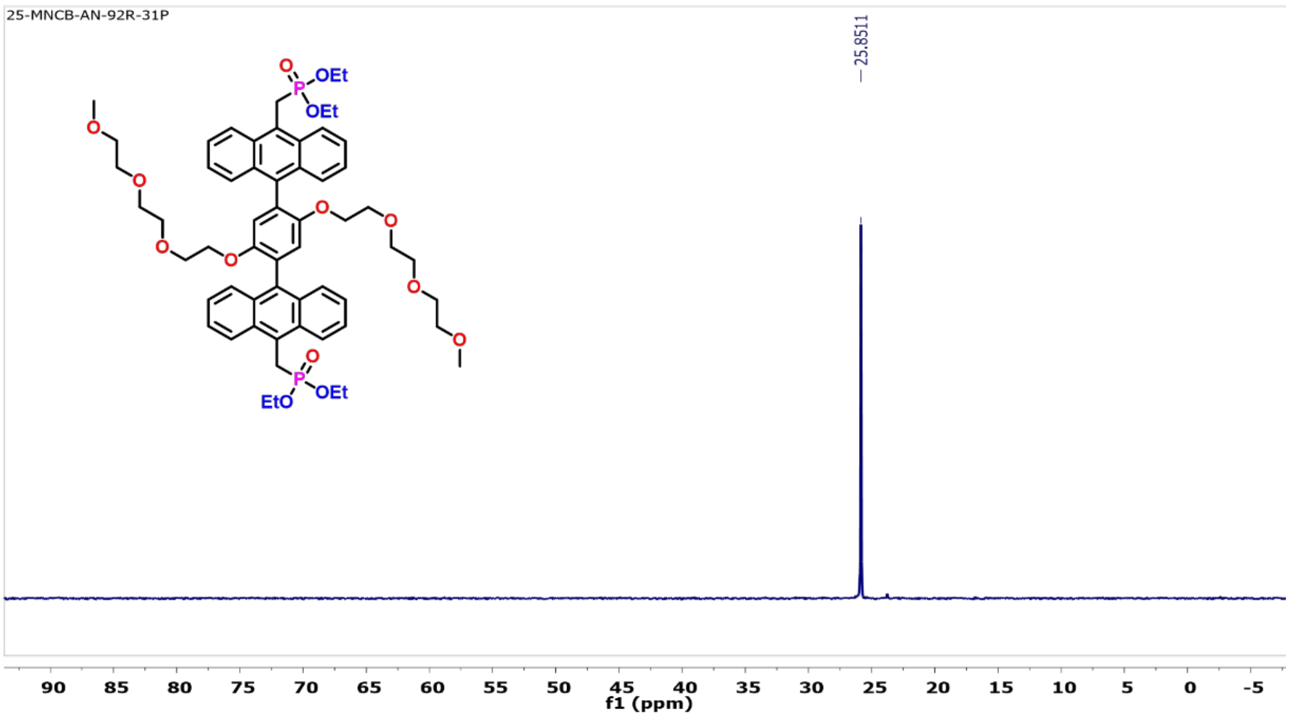

Figure S62: ^{31}P NMR (162 MHz, $CDCl_3$) spectrum of **AEP**

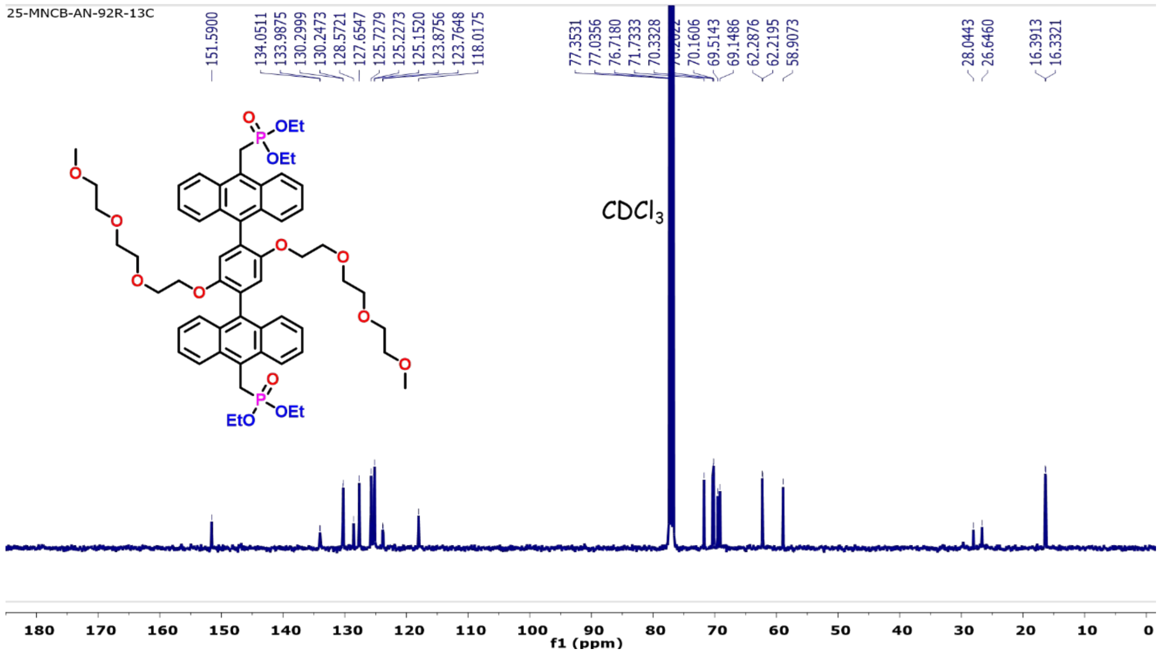

Figure 63: ^{13}C NMR (101 MHz, $CDCl_3$) spectrum of **AEP**

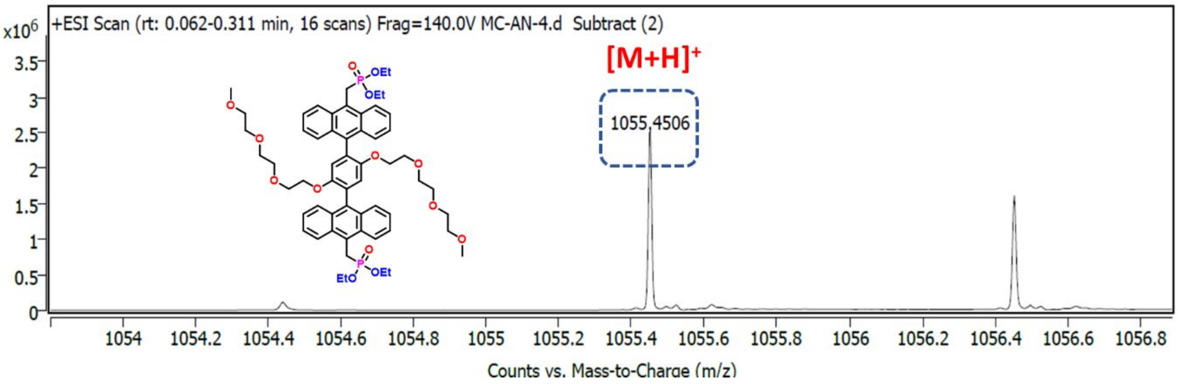

Figure S64: HRMS (partial) of **AEP.**

23. Supplementary References:

1. Nishizawa, T.; Lim, H. K.; Tajima, K.; Hashimoto, K. Highly Uniaxial Orientation in Oligo(p-phenylenevinylene) Films Induced during Wet-Coating Process. *J. Am. Chem. Soc.* **2009**, *131*, 2464–2465.

2. Wang, J.; Wang, S.; Li, X.; Zhu, L.; Meng, Q.; Xiao, Y.; Li, D. Novel Hole Transporting Materials with a Linear π-Conjugated Structure for Highly Efficient Perovskite Solar Cells. *Chem. Commun.* **2014**, *50*, 5829–5832.

3. Prusti, B.; Tripathi, S.; Samanta, P. K.; Chakravarty, M. Vinylene-Linked Conjugated Microporous Polymer Decorated with Electron-Rich Units: A Single-Component White Light Emitting Device. *Adv. Opt. Mater.* **2024**, *12*, 2301746.

4. Huang, W.; Wang, H.; Sun, L.; Li, B.; Su, J.; Tian, H. Propeller-like D–π–A Architectures: Bright Solid Emitters with AIEE Activity and Large Two-Photon Absorption. *J. Mater. Chem. C* **2014**, *2*, 6843–6849.

5. He, Y.; Ma, W.; Yang, N.; Liu, F.; Chen, Y.; Liu, H.; Zhu, X. Efficient Synthesis of Vinylene-Linked Conjugated Porous Networks via the Horner–Wadsworth–Emmons Reaction for Photocatalytic Hydrogen Evolution. *Chem. Commun.* **2021**, *57*, 7557–7560.

6. Gu, Z.; Wang, J.; Shan, Z.; Wu, M.; Liu, T.; Song, L.; Zhang, G. Modulating Electronic Structure of Triazine-Based Covalent Organic Frameworks for Photocatalytic Organic Transformations. *J. Mater. Chem. A* **2022**, *10* (34), 17624–17632.

____________________END____________________
