# Supplementary figures and images for: Easy Access to Vinylene-Linked Conjugated Homopolymers from Phosphonates via an O2‑Mediated Aldehyde-Free Strategy
Source: Org Lett. 2025 Sep 13;27(38):10695–9. doi: 10.1021/acs.orglett.5c03195 (PMC12481555; doi:10.1021/acs.orglett.5c03195)

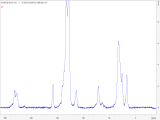

Supplement: Supplementary file 2 [file ol5c03195_si_002.zip › NMR FID Files/ABV/1/pdata/1/thumb.png]
